# Pdia4 regulates β‐cell pathogenesis in diabetes: molecular mechanism and targeted therapy

**DOI:** 10.15252/emmm.201911668

**Published:** 2021-09-20

**Authors:** Tien‐Fen Kuo, Shuo‐Wen Hsu, Shou‐Hsien Huang, Cicero Lee‐Tian Chang, Ching‐Shan Feng, Ming‐Guang Huang, Tzung‐Yan Chen, Meng‐Ting Yang, Si‐Tse Jiang, Tuan‐Nan Wen, Chun‐Yen Yang, Chung‐Yu Huang, Shu‐Huei Kao, Keng‐Chang Tsai, Greta Yang, Wen‐Chin Yang

**Affiliations:** ^1^ Agricultural Biotechnology Research Center Academia Sinica Taipei Taiwan; ^2^ Graduate Institute of Life Science National Defense Medical Center Taipei Taiwan; ^3^ Department of Veterinary Medicine, College of Veterinary Medicine National Chung Hsing University Taichung Taiwan; ^4^ Department of Aquaculture National Taiwan Ocean University Keelung Taiwan; ^5^ Institute of Biotechnology National Taiwan University Taipei Taiwan; ^6^ Institute of Biotechnology National Chung‐Hsing University Taichung Taiwan; ^7^ Molecular and Biological Agricultural Sciences Taiwan International Graduate Program Academia Sinica Taipei Taiwan; ^8^ Graduate Institute of Integrated Medicine China Medical University Taichung Taiwan; ^9^ National Laboratory Animal Center National Applied Research Laboratories Taipei Taiwan; ^10^ Institute of Plant and Microbial Biology Academia Sinica Taipei Taiwan; ^11^ PhD Program in Medical Biotechnology College of Medical Science and Technology Taipei Medical University Taipei Taiwan; ^12^ National Research Institute of Chinese Medicine Ministry of Health and Welfare Taipei Taiwan; ^13^ Department of Life Sciences National Chung Hsing University Taichung Taiwan; ^14^ Institute of Pharmacology National Yang‐Ming University Taipei Taiwan

**Keywords:** diabetes, Pdia4, ROS, β‐cell failure, β‐cells, Metabolism, Molecular Biology of Disease

## Abstract

Loss of β‐cell number and function is a hallmark of diabetes. β‐cell preservation is emerging as a promising strategy to treat and reverse diabetes. Here, we first found that Pdia4 was primarily expressed in β‐cells. This expression was up‐regulated in β‐cells and blood of mice in response to excess nutrients. Ablation of Pdia4 alleviated diabetes as shown by reduced islet destruction, blood glucose and HbA1c, reactive oxygen species (ROS), and increased insulin secretion in diabetic mice. Strikingly, this ablation alone or in combination with food reduction could fully reverse diabetes. Conversely, overexpression of Pdia4 had the opposite pathophysiological outcomes in the mice. In addition, Pdia4 positively regulated β‐cell death, dysfunction, and ROS production. Mechanistic studies demonstrated that Pdia4 increased ROS content in β‐cells via its action on the pathway of Ndufs3 and p22^phox^. Finally, we found that 2‐β‐D‐glucopyranosyloxy1‐hydroxytrideca 5,7,9,11‐tetrayne (GHTT), a Pdia4 inhibitor, suppressed diabetic development in diabetic mice. These findings characterize Pdia4 as a crucial regulator of β‐cell pathogenesis and diabetes, suggesting Pdia4 is a novel therapeutic and diagnostic target of diabetes.

## Graphical abstract

Pancreatic β‐cell failure is associated with diabetes. Pdia4, a protein disulfide isomerase, is identified as a crucial regulator of β‐cell pathogenesis and diabetes.

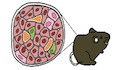

**Paper explained**

**Problem**

A gradual loss of functional pancreatic β‐cells is a cardinal feature of type 2 diabetes. Understanding the cellular and molecular mechanisms of β‐cell failure is important and may lead to improved diabetes treatment.

**Results**

Pdia4 was identified as a key protein for promoting ROS production and pathogenesis in β‐cells during diabetes in *Lepr* *^db/db^* mice and HFD‐fed B6 mice. Mechanistically, Pdia4 recruited its partners, Ndufs3 and p22^phox^, to increase ROS generation in the mitochondria and cytosol of β‐cells, leading to β‐cell failure and the development of diabetes. This recruitment involved interaction and stabilization of Ndufs3 and p22^phox^ by interaction with Pdia4. Furthermore, the Pdia4 inhibitor abolished the interaction of Pdia4 with its partners and, consequently, reduced ROS production in β‐cells and improved β‐cell failure and diabetes symptoms in *Lepr* *^db/db^* mice. Both the genetics and pharmacological approaches demonstrated that targeting Pdia4 can preserve functional β‐cells and ameliorate diabetes in mouse models.

**Impacts**

This work illustrates the novel role of the Pdia4/Ndufs3/p22^phox^ cascade as a central regulator of ROS generation in β‐cells and further establishes the new link between the Pdia4/Ndufs3/p22^phox^ cascade, which orchestrates oxidative stress, β‐cell failure and diabetes. Administration of a first‐in‐class Pdia4 inhibitor represents a feasible approach for treating β‐cell failure during diabetes. The overall findings also highlight the potential of targeting Pdia4 to prevent β‐cell loss and treat diabetes.

## Introduction

Globally, 425 million people live with diabetes, which causes about 5 million deaths annually. Diabetes is characterized by a failure of functional β‐cells to adapt insulin secretion to compensate for increasing insulin resistance, driving diabetes development (Cerf, 2013). Thus, pancreatic β‐cell failure is central to diabetes development (Matthews *et al*, 1998; Donath & Halban, 2004; Harrity *et al*, 2006). Accordingly, accumulating data suggest that preserving a portion of functional β‐cells can change the clinical outcome of diabetes (Defronzo, 2009; Leahy *et al*, 2010). However, none of the current anti‐diabetic drugs is clinically effective for this preservation. Therefore, identification of the key players in β‐cell dysfunction and death help us gain insight and understanding into β‐cell pathogenesis and diabetes development and thus aids the development of new strategies for diabetes treatment (Ardestani *et al*, 2014; Ardestani & Maedler, 2016).

The mechanism underlying the maintenance of β‐cell number and function is extremely complex and is still poorly understood. During diabetes, endoplasmic reticulum (ER) stress, inflammation, and excess nutrients can induce aberrant reactive oxygen species (ROS) in β‐cells and other cell types (Robertson *et al*, 2004; Robertson, 2004). Under normal physiological conditions, ROS are considered to be essential signaling molecules in β‐cells and other cells (Trachootham *et al*, 2009). Nevertheless, during diabetes, exuberant ROS accumulation leads to β‐cell dysfunction and death (Newsholme *et al*, 2012; Weaver *et al*, 2015) and peripheral insulin resistance (Evans *et al*, 2005) in animals and humans with diabetes. Autophagy, apoptosis, and necrosis are implicated in β‐cell death (Nakamura *et al*, 2006; Quan *et al*, 2012). The mitochondrial electron transport chain (ETC) is thought to be a major machinery for ROS production in β‐cells though NADPH oxidase (Nox), ER oxidoreductin 1 (Ero1), and certain pathways may also be implicated (Bindokas *et al*, 2003; Harrity *et al*, 2006; Leung & Leung, 2008). In contrast, ROS can be eliminated by antioxidant proteins such as glutathione peroxidase (Gpx), catalase, and superoxide dismutases (Sod). In particular, β‐cells are more vulnerable to aberrant ROS than other cell types due to the low expression level of antioxidant proteins (Lenzen *et al*, 1996; Tiedge *et al*, 1997).

Protein disulfide isomerases (Pdis) in mammals, including eight typical Pdis with CGHC motifs and 13 atypical Pdis with C/SXXC/S motifs, represent a family of multifunctional enzymes with oxidoreductase and chaperone activities (Maattanen *et al*, 2006). Most of the Pdis have an ER retention motif (Ni & Lee, 2007; Galligan & Petersen, 2012). However, more and more data show that in addition to being present in the cytosol (ER and other organelles), Pdis reside in the nuclei and membrane (Turano *et al*, 2002) and plasma of different cell types (https://www.proteinatlas.org/ENSG00000155660‐Pdia4/cell). Thus, Pdis are thought to possess ER‐relevant and ER‐irrelevant localizations and functions such as other ER chaperones (Schultz‐Norton *et al*, 2006; Xiong *et al*, 2012). This family is presumed to implement their functions via multiple mechanisms, e.g., the catalysis of disulfide bonds and conformational maintenance and regulation of their specific interaction partners and substrates (Maattanen *et al*, 2006; Schultz‐Norton *et al*, 2006). More recently, Pdia1 has been characterized as a molecular chaperone to activate estrogen receptor via stabilizing the receptor (Schultz‐Norton *et al*, 2006; Xiong *et al*, 2012). The role of Pdis in health and disease is poorly studied (Ni & Lee, 2007; Galligan & Petersen, 2012) though they might be implicated in infection (Naguleswaran *et al*, 2005; Ou & Silver, 2006), fertilization (Ellerman *et al*, 2006), coagulation (Manukyan *et al*, 2008), immunity (Garbi *et al*, 2006), tumors (Goplen *et al*, 2006), or cell viability/growth (Li & Lee, 1991; Severino *et al*, 2007). Emerging evidence obtained from yeast and worms suggests that the function of Pdis is not always redundant (Norgaard *et al*, 2001; Winter *et al*, 2007). Pdia4 is structurally unique because it is the largest member with three GCHC motifs in the family. Unlike *Pdia3* (Garbi *et al*, 2006), *Pdia4* is not an essential gene since its knockout mice were shown to survive without any noticeable phenotype (Almeida *et al*, 2011; Kuo *et al*, 2017). Its expression could be further induced by calcium flux (Li & Lee, 1991), ER stress (Li & Lee, 1991; Parker *et al*, 2001) and hypoxia (Pawar *et al*, 2011) in tumors. However, like other Pdis, nothing is known about the role of Pdia4 in diabetes, and its therapeutic potential and molecular basis in diabetes has not been deciphered.

In this study, we first evaluated the expression level of Pdia4 in the pancreatic islets and sera in mice and humans. Next, mice with Pdia4 knockout and overexpression were generated to evaluate the impact of Pdia4 on β‐cell pathogenesis and diabetes. In parallel, we elucidated the molecular mechanism of Pdia4 in ROS generation and β‐cell pathogenesis. Finally, we identified 2‐β‐D‐glucopyranosyloxy1‐hydroxytrideca 5,7,9,11‐tetrayne (GHTT) as a Pdia4 inhibitor, which was used to assess its anti‐diabetic potential in diabetic mice.

## Results

### Up‐regulation of Pdia4 protein in pancreatic islets and sera of mouse and human origin in response to excess nutrients

**Figure 1:**
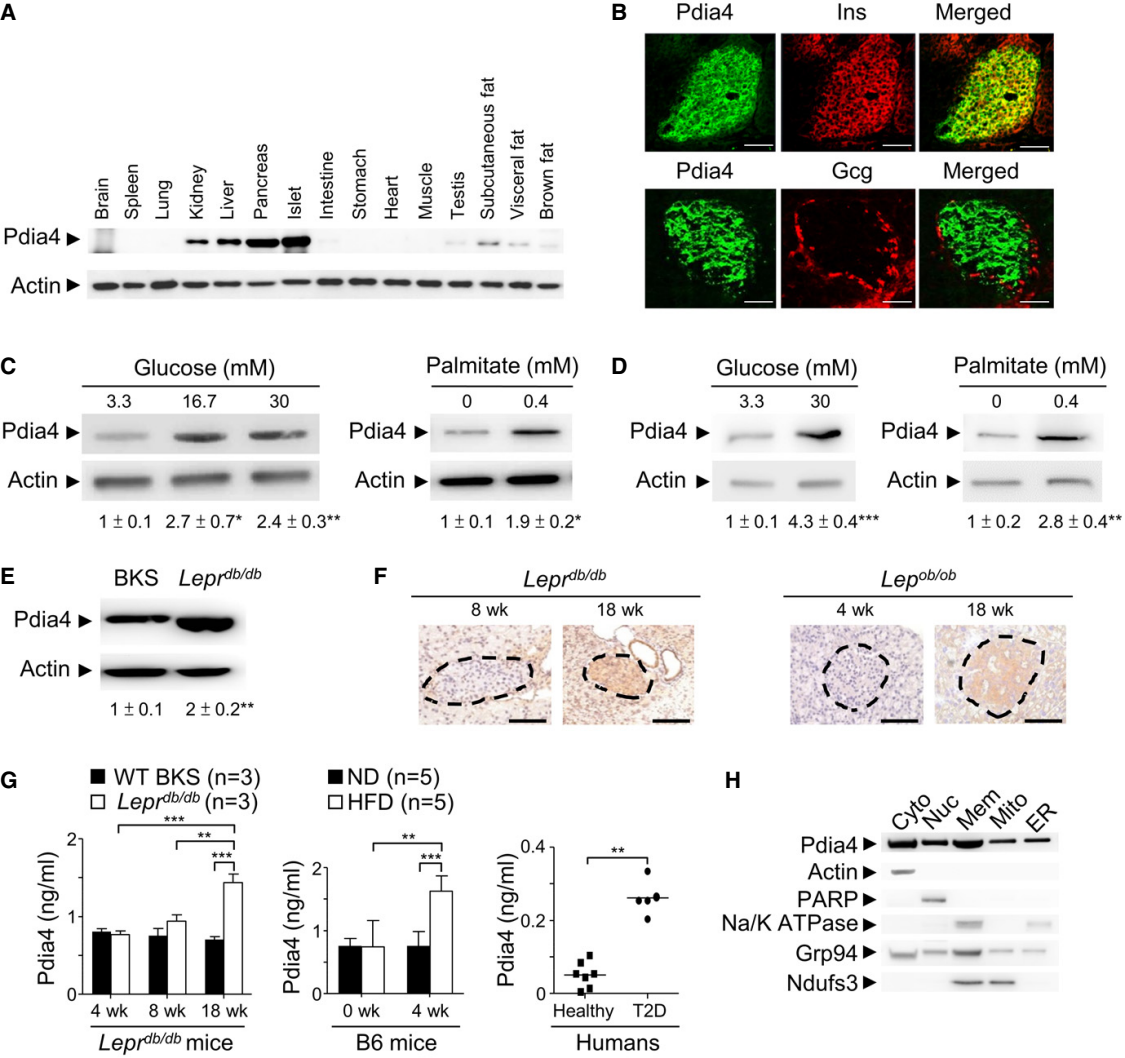
Expression and distribution of Pdia4 in pancreatic islets and/or other tissues ATotal lysates of different mouse organs underwent immunoblotting analysis using anti‐Pdia4 and anti‐actin antibodies.BConfocal analysis of Pdia4 and insulin (Ins) or glucagon (Gcg) in mouse islets. The tissues were stained with the indicated antibodies and analyzed using a confocal microscope. Scale bar = 100 μm.CMin6 cells were treated with glucose and palmitate at the indicated dosages. Total lysate underwent immunoblotting analysis using the indicated antibodies.DHuman islets were treated with glucose and palmitate at the indicated dosages, followed by immunoblotting analysis.EThe islets of WT BKS and diabetic *Lepr* ^db/db^ mice underwent immunoblotting analysis.FIHC analysis of the islets from *Lepr* ^db/db^ and *Lep* ^ob/ob^ mice at the indicated ages using anti‐Pdia4 antibody and hematoxylin. Scale bar = 100 μm. The dash circles indicate islet regions.GSera from wild‐type (WT) BKS and *Lepr* ^db/db^ mice, aged 4, 8, and 18 weeks, B6 mice, fed with a normal diet (ND) and a high‐fat diet (HFD) for 4 weeks, and humans, healthy volunteers and diabetic patients (T2D), were quantified for Pdia4 level using an anti‐Pdia4 ELISA kit.HImmunoblotting analysis of Pdia4 and markers in the cytosolic (Cyto), nuclear (Nuc), membrane (Mem), mitochondrial (Mito), and ER compartments of Ming 6 cells using the indicated antibodies. Data information: Data from 3 experiments (A–E, F and H) and more (G) are presented as the mean ± SD. One‐way ANOVA test was used for statistical analysis of differences between groups, and *P* (*) < 0.05; *P* (**) < 0.01 and *P* (***) < 0.001 are considered statistically significant. The number of mice (*n*) is indicated in parentheses. Source data are available online for this figure.

To explore the likely role of Pdis in β‐cells, we first compared the mRNA level of 8 typical Pdis (a1, a2, a3, a4, a5, a6, a13, and a15) and 2 atypical Pdis (a16 and a19) in Min6 cells, a mouse β‐cell line, and mouse islets. The real‐time polymerase chain reaction (RT–PCR) data showed that 4 Pdis (a1, a3, a4, and a6) had a higher transcriptional level than the rest in those cells (Appendix Fig S1A). In marked contrast, the protein level of Pdia4, but not Pdia1, Pdia3, or Pdia6, was up‐regulated by glucose in Min6 cells (Appendix Fig S1B). This up‐regulation was consistent with the presence of a putative ER stress responsive element (ERSE) in the *Pdia4* promoter (Appendix Fig S1C). Further, high glucose increased the activity of Pdia4 promoter in Min6 cells (Appendix Fig S1D). Next, we investigated the expression pattern of Pdia4 in mouse tissues. We discovered that Pdia4 was expressed in mouse pancreata and islets to a greater extent than in liver, kidney, testis, and fat tissue (Fig 1A). Further, Pdia4 was expressed in β‐cells but not α‐cells of mouse islets (Fig 1B). However, we could not rule out its expression in other pancreatic cell types. Of note, this expression was up‐regulated in response to a high dose of glucose (left, Fig 1C) and palmitate (right, Fig 1C) in Min6 cells. Likewise, Pdia4 was expressed in human islets and this expression was further up‐regulated by excess nutrients (Fig 1D). Accordingly, the *in vivo* studies revealed that Pdia4 was expressed in pancreatic islets of wild‐type (WT) control mice and this expression level was further elevated in pancreatic islets of diabetic *Lepr^db/db^* mice (Fig 1E). The up‐regulation of Pdia4 in pancreatic islets correlated well with diabetes development in *Lepr^db/db^* mice and *Lep^ob/ob^* mice (Fig 1F), two spontaneous mouse models of diabetes. Equally importantly, serum Pdia4 also went up with diabetes development in *Lepr^db/db^* mice, high‐fat diet (HFD)‐fed B6 mice, and diabetic patients (Fig 1G). Since Pdia4 was initially documented as an ER‐resident protein with an ER retention motif, KEEL^642–645^, at its C‐terminus (Ni & Lee, 2007; Galligan & Petersen, 2012), we thus examined the subcellular distribution of Pdia4 in Min6 β‐cells. Surprisingly, the immunoblotting data indicated that Pdia4 was distributed in the nuclei, cytosol, membrane, mitochondria, and ER of Min6 cells (Fig 1H). Consistently, mass spectroscopy (MS) data also confirmed that despite its KEEL motif, Pdia4 resided in the aforesaid compartments of Min6 β‐cells and mouse serum (Appendix Fig S1E).

Overall, the data showed that Pdia4 was expressed in the pancreas and was distributed in different cell compartments. The remainder of the study concentrated on the investigation of Pdia4 in β‐cell pathogenesis and diabetes.

### Reduction of ROS, HbA1c, islet atrophy, and islet cell death, and increase in β‐cell function and longevity in Pdia4‐deficient mice

**Figure 2:**
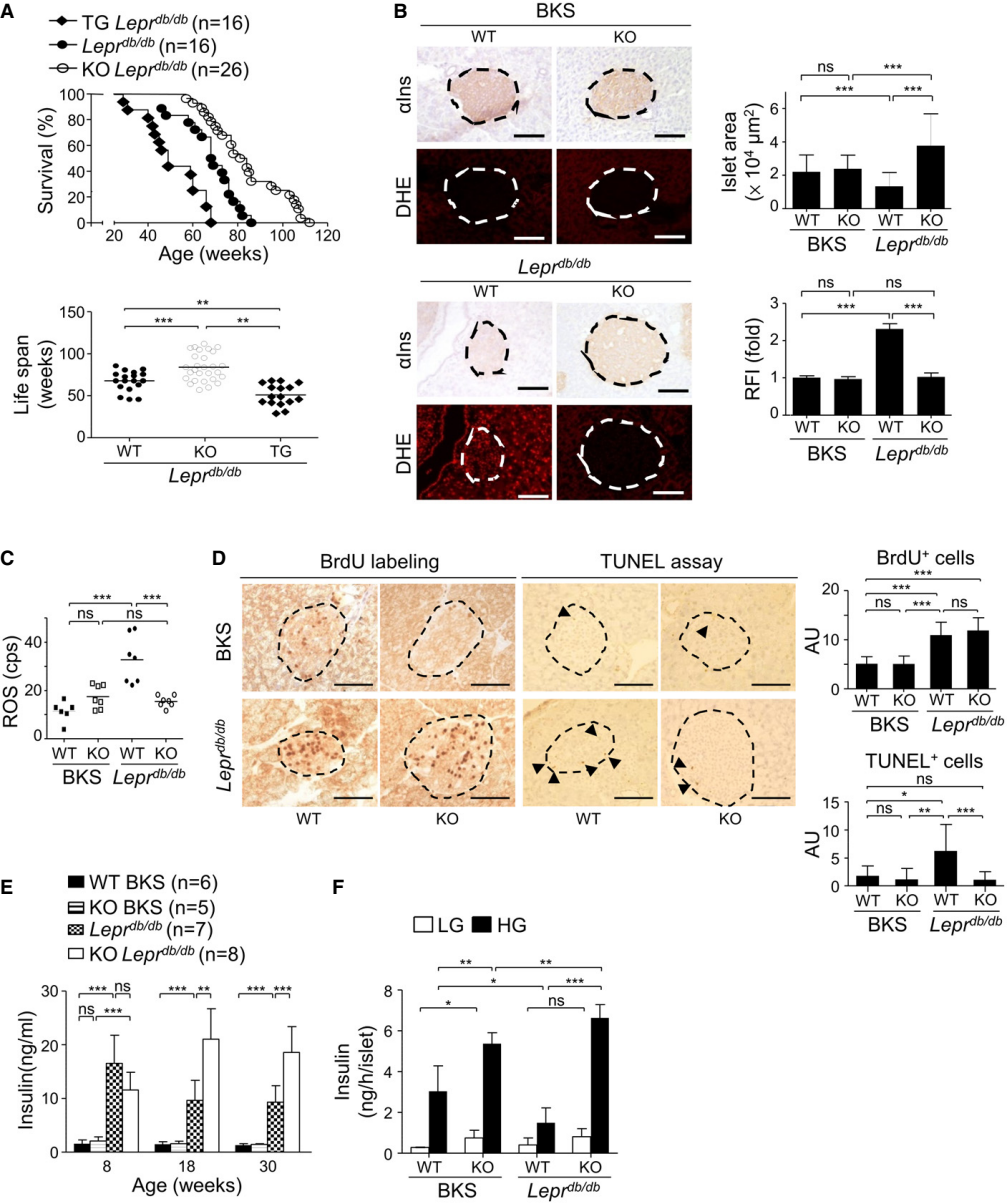
Pdia4 ablation increases survival rate and longevity and reduces islet atrophy, ROS production, and islet cell death, elevates serum insulin, and potentiates β‐cell GSIS in *Pdia4* ^−/−^ *Lepr^db/db^* mice ASurvival rate and life span of WT, *Pdia4^−/−^* (KO), and Pdia4^tg/tg^ (TG) mice on *Lepr^db/db^* background from birth to 120 weeks (Appendix Figs S2A and S6A).BPancreata of 18‐week‐old WT and *Pdia4^−/−^* (KO) mice on BKS or *Lepr^db/db^* background were stained with anti‐insulin (αIns) antibody and dihydroethidium (DHE) (left). Islet area (μm^2^) and relative fluorescence intensity (RFI) were quantified (right). Scale bar: 100 μm. The dash circles indicate islet regions.CSerum ROS of the 18‐week‐old mice (B) were determined.DThe same batch mice as in (B) were given water containing BrdU. The BrdU^+^ cells of the islets were visualized and quantified (BrdU^+^ labeling). TUNEL‐positive cells in the islets of the mice (B) were visualized and quantified (TUNEL assay). BrdU^+^ cells per islet area (0.01 mm^2^) and TUNEL^+^ cells per islet area (0.05 mm^2^) are expressed in arbitrary units (AU). The dash circles indicate islet regions, and the black arrowheads indicate TUNEL^+^ cells.E, FELISA kits were used to quantify serum insulin (E) of the same mice as in (B) and their supernatants of the mouse islets (F) in GSIS assays. High glucose (HG, 16.7 mM) or low glucose (LG, 3.3) was used in GSIS assays. Data information: The number of mice (*n*) is indicated in parentheses. Data from over three experiments are presented as the mean ± SD. Log rank (A) and one‐way ANOVA test (B–F) were used for statistical analysis of differences between groups, and *P* (*) < 0.05; *P* (**) < 0.01 and *P* (***) < 0.001 are considered statistically significant. Source data are available online for this figure.

The fact that the Pdia4 expression increased with diabetes development in mice and humans prompted us to examine whether it could trigger β‐cell pathogenesis and diabetes. First, conventional Pdia4 knockout mice (*Pdia4^−/−^* B6) were bred as published (Almeida *et al*, 2011; Kuo *et al*, 2017). The mice were bred into BKS (*Pdia4^−/−^* BKS) and, subsequently, *Lepr^db/db^* backgrounds (*Pdia4^−/−^ Lepr^db/db^*) in Appendix Fig S2A. WT and *Pdia4^−/−^* mice on B6 (Almeida *et al*, 2011; Kuo *et al*, 2017) and BKS backgrounds (Appendix Fig S2) were diabetes‐free. As expected, *Lepr^db/db^* mice spontaneously developed diabetes by 8 weeks of age and this diabetes became more severe with age as evidenced by fasting blood glucose (FBG, Appendix Fig S2A), postprandial blood glucose (PBG, Appendix Fig S2A), the percentage of glycosylated hemoglobin A1c (Hb_A1c_, Appendix Fig S2B), glucose tolerance (Appendix Fig S2C), homeostatic model assessment (HOMA) indices (Appendix Fig S2D), and diabetic incidence (Appendix Fig S2E). In sharp contrast, *Pdia4^−/−^Lepr^db/db^* mice developed borderline diabetes with average FBG and PBG of around 109 and 289 mg/dl at 24 weeks of age, respectively (Appendix Fig S2A). Closer analysis revealed that 58% of the *Pdia4^−/−^Lepr^db/db^* mice whose PBG was 137 mg/dl by 24 weeks of age, were diabetes‐free (*Pdia4^−/−^Lepr^db/db^*, Appendix Fig S2E) compared to the rest (42%) which exhibited slight diabetes with PBG around 340 mg/dl (*Pdia4^−/−^Lepr^db/db^*, Appendix Fig S2E). The diabetes‐free and diabetic mice had daily food intake of 6.8 and 7.9 g, respectively. Regression analysis indicated a strong correlation between PBG and food intake in *Pdia4^−/−^Lepr^db/db^* mice (*R* ^2^ = 0.98, Appendix Fig S2F). Consistently, *Pdia4^−/−^Lepr^db/db^* mice, given 6.8 g feed/day/mouse from 4 to 24 weeks of age, showed completely arrested diabetes development (*Pdia4^−/−^Lepr^db/db^* + FR, Appendix Fig S2E). In contrast, a ROS scavenger, vitamin C, at 375 mg/kg failed to affect diabetes development in *Lepr^db/db^* mice (*Lepr^db/db^* + VitC, Appendix Fig S2E). As published (Brem *et al*, 2007), *Lepr^db/db^* mice showed decreased food intake and PBG with age over a period of 90 weeks (Appendix Fig S3A and B). However, *Pdia4^−/−^Lepr^db/db^* mice, aged 55 weeks and over, had much lower PBG and HbA1c than the age‐matched *Lepr^db/db^* mice (Appendix Fig S3B and C). Of note, *Pdia4^−/−^Lepr^db/db^* mice showed more effective amelioration of diabetes than the age‐matched *Lepr^db/db^* mice as shown by water consumption (right, Appendix Fig S3A), PBG (Appendix Fig S3B), glucose tolerance (Appendix Fig S3D), islet preservation (Appendix Fig S3E), survival rate (top, Fig 2A), and life span (bottom, Fig 2A). No significant difference in food intake was observed between *Lepr^db/db^* and *Pdia4^−/−^Lepr^db/db^* mice, from 8 to 90 weeks (left, Appendix Fig S3A). Strikingly, all the *Pdia4^−/−^Lepr^db/db^* mice became diabetes‐free by the age of 55 weeks and beyond, which correlated with decreased food intake in aged mice (Appendix Fig S3).

We also examined the structure and function of pancreatic islets in WT and *Pdia4^−/−^* mice. Islet size in the four mouse lines aged 18 weeks was in the following ascending order: *Lepr^db/db^* mice < WT BKS mice = *Pdia4^−/−^* BKS mice < *Pdia4^−/−^Lepr^db/db^* mice (αIns, Fig 2B). Also of note, the degree of islet atrophy in both mouse backgrounds correlated with ROS accumulation in islets (dihydroethidium (DHE), Fig 2B) and blood (Fig 2C). To investigate the role of Pdia4 in β‐cell proliferation and demise, we checked cell proliferation and death of mouse islets using 5‐bromo‐2‐deoxyuridine (BrdU) labeling assays and terminal deoxynucleotidyl transferase dUTP nick end labeling (TUNEL), respectively. BrdU data showed no significant difference in cell proliferation of pancreatic islets between WT and *Pdia4^−/−^* mice on BKS and *Lepr^db/db^* backgrounds irrespective of Pdia4 content (left, Fig 2D). In sharp contrast, *Lepr^db/db^* mice had more TUNEL‐positive islet cells, dead cells, and living cells undergoing proliferation and repair, than *Pdia4^−/−^Lepr^db/db^* mice, WT, and *Pdia4^−/−^* BKS mice (right, Fig 2D). Both types of assays suggest that Pdia4 is inversely correlated to cell death in mouse islets. Furthermore, we generated islet‐specific Pdia4 knockout mice (*Pdia4^f/f^Lepr^db/db^Cre^tg/0^*) to verify the islet‐specific function of Pdia4 in islets and diabetes (Appendix Fig S4). The conditional Pdia4 knockout mice (Appendix Fig S4) had similar pre‐clinical parameters to the conventional Pdia4 knockout mice (Fig 2 and Appendix Fig S2) in terms of FBG, PBG, Hb_A1c_, serum insulin, islet atrophy, ROS, HOMA indices, and glucose tolerance.

Moreover, *Pdia4^−/−^* mice had better β‐cell function than WT mice on BKS and *Lepr^db/db^* backgrounds as evidenced by HOMA‐β (Appendix Fig S2D), serum insulin (Fig 2E), and glucose‐stimulated insulin secretion (GSIS) (Fig 2F). At 8 weeks of age, *Lepr^db/db^* and *Pdia4^−/−^Lepr^db/db^* mice had a high but similar level of serum insulin compared to a low serum insulin level in WT BKS and *Pdia4^−/−^* BKS mice (8 weeks, Fig 2E). However, at 18 weeks of age and beyond, *Pdia4^−/−^Lepr^db/db^* mice continued to produce high serum insulin while *Lepr^db/db^* mice gradually lost their serum insulin (18 and 30 weeks, Fig 2E). Consistently, GSIS data indicated that *Pdia4^−/−^* islets had better function than WT islets regardless of mouse background (Fig 2F). Serum insulin change seemed to reflect the status of β‐cell function and/or insulin resistance.

Taken together, the loss‐of‐function data suggest that Pdia4 is a key regulator for β‐cell pathogenesis and diabetes.

### Increase in diabetes, ROS, HbA1c, and islet atrophy and decrease in β‐cell function in Pdia4 transgenic mice

**Figure 3:**
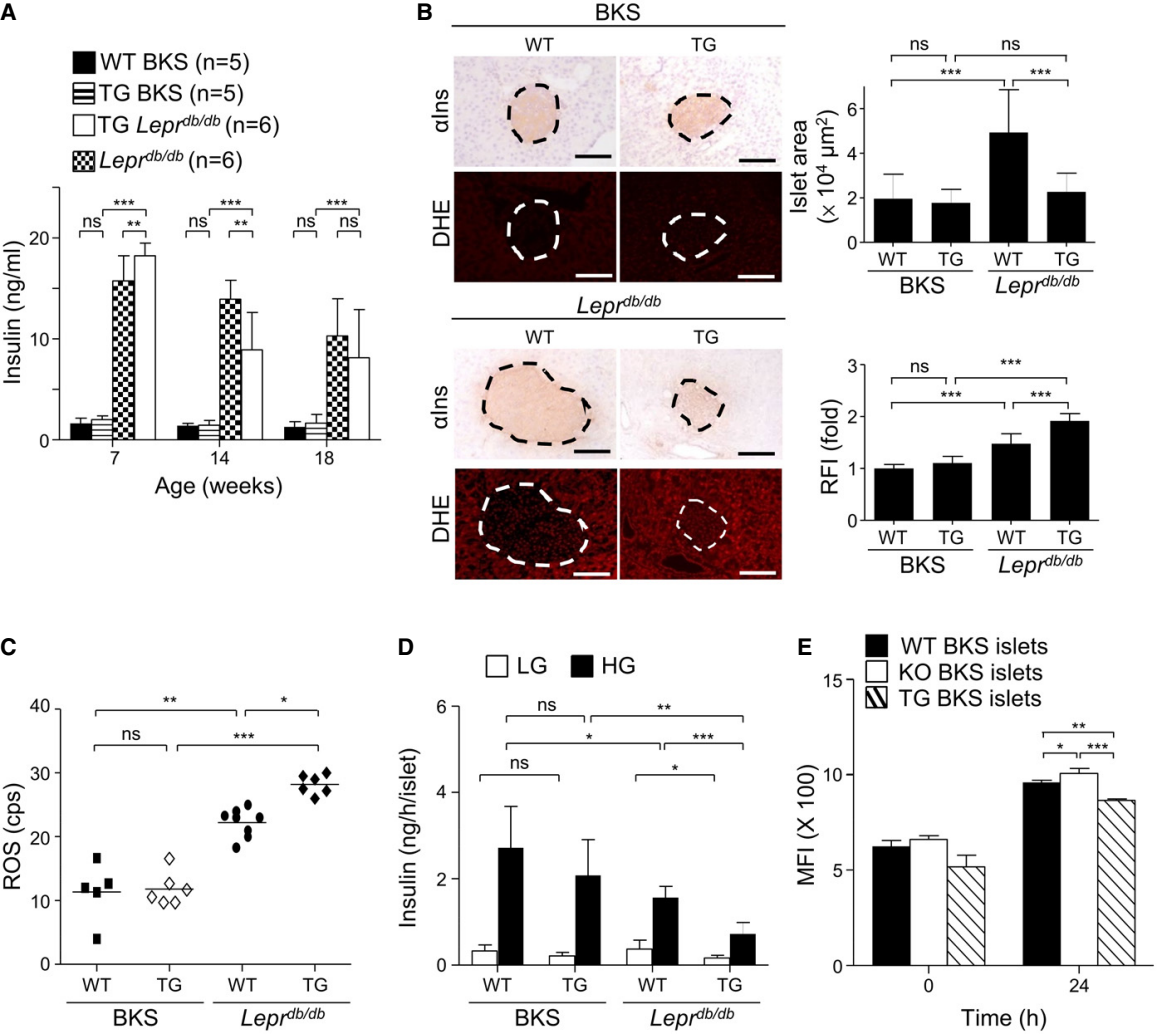
Pdia4 overexpression increases islet atrophy and ROS production, decreases serum insulin, and impairs β‐cell GSIS in Pdia4^tg/tg^ *Lepr^db/db^* mice ASerum insulin of WT and Pdia4^tg/tg^ (TG) mice on BKS or *Lepr^db/db^* background (Appendix Fig S6A), at the indicated ages, was measured using an ELISA kit.BPancreata of the same batch of mice as in (A), at the age of 14 weeks, were stained with anti‐insulin (αIns) antibody and DHE (left). Islet area (μm^2^) and relative fluorescence intensity (RFI) were quantified (right). Scale bar = 100 μm. The dash circles indicate islet regions.CSerum ROS of the mice from (A) were determined.DThe islets of the mice from (C) were tested for GSIS. High glucose (HG, 16.7 mM) or low glucose (LG, 3.3 mM) was used in GSIS assays.EPancreatic islets of WT, *Pdia4^−/−^* (KO) and Pdia4^tg/tg^ (TG) BKS mice, which received an intraperitoneal dose of glucose (1 g/kg body weight) for 0.5 h, were isolated and divided into 2 aliquots. One aliquot of the islets was stained with anti‐insulin antibody and measured for their basal insulin content using flow cytometry analysis (0 h). The other aliquot of the islets were grown in a complete medium for 24 h, and their insulin content was measured (24 h). Data information: Data from 3 experiments (E) and more (A–D) are presented as the mean ± SD. One‐way ANOVA test was used for statistical analysis of differences between groups, and *P* (*) < 0.05; *P* (**) < 0.01 and *P* (***) < 0.001 are considered statistically significant. The number of mice (*n*) is indicated in parentheses. Source data are available online for this figure.

In parallel, a gain‐of‐function approach was taken to confirm the role of Pdia4 in diabetes development. Pdia4 transgenic (Pdia4^tg/tg^) mice on B6, BKS, and *Lepr^db/db^* backgrounds, which expressed a high level of transgenic Pdia4 in islets, were created and monitored for diabetes (Appendix Fig S5A). No noticeable phenotypes were observed in WT and transgenic mice on B6 and BKS backgrounds (Appendix Figs S5 and S6). HFD‐fed B6 mice were used as a murine model because they are closest to human metabolic syndrome (Hinder *et al*, 2017). However, HFD‐fed Pdia4^tg/tg^ B6 mice developed moderate diabetes as evidenced by BG, HbA_1c_, GTT, and diabetic incidence in comparison with HFD‐fed WT and *Pdia4^−/−^* B6 mice (Appendix Fig S5A–E). Further, HFD‐fed Pdia4^tg/tg^ B6 mice had worse HOMA‐β and islet atrophy than HFD‐fed WT and *Pdia4^−/−^* B6 mice (Appendix Fig S5F and G). Likewise, Pdia4^tg/tg^ *Lepr^db/db^* mice spontaneously developed diabetes by 6 weeks of age, around 2 weeks earlier than the age‐matched *Lepr^db/db^* controls, and, finally, both lines reached a profound degree of diabetes as evidenced by FBG, PBG and HbA_1c_ (Appendix Fig S6A). At 7 weeks of age, Pdia4^tg/tg^ *Lepr^db/db^* mice had a higher level of serum insulin than *Lepr^db/db^* mice (Fig 3A). Afterward, the serum insulin in Pdia4^tg/tg^ *Lepr^db/db^* and *Lepr^db/db^* gradually decreased with age (14–18 weeks, Fig 3A). However, WT and Pdia4^tg/tg^ BKS mice had basal serum insulin levels that remained unaltered over the lifetime (Fig 3A). The change in serum insulin was consistent with HOMA indices (Appendix Fig S6B) and glucose tolerance (Appendix Fig S6C) in the four mouse lines. Further, we checked the structure and function of pancreatic islets in WT and Pdia4^tg/tg^ mice on BKS and *Lepr^db/db^* backgrounds. By 14 weeks of age, islet size in the four mouse lines was (in descending order), *Lepr^db/db^* mice > Pdia4^tg/tg^ *Lepr^db/db^* mice = Pdia4^tg/tg^ BKS mice = WT BKS mice (αIns, Fig 3B). Similar to islet atrophy (αIns, Fig 3B), ROS accumulation in the islets (DHE, Fig 3B), and sera (Fig 3C) of Pdia4^tg/tg^ *Lepr^db/db^* mice was more pronounced than that seen in the islets and sera of *Lepr^db/db^*, Pdia4^tg/tg^ BKS mice, and WT BKS mice. Moreover, *Pdia44^tg/tg^Lepr^db/db^* mice had worse β‐cell function than *Lepr^db/db^* mice, Pdia4^tg/tg^ BKS, and WT BKS mice as evidenced by *in vitro* GSIS assays (Fig 3D) and *in vivo* HOMA‐β (right, Appendix Fig S6B). Accordingly, flow cytometry analysis showed that the basal level of insulin content in *Pdia4^−/−^* islets was slightly higher than that of insulin content in WT and Pdia4^tg/tg^ islets (0 h, Fig 3E). Likewise, *Pdia4^−/−^* islets produced slightly more insulin than WT and Pdia4^tg/tg^ islets in complete DMEM medium 24 h post‐culture (24 h, Fig 3E). Collectively, the data on Pdia4 overexpression suggest that Pdia4 promotes β‐cell pathogenesis and diabetes.

### Pdia4 in the regulation of cell dysfunction, cell death, ROS production, and the activity of Nox and ETC CI in pancreatic islets

**Figure 4:**
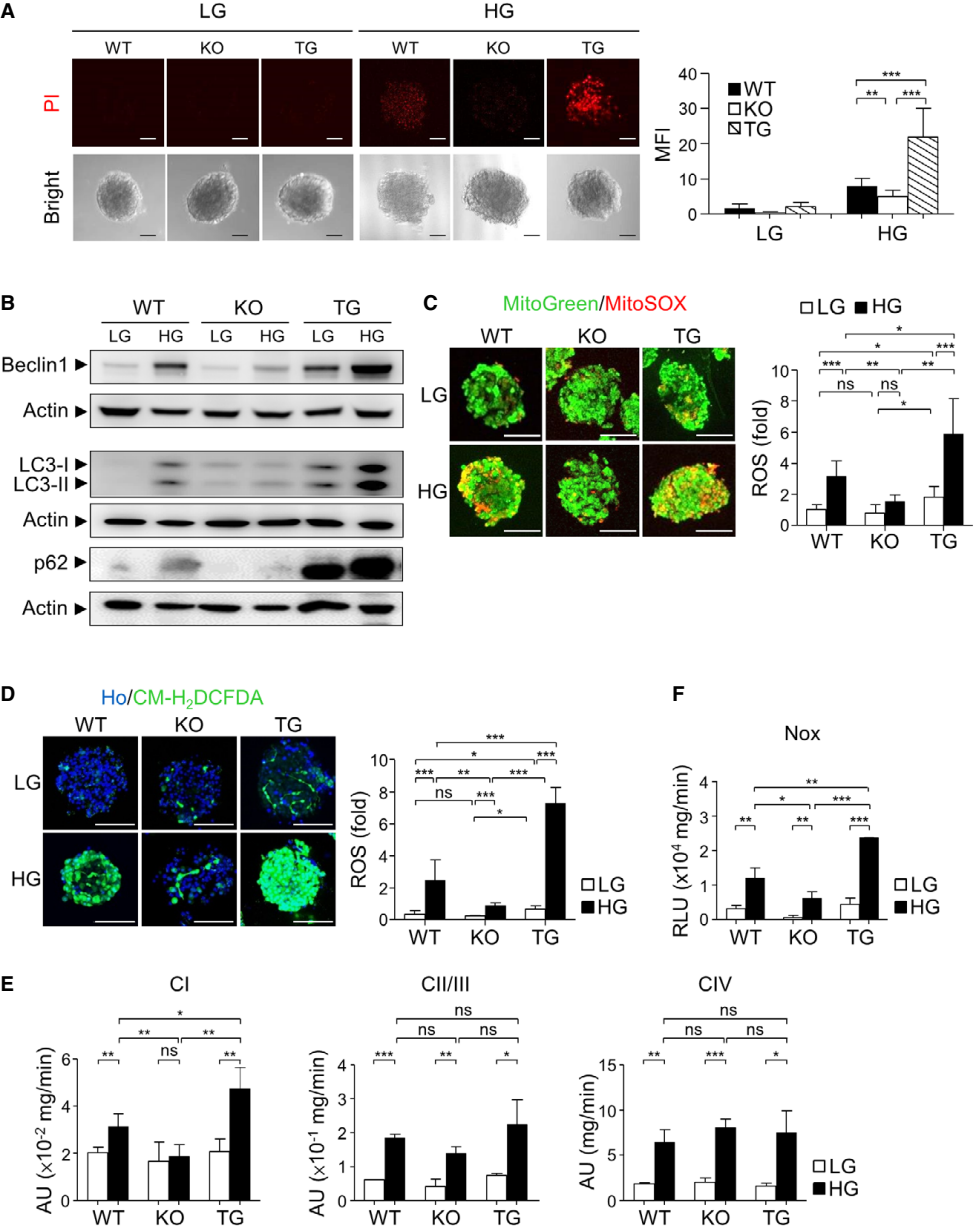
Pdia4 is involved in the regulation of cell death, ROS accumulation, and the activity of ETC and NOX AThe islets of WT, *Pdia4^−/−^* (KO), and Pdia4^tg/tg^ (TG) BKS mice were isolated and grown in complete DMEM medium containing 3.3 mM (LG) and 30 mM glucose (HG) for 12 h. The islets stained with propidium iodide (PI), photographed, and quantified. Scale bar = 50 μm.BThe islets from the mice (A) were treated with 3.3 mM (LG) or 30 mM glucose (HG). After lysis, the lysates underwent immunoblotting analysis using the antibody against Beclin 1, LC3, p62 and actin.C, DThe islets from the mice (A) were incubated with MitoGreen plus MitoSOX (C) or Hoechst 33342 (Ho) plus CM‐H_2_DCFDA (D) in the presence of glucose at 3.3 mM (LG) and 16.7 mM (HG). Mitochondrial (C) and cytosolic ROS (D) in the islets were visualized and quantified. Scale bar = 100 μm.EMitochondria isolated from the mouse islets (A) were measured for the activity of ETC CI (left), CII/III (middle), and CIV (right) using MitoCheck assays.FThe membrane fraction of the pancreas from the mice (A) was measured for Nox activity using luminometric assays. Data information: Data from three experiments are presented as the mean ± SD. One‐way ANOVA test was used for statistical analysis of differences between groups, and *P* (*) < 0.05; *P* (**) < 0.01 and *P* (***) < 0.001 are considered statistically significant. Source data are available online for this figure.

Given that Pdia4 was implicated in the increase of ROS generation and islet atrophy during diabetes, we next investigated cell death of pancreatic islets in WT, *Pdia4^−/−^*, and Pdia4^tg/tg^ BKS mice. Propidium iodide (PI) staining data indicated a similar basal extent of cell death in Pdia4^tg/tg^, WT, and *Pdia4^−/−^* islets in the presence of low glucose (LG, Fig 4A). In contrast, Pdia4^tg/tg^ islets had more cell death than WT and *Pdia4^−/−^* islets in the presence of high glucose (HG, Fig 4A). Immunoblotting data showed that Pdia4^tg/tg^ islets had slightly more Beclin 1, LC3‐I, LC3‐II, and p62 than the WT and *Pdia4^−/−^* islets in the presence of low glucose (LG, Fig 4B). In contrast, Beclin 1, LC3, and p62 showed a greater increase in Pdia4^tg/tg^ islets in comparison with WT and *Pdia4^−/−^* islets in response to high glucose (HG, Fig 4B). Next, we compared the ROS content in the mitochondria and cytosol of pancreatic islets of WT, *Pdia4^−/−^*, and Pdia4^tg/tg^ BKS mice. The mitochondrial ROS level in pancreatic islets of BKS mice at a low glucose concentration was (in descending order): Pdia4^tg/tg^ islets > WT islets = *Pdia4^−/−^* islets (LG, Fig 4C). In contrast, the mitochondrial ROS level of mouse pancreatic islets at a high glucose concentration was (in descending order): Pdia4^tg/tg^ islets > WT islets > *Pdia4^−/−^* islets (HG, Fig 4C). Similarly, the cytosolic ROS level in the pancreatic islets of BKS mice at a low glucose concentration was (in descending order): Pdia4^tg/tg^ islets > WT islets = *Pdia4^−/−^* islets (LG, Fig 4D). In contrast, the cytosolic ROS level in mouse pancreatic islets at a high glucose concentration was (in descending order): Pdia4^tg/tg^ islets > WT islets > *Pdia4^−/−^* islets (HG, Fig 4D).

Furthermore, we measured mitochondrial ETC and Nox activities in pancreatic islets in WT, *Pdia4^−/−^*, and Pdia4^tg/tg^ BKS mice using MitoCheck assays and luminometry, respectively. MitoCheck assays indicated that the activity of ETC CI in the isolated mitochondria of the three mouse pancreatic islets was (in descending order): Pdia4^tg/tg^ islets > WT islets > *Pdia4^−/−^* islets (CI, Fig 4E). However, there was no difference in the activity of ETC CII/III and ETC CIV (CII/III and CIV, Fig 4E) in the isolated mitochondria of three mouse pancreatic islets. On the contrary, luminometric assays demonstrated that the Nox activity in pancreatic islets of BKS mice at a low glucose concentration was (in descending order): Pdia4^tg/tg^ islets > WT islets = *Pdia4^−/−^* islets (LG, Fig 4F). However, the Nox activity in pancreatic islets of BKS mice at a high glucose concentration was (in descending order): Pdia4^tg/tg^ islets > WT islets > *Pdia4^−/−^* islets (HG, Fig 4F). In parallel, we assessed the effect of Pdia4 on the expression level of antioxidant enzymes in mouse pancreatic islets. We found no difference in the protein levels of Sod1, Sod2, Gpx1, and catalase in the islets of WT, *Pdia4^−/−^*, and Pdia4^tg/tg^ mice (Appendix Fig S6E).

**Figure 5:**
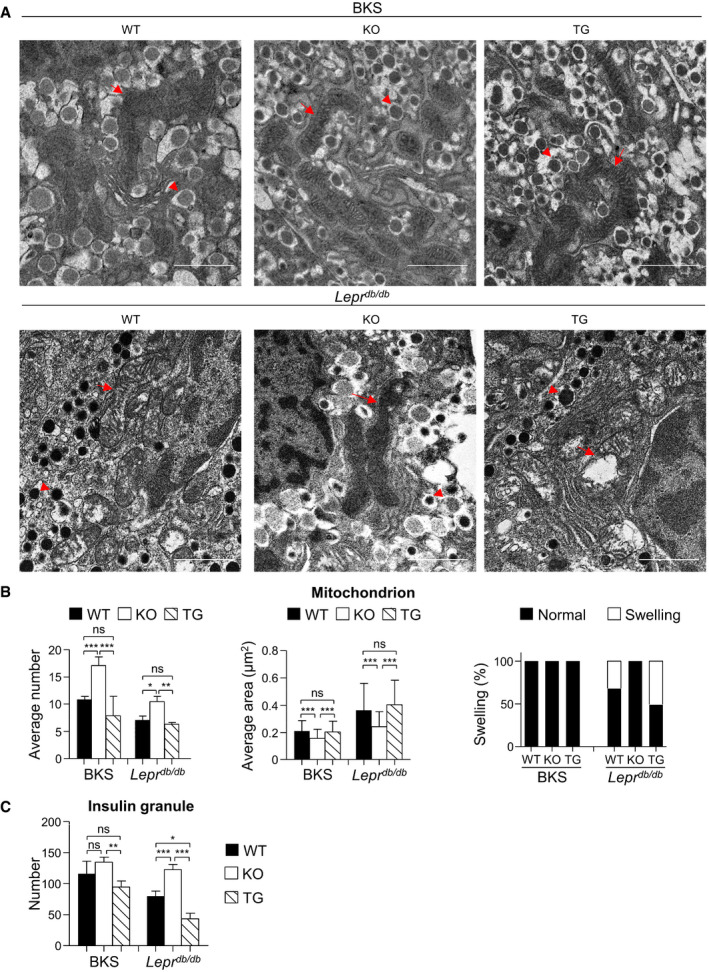
Pdia4 affects mitochondria and insulin granules in β‐cells AUltrastructure of mitochondria and insulin granules in typical β‐cells of three mouse lines on BKS and *Lepr^db/db^* backgrounds. Arrows indicate the mitochondria whereas arrowheads indicate insulin granules. Scale bar = 1 μm.BAverage number (left), average area (middle), and swelling percentage (right) of the β‐cell mitochondria from the mouse pancreatic islets (A) in microscopic sections.CThe average number of insulin granules in β‐cells from the mouse pancreatic islets (A) in microscopic sections. Data information: A total of 15–23 images per group were analyzed. Data from each group are presented as the mean ± SD. One‐way ANOVA test was used for statistical analysis of differences between groups, and *P* (*) < 0.05; *P* (**) < 0.01; and *P* (***) < 0.001 are considered statistically significant. Source data are available online for this figure.

We also checked the significance of Pdia4 in the function of β‐cells using a transmission electron microscope (Fig 5A). We found that the number of mitochondria in β‐cells of BKS and *Lepr^db/db^* mice was (in descending order): *Pdia4^−/−^* β‐cells > WT β‐cells > Pdia4^tg/tg^ β‐cells (left, Fig 5B). In contrast, the average area and swelling of β‐cell mitochondria in BKS and *Lepr^db/db^* mice was (in descending order): Pdia4^tg/tg^ β‐cells > WT β‐cells > *Pdia4^−/−^* β‐cells (middle and right, Fig 5B). In addition, the insulin granule number of β‐cells in BKS and *Lepr^db/db^* mice was (in descending order): *Pdia4^−/−^* β‐cells > WT β‐cells > Pdia4^tg/tg^ β‐cells (Fig 5C). The data on mitochondria and insulin granules imply an inverse association between Pdia4 and β‐cell function.

Overall, the data suggest that Pdia4 modulates ROS production and, subsequently, cell death and cell dysfunction in β‐cells through the regulation of Nox and ETC activities.

### Characterization of the interaction of Pdia4 with Ndufs3 and p22^phox^

**Figure 6:**
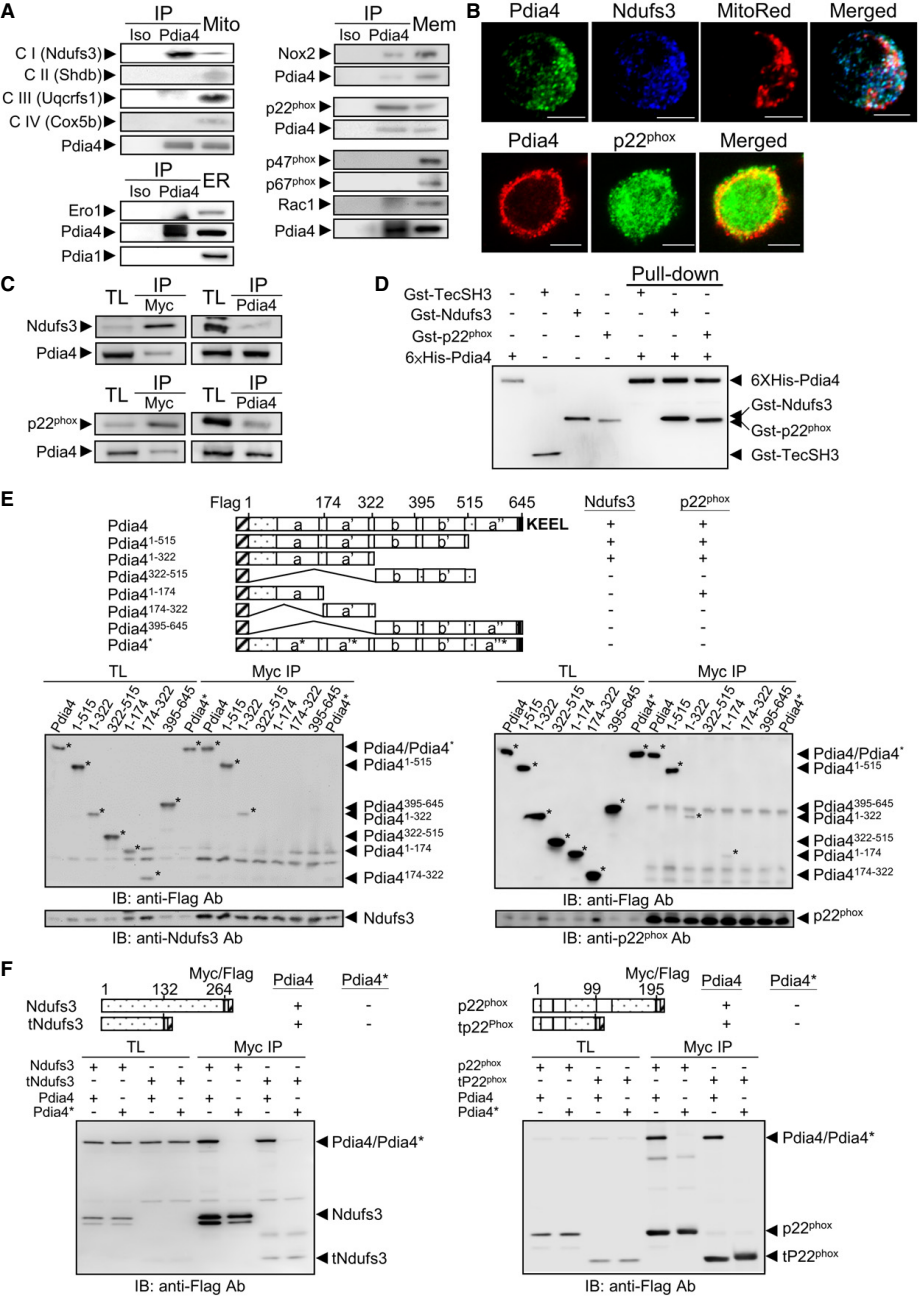
Binding domain mapping analysis of Pdia4 and Ndufs3 or p22^phox^ AMitochondrial (Mito, top left), ER (bottom left) and membrane (Mem, right) fractions of Min6 cells were precipitated with isotype (Iso) or anti‐Pdia4 (Pdia4) antibody. The fractions and immunoprecipitates (IP) underwent immunoblotting analysis.BConfocal analysis of the indicated proteins in Min6 cells. Min6 cells were intracellularly stained with anti‐Pdia4 and anti‐Ndufs3 antibodies plus MitoRed (top). Min6 cells were surface stained with anti‐Pdia4 antibody, followed by intracellular staining with anti‐p22^phox^ antibody (bottom). Scale bar = 5 μm.CThe construct encoding Flag‐Pdia4 and that expressing Myc/Flag‐tagged Ndufs3 (top) or p22^phox^ (bottom) were co‐transfected into 293T cells. Total lysates of the cells were incubated with anti‐Myc (left) or anti‐Pdia4 (right) antibodies plus protein G beads. Their total lysates (TL) and immunoprecipitates (IP) underwent immunoblotting analysis anti‐Flag antibody.DHis‐tagged Pdia4 was incubated with the recombinant proteins, Gst‐TecSH3, Gst‐Ndufs3, and Gst‐p22^phox^. The immunoprecipitates (IP) and recombinant proteins underwent immunoblotting analysis with anti‐Gst and anti‐His antibodies.EThe construct encoding Flag‐Pdia4 or its mutants and that expressing Myc/Flag‐tagged Ndufs3 (left) or p22^phox^ (right) were co‐transfected into 293T cells. Total lysates (TL) and anti‐Myc immunoprecipitates (Myc IP) underwent immunoblotting analysis. N‐terminal Flag tag, catalytic domains (a, a', and a''), non‐catalytic domains (b and b'), and C‐terminal KEEL are indicated. Pdia4* is a mutant of Pdia4 whose three CGHC motifs were changed into three SGHS motifs (a*, a’*, and a’’*).FThe construct encoding Myc/Flag‐tagged p22^phox^, Ndufs3 or their mutants and that expressing Flag‐tagged Pdia4 were co‐transfected into 293T cells. Total lysates (TL) and anti‐Myc immunoprecipitates (Myc IP) underwent immunoblotting analysis using anti‐Flag antibody. Source data are available online for this figure.

To decode the molecular basis of Pdia4 for ROS production in β‐cells, we teased out the interaction of Pdia4 with the ROS‐generating machinery such as the mitochondrial ETC, Nox, and Ero1 pathways. Proteomic analysis of the Pdia4 and mock precipitates in Min6 cells identified Ndufs3, a component of ETC CI, and, p22^phox^, a common component of Nox 1 to 4, but not Ero1 as potential interacting partners of Pdia4 (data deposited at PeptideAtlas (PASS01396). Consistently, immunoprecipitation assays showed that Pdia4 bound Ndufs3 (top left, Fig 6A) and p22^phox^ in Min6 cells where those three were endogenous proteins (right, Fig 6A). However, Pdia4 did not bind Ero1 (bottom left, Fig 6A) or the component of ETC CII to IV (top left, Fig 6A). Confocal data showed that Pdia4 was colocalized with Ndufs3 and p22^phox^ in the mitochondria and membrane of Min6 cells, respectively (Fig 6B). To further verify the interaction of Pdia4 with Ndufs3 and p22^phox^, we co‐expressed Pdia4 and Ndufs3 or p22^phox^ with different tags at their N‐terminus in 293T cells. We confirmed this interaction in 293T cells using those proteins with different tags (Fig 6C). Further, we tested the *in vitro* direct interaction between Pdia4 and its partners. Pull‐down assays revealed that Pdia4 specifically and directly bound to Gst‐Ndufs3 and Gst‐p22^phox^ but not Gst‐TecSH3, an irrelevant protein (Fig 6D), and Nox 1 to 4 (Appendix Fig S7A). We also explored the action of Pdia4 on the stability of Ndufs3 and p22^phox^ in the presence of protease (Appendix Fig S7B). The co‐incubation assays showed that Pdia4 protected against the trypsin‐mediated degradation of Ndufs3 (top, Appendix Fig S7B) and p22^phox^ (bottom, Appendix Fig S7B) more than an irrelevant protein, bovine serum albumin (BSA), implying that Pdia4 stabilized the ROS‐generating pathways.

To pinpoint the interaction domain in Pdia4, Ndufs3, and P22^phox^, 293T cells co‐expressing Flag‐tagged Pdia4 or its deletion/point mutation mutants and full‐length Ndufs3 or p22^phox^ with Myc/Flag tags were tested. Co‐immunoprecipitation experiments using Myc antibody showed that the first two CGHC domains (a and a') of Pdia4 were mainly responsible for its interaction with Ndufs3 (left, Fig 6E) and p22^phox^ (right, Fig 6E). In contrast, non‐catalytic domains (b and b') of Pdia4 failed to bind Ndufs3 and p22^phox^ (Pdia4^322–515^, Fig 6E). However, conversion of CGHC into SGHS in the three CGHC domains of Pdia4 resulted in the loss of its ability to bind Ndufs3 and p22^phox^ (Pdia4*, Fig 6E). The same strategy was used to characterize the interaction domain of Ndufs3 or p22^phox^ for Pdia4. The results showed that the domain corresponding to the first 132 amino acids of Ndufs3 (truncated Ndufs3, tNdufs3) or the domain corresponding to the first 99 amino acids of p22^phox^ (truncated p22^phox^, tp22^phox^) was the domain for Pdia4 association (Fig 6F). Overall, these data suggest that Pdia4 interacts with Ndufs3 and p22^phox^ in a CGHC motif‐dependent fashion.

### Molecular regulation of ROS generation by Pdia4 in pancreatic β‐cells

**Figure 7:**
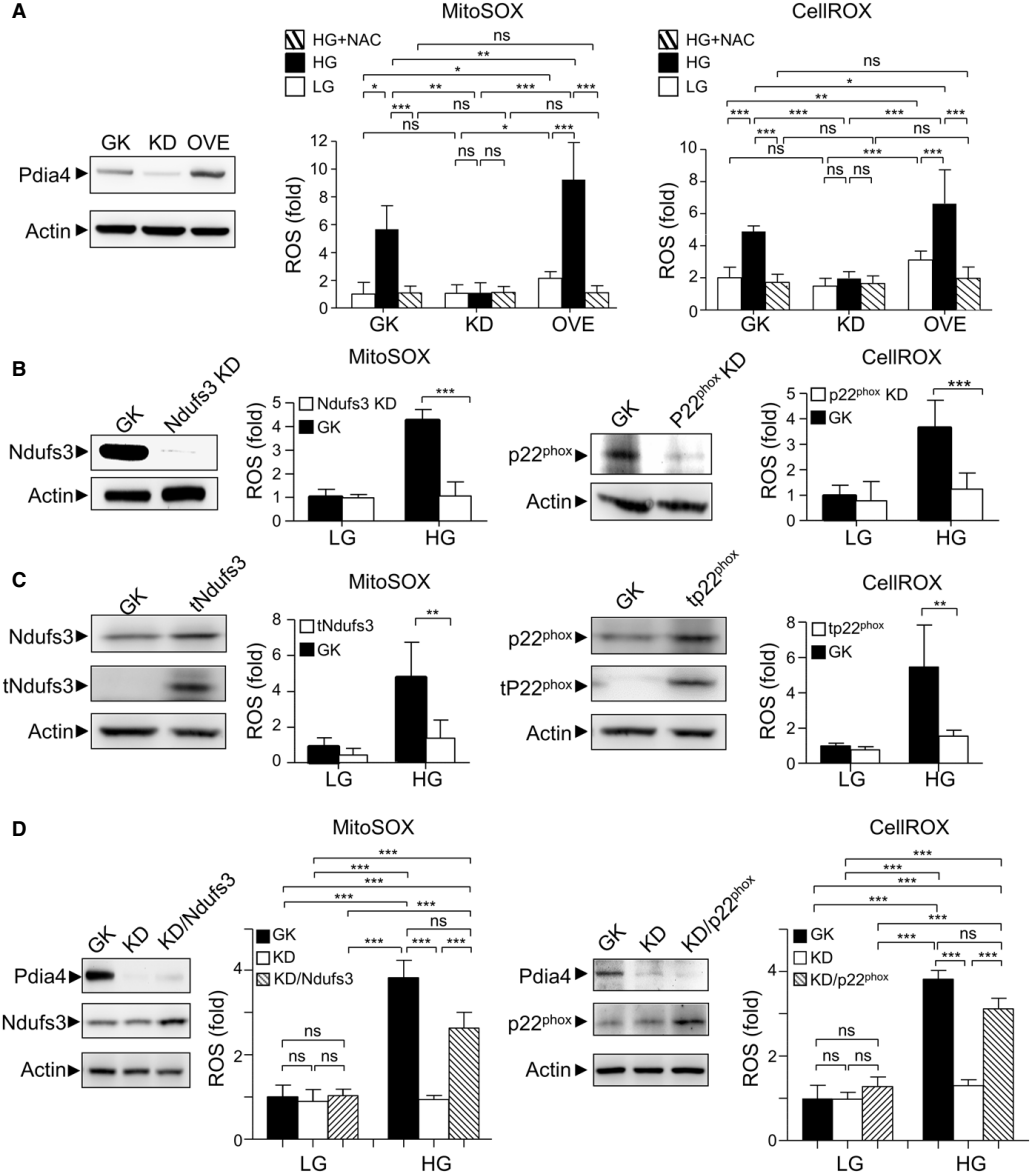
Pdia4 regulates ROS generation involving Ndufs3 and p22^phox^ in Min6 cells AMin6 cells infected with a lentivirus expressing a scramble RNAi (GK), a Pdia4 RNAi (KD), and a Pdia4 cDNA (OVE) were sorted and tested for the Pdia4 protein level (left). The cells were incubated with NAC (1 mM) and then stained with MitoSOX or CellROX in response to 0.5 mM (LG) and 25 mM (HG) glucose for an additional 30 min. Signal from MitoSOX (middle) and CellROX (right) was re‐plotted into histograms.BMin6 cells infected with a lentivirus expressing a scramble RNAi (GK) and an RNAi of Ndufs3 (Ndufs3 KD) or p22^phox^ (p22^phox^ KD) were selected and tested for levels of Ndufs3 (1^st^ column), mitochondrial ROS (2^nd^ column, MitoSOX), p22^phox^ (3^rd^ column), and cytosolic ROS (4^th^ column, CellROX). The cells were grown in the presence of 0.5 mM (LG) and 25 mM (HG) glucose.CThe same experiments as (B) were conducted except that Min6 cells were infected with a lentivirus expressing a cDNA of truncated Ndufs3 (tNdufs3) and p22^phox^ (tp22^phox^).DMin6 GK or KD cells infected with a lentivirus expressing a cDNA of full‐length Ndufs3 or p22^phox^ were selected and tested for the level of Pdia4 and Ndufs3 (1^st^ column), mitochondrial ROS (2^nd^ column, MitoSOX), Pdia4 and p22^phox^ (3^rd^ column), and cytosolic ROS (4^th^ column, CellROX). The cells were grown in the presence of 0.5 mM (LG) and 25 mM (HG) glucose. Data information: Data from 3 experiments are presented as the mean ± SD. One‐way ANOVA test was used for statistical analysis of differences between groups, and *P* (*) < 0.05; *P* (**) < 0.01; and *P* (***) < 0.001 are considered statistically significant.

Furthermore, we wanted to address how Pdia4 controlled β‐cell ROS production via ETC and Nox pathways. First, a genetic approach was used to examine the function of Pdia4 in ROS production. The content of mitochondrial and cytosolic ROS in Min6 GK cells, a scramble control, Min6 KD cells with Pdia4 knockdown, and Min6 OVE cells with Pdia4 overexpression, was measured using the MitoSOX and CellROX dyes, respectively. We found that Min6 OVE cells had a slight increase in mitochondrial and cytosolic ROS compared with Min6 GK and Min6 KD cells in response to low glucose (LG, Fig 7A). Accordingly, Min6 OVE cells had higher ROS content in the mitochondria and cytosol than Min6 GK and KD cells in response to high glucose (HG, Fig 7A). However, N‐acetylcysteine (NAC), a ROS scavenger, abolished the increase of Pdia4‐mediated ROS production in the mitochondria and cytosol in all Min6 lines (HG+NAC, Fig 7A). Next, we assessed the involvement of Ndufs3 and p22^phox^ in ROS production in the mitochondria and cytosol of Min6 cells. The siRNA experiments showed that knockdown of Ndufs3 and p22^phox^ reduced the mitochondrial and cytosolic ROS content in Min6 cells, respectively (Fig 7B).

Next, to test the likely link among Pdia4, Ndufs3, p22^phox^, and ROS production, we examined whether overexpression of tNdufs3, a truncated mutant of Ndufs3 (a.a. 1 to 132), and tp22^phox^, a truncated mutant of p22^phox^ Ndufs3 (a.a. 1 to 99), interfered with ROS production in Min6 cells. The data indicated that overexpression of tNdufs3 and tp22^phox^ caused a significant reduction in mitochondrial and cytosolic ROS content in Min6 cells at a high glucose concentration (HG, Fig 7C). However, this reduction was not significant at a low glucose concentration (LG, Fig 7C). In parallel, we overexpressed Ndufs3 and p22^phox^ in Min6 KD cells in which Pdia4 was knocked down. The overexpression of Ndufs3 and p22^phox^ could restore the mitochondrial and cytosolic ROS production in Min6 KD cells to a comparable level of ROS production in Min6 GK cells in the presence of high glucose (HG, Fig 7D). This restoration was also seen in low glucose (LG, Fig 7D) though it was not statistically significant. Therefore, the genetic data supported the notion that Pdia4 up‐modulated Ndufs3‐ and p22^phox^‐mediated ROS generation via their mutual association.

### Identification and effect of GHTT, a Pdia4 inhibitor, on diabetic reversal in *Lepr^db/db^* mice

**Figure 8:**
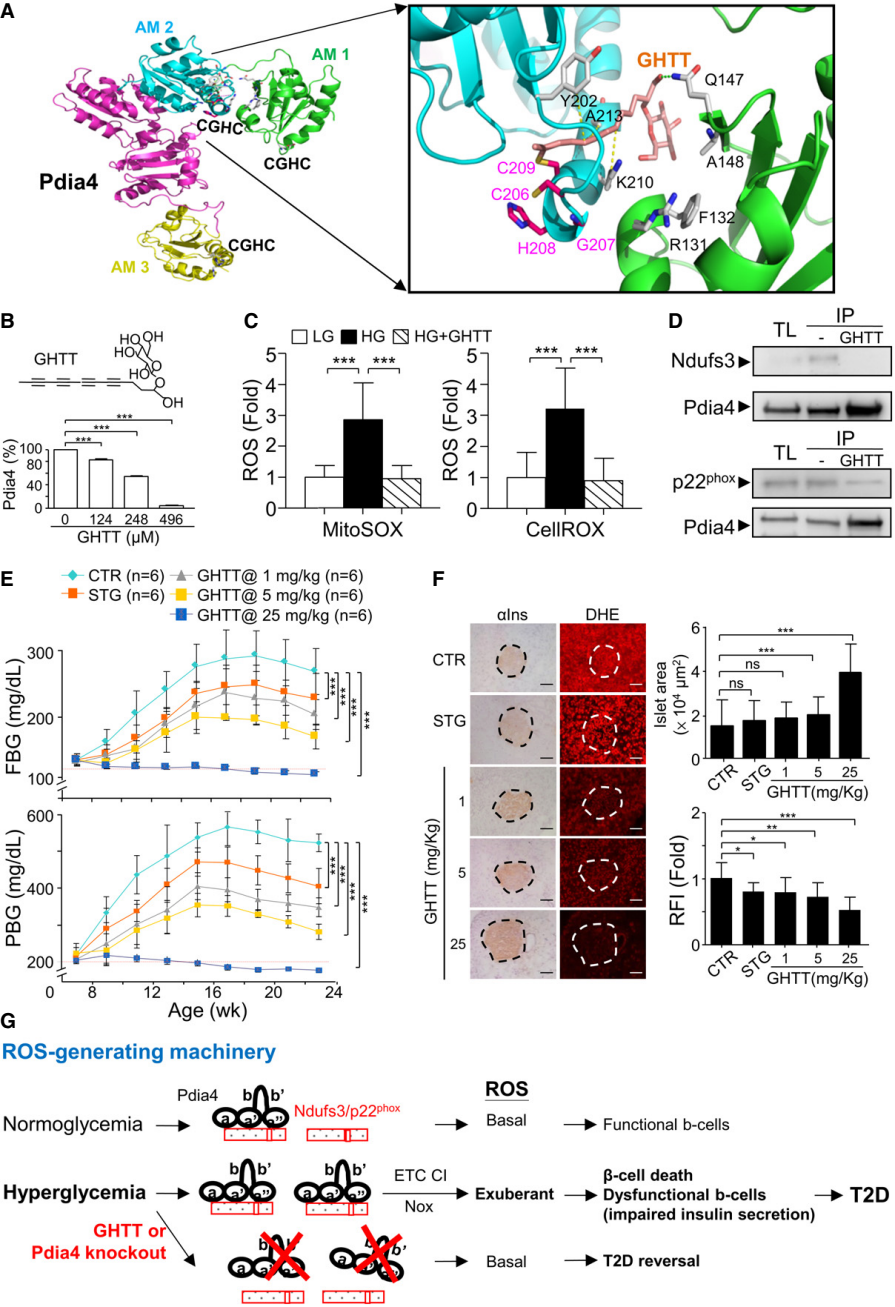
Effect of GHTT on diabetes development and ROS production in *Lepr^db/db^* mice AMolecular docking indicating the interaction between GHTT and active motifs (AMs) of Pdia4. AM 1, 2, and 3 represent the first, second, and third CGHC domains. The sulfur, nitrogen, and oxygen atoms are shown in yellow, blue, and red, respectively. The hydrogen bond and hydrophobic interaction between GHTT (light pink) and the amino acid residues (gray) of Pdia4 model are shown by the green and yellow dashed lines, respectively.BInsulin‐based turbidity assays were used. Insulin was incubated with recombinant Pdia4 in the presence of PBS and inhibitors at 25°C. After 30 min, the reaction was stopped and measured the absorbance at 595 nm. The relative activity (%) of Pdia4 was obtained by the formula, 100% × (OD_595_ of PBS − OD_595_ of GHTT)/(OD_595_ of PBS).CMin6 cells were incubated with medium containing low glucose (LG, 0.5 mM) and high glucose (HG, 25 mM) in the absence or presence of 28 μM GHTT (HG+GHTT) and then stained with MitoSOX or CellROX for an additional 30 min. Signal from CellROX (right) and MitoSOX (left) was quantified and re‐plotted into histograms.D293T cells, which were transfected with the construct encoding Flag‐Pdia4 and that expressing Myc/Flag‐tagged p22^phox^ or Ndufs3, were treated with GHTT (28 μM) for 30 min. The cells were lysed and incubated with anti‐Pdia4 antibodies plus protein G beads. Their total lysates (TL) and immunoprecipitates (IP) underwent immunoblotting analysis with anti‐Flag antibody.EEight‐week‐old diabetic *Lepr^db/db^* mice were orally fed with a daily dose of PBS (CTR), sitagliptin (STG, 90 mg/kg) and GHTT (1, 5, and 25 mg/kg) from 8 to 24 weeks. Their FBG and PBG were measured.FThe sections of pancreata of the mice (E) were stained with anti‐insulin (αIns) antibody and dihydroethidium (DHE) (left). Islet area (μm^2^) and relative fluorescence intensity (RFI) were quantified (right). Scale bar: 100 μm. The dash circles indicate islet regions.GA schema illustrating the regulation of Ndufs3 and p22^phox^ by Pdia4 via a CGHC‐dependent intermolecular interaction. Pdia4 interacts with Ndufs3 and p22^phox^, leading to an increased activity of ETC CI and Nox. Consequently, the exuberant ROS induces β‐cell pathology and diabetes. Conversely, Pdia4 deficiency and inactivation of Pdia4 by GHTT can abolish the interaction of Pdia4 and p22^phox^ or Ndufs3, leading to reduction of ROS production and β‐cell failure and mitigation of diabetes. Data information: Data from three experiments (B, C and F) and more (E) are presented as the mean ± SD. Log rank (E) and one‐way ANOVA test (B, C and F) were used for statistical analysis of differences between groups, and *P* (*) < 0.05; *P* (**) < 0.01; and *P* (***) < 0.001 are considered statistically significant. The number of mice (*n*) is indicated in parentheses. Source data are available online for this figure.

Finally, we checked whether Pdia4 inhibitors could rescue β‐cells from death and, in turn, reverse diabetes in diabetic mice. We first screened for Pdia4 inhibitors using the virtual screening of the Pdia4 binding pocket in 261 in‐house phytocompounds (Fig 8A) as described (Almeida *et al*, 2011; Kuo *et al*, 2017). GHTT, one of the best hits, was tested for Pdia4 activity and had an inhibitory activity with an IC_50_ of 358 μM (Fig 8B). Then, we explored the impact of GHTT on ROS production and ROS‐generating pathways. We found that GHTT lowered ROS in the cytosol and mitochondria of Ming 6 cells (Fig 8C). Furthermore, GHTT reduced the interaction between Pdia4 and Ndufs3 or p22phox (Fig 8D).

Moreover, we examined the *in vivo* anti‐diabetic effect of GHTT in new‐onset diabetic *Lepr^db/db^* mice. As anticipated, 90 mg/kg of sitagliptin, which was used as a positive control, moderately reduced FBG and PBG in *Lepr^db/db^* mice (STG, Fig 8E). GHTT normalized diabetes in a dose‐dependent manner as evidenced by FBG (top, Fig 8E), PBG (bottom, Fig 8E), GTT (Appendix Fig S8A), and Hb_A1c_ (Appendix Fig S8B). Accordingly, GHTT dose‐dependently reduced islet atrophy (Fig 8F), islet ROS (Fig 8F), and serum ROS (Appendix Fig S8C) in *Lepr^db/db^* mice. However, sitagliptin failed to reduce islet atrophy (Fig 8F). Preservation of the islets by GHTT was in agreement with HOMA‐β (Appendix Fig S8D) and serum insulin (Appendix Fig S8E). Overall, the data demonstrated that the Pdia4 inhibitor, GHTT, phenocopied its genetic knockout in mouse models.

In conclusion, this work suggested that under normoglycemic conditions, Pdia4 produced a basal level of ROS to maintain β‐cell mass and function via its interaction and stabilization of Ndufs3 and p22^phox^ (Fig 8G). Under hyperglycemic conditions, excess nutrient up‐regulated Pdia4 expression. This up‐regulation increased the activity of Ndufs3 and p22^phox^, via intermolecular interaction, and, subsequently, ROS production in β‐cells. Consequently, the exuberant ROS resulted in β‐cell pathogenesis and diabetes (Fig 8G). Conversely, Pdia4 inhibitor reversed diabetes via decreased β‐cell pathology and ROS production in diabetic animals (Fig 8G). These findings also revealed the underlying mechanism and function of Pdia4 in β‐cells and diabetes as well as the likely use of Pdia4 inhibitors as an anti‐diabetic therapy.

## Discussion

Pancreatic β‐cell failure is a hallmark of diabetes (Cerf, 2013). Nevertheless, the mechanism through which this failure occurs is poorly investigated. In this study, we conducted comprehensive phenotyping of a previously unrecognized factor, Pdia4, in glucose homeostasis in two mouse models of obesity‐related diabetes. In mouse models and islet mass, deletion of Pdia4, either globally or in β‐cells, led to protection from hyperglycemia, most likely due to a decrease in oxidative stress. Enrichment of Pdia4 in the β‐cells of mice resulted in accelerated diabetes development and failure to accrue islet mass. Consistently, the Pdia4 inhibitor, GHTT, protected against hyperglycemia in the mouse models. Mechanistically, Pdia4 was shown to interact with Ndufs3 and p22^phox^ in a sequence‐specific fashion. The Pdia4, Ndufs3, and p22^phox^ pathways were confirmed to dictate ROS production in endocrine β‐cells. We conceptualized these findings as follows: under pathological conditions, excessive Pdia4 and ROS led to β‐cell failure and diabetes; conversely, Pdia4 ablation and inhibition diminished ROS content in β‐cells and, thereby, reduced β‐cell failure and diabetes development. In essence, exuberant ROS is detrimental to β‐cells, and getting rid of Pdia4 improves ROS.

This work unraveled the molecular mechanism through which Pdia4 resulted in a decrease in functional β‐cell mass during diabetes. We demonstrated that Pdia4 positively regulated β‐cell dysfunction/death during diabetes. First, PI staining data indicated that, consistent with ROS production, Pdia4 promoted cell death in β‐cells (Fig 4A). Furthermore, Pdia4 significantly increased the expression of two markers, Beclin 1, a key player in the initiation step of autophagy, and LC3‐II, a player in the elongation step of this process, during autophagy in β‐cells (Fig 4B) in agreement with the literature stating that autophagy is linked to diabetes (Yang *et al*, 2017). Since β‐cell dedifferentiation has been reported to participate in β‐cell failure (Kim‐Muller *et al*, 2016; Efrat, 2019), we also examined the relationship between Pdia4 and Aldh1a3, a dedifferentiation marker of β‐cells. We found that Pdia4 increased the expression level of Aldh1a3 in islet cells (Appendix Fig S9B). Thus, Pdia4 contributed to cell death in β‐cells in a manner that involved autophagy (Fig 4) and dedifferentiation (Appendix Fig S9B) but not JNK (Appendix Fig S9A) in β‐cells. However, we found that Pdia4 did not affect β‐cell proliferation (BrdU, Fig 2D) and differentiation (PDX1 and MafA, Appendix Fig S9C). In this context, the significance and mechanisms of Pdia4 in β‐cell physiopathology from the perspective of dedifferentiation and autophagy are worth further investigation. We also showed that Pdia4 was associated with dysfunction in β‐cells through GSIS (Figs 2F and 3D), insulin content (Fig 3E), insulin granules (Fig 5A and C), and mitochondrial parameters (Fig 5A and B). This dysfunction could be attributed to the up‐regulation of ROS by Pdia4 because ROS is known to impair mitochondrial structure/function and insulin production/secretion in β‐cells (Gerber & Rutter, 2017). However, Pdia4 failed to regulate the expression of antioxidant proteins (Appendix Fig S6E). According to these data, we concluded that Pdia4 increased ROS and, thereby, caused β‐cell dysfunction and death. Mechanistically speaking, Pdia4 up‐regulated ROS accumulation in response to glucose in β‐cells (Fig 7) via the interaction of Pdia4 with Ndufs3 and p22^phox^, key components of ETC CI and Nox 1‐4, respectively (Fig 6). This intermolecular interaction involved the first two CGHC motifs of Pdia4 and the N‐terminal portion of Ndufs3 or p22^phox^ (Fig 6). In addition, this interaction seemed to increase the stability of Ndufs3 or p22^phox^ in the presence of trypsin (Appendix Fig S7B). We also established the new link between Pdia4, Ndufs3, p22^phox^, and ROS production in β‐cells based on the interference and complementation data (Fig 7). Accordingly, disruption of this interaction by GHTT, the Pdia4 inhibitor, led to decreased ROS in β‐cells (Fig 8C and D). Both the genetics and pharmaceutical data are consistent with several publications that have reported the increased expression and/or activity of Ndufs3 and p22^phox^ in diabetic patients and rodents (Huang *et al*, 2011; Wu *et al*, 2017). However, a caveat in the genetics approaches used in this study was that Pdia4 was deficient or overexpressed from the inception of β‐cell development. In parallel, treatment with GHTT reduced islet atrophy in β‐cells (Fig 8F). Pharmaceutical approaches complemented the genetics approaches in terms of β‐cell improvement. Therefore, this work demonstrates the novel function and molecular basis of the chaperone, Pdia4, in β‐cells in terms of oxidative stress, dysfunction, and death and extends our understanding of islet biology.

The findings presented further suggest the potential of Pdia4 to serve as a therapeutic target for obesity‐related diabetes and β‐cell pathology. *Lepr^ab/db^* mice and HFD‐fed B6 mice were used as mouse models of diabetes to study the significance of Pdia4 in β‐cell failure and diabetes because *Lepr^ab/db^* mice develop severe diabetes while B6 mice fed a high‐fat diet develop moderate diabetes. First, Pdia4 ablation led to a remarkable reduction in diabetes incidence (58%) in *Pdia4^−/−^Lepr^db/db^* mice aged 24 weeks (*Pdia4^−/−^Lepr^db/db^*, Appendix Fig S2E). This incidence went down to zero in *Lepr^db/db^* mice aged 55 weeks or over (Appendix Fig S3B) presumably because food consumption decreased with age (Appendix Fig S3A). Consequently, *Pdia4^−/−^Lepr^db/db^* mice had a better survival rate and life span than *Lepr^db/db^* mice (Fig 2A). Accordingly, GHTT treatment phenocopied Pdia4 knockout in *Lepr^db/db^* mice (Fig 8). Conversely, Pdia4 overexpression accelerated diabetes in Pdia4^tg/tg^ *Lepr^db/db^* mice and HFD‐fed Pdia4^tg/tg^ B6 mice (Appendix Figs S5 and S6). In addition, Pdia4^tg/tg^ *Lepr^db/db^* mice had a worse survival rate and life span than *Lepr^db/db^* mice (Fig 2A). Of note, Pdia4 deficiency and inactivation also improved β‐cell failure and diabetes. The reversal of diabetes in aged *Pdia4^−/−^Lepr^db/db^* mice most probably came from reduced β‐cell death by Pdia4 and/or replenishment of β‐cells (Gao *et al*, 2015). Although these findings with *Lepr^db/db^* mice are fascinating, their relevance to human diabetes remains tentative. The role of Pdia4 in HFD‐fed B6 mice was further verified because this model resembles human diabetes more closely than *Lepr^db/db^* mice in terms of disease etiology (Hinder *et al*, 2017). Both models of diabetes complemented each other and revealed the importance and therapeutic potential of Pdia4 for diabetes and β‐cell failure. Targeting Pdia4 and its pathways may thus constitute attractive approaches for the treatment of β‐cell pathogenesis and diabetes (Leahy *et al*, 2010).

Oxidative stress is well recognized to play a major role in β‐cell physiopathology and diabetes (Dos Santos *et al*, 2019). Homeostasis of ROS is controlled by ROS‐generating and ROS‐degrading pathways. A basal level of ROS is essential for β‐cell physiology and function (Trachootham *et al*, 2009). However, metabolic overload stimulates ROS overproduction which impairs insulin secretion and insulin action during diabetes (Tangvarasittichai, 2015). This impairment can get worse if antioxidant enzymes are insufficient. Our current work agrees with the literature stating that elevated ROS, arising from increased oxidative stress, contributes to β‐cell failure and diabetes development (Lenzen *et al*, 1996; Tiedge *et al*, 1997; Robertson, 2004; Robertson & Harmon, 2007). Although the concept of ROS causing β‐cell failure is not a new concept, the participation of Pdia4 in the Ndufs3 and p22^Phox^ pathways gives greater understanding of the progression from oxidative stress to β‐cell pathology and diabetes. Overall our work can be viewed as follows: excess nutrients/hyperglycemia → Pdia4 → Ndufs3 and p22^phox^ → ROS → β‐cell failure and diabetes (Fig 8). ROS acts as a double‐edged mediator of β‐cell physiopathology and diabetes. Thus, antioxidant compounds and enzymes are commonly used to scavenge excessive ROS during diabetes in pre‐clinical and clinical settings (Robertson, 2004; Robertson & Harmon, 2007; Chang & Chuang, 2010). However, this approach has frequently failed to treat diabetes in diabetic mice and patients (Chang & Chuang, 2010) likely because some ROS might have already exerted its action before its clearance by antioxidants. In this study, targeting Pdia4 seemed to have some advantage over the using antioxidant compounds and enzymes because Pdia4 deficiency eliminated the possibility of diabetes development by lowering ROS production, different from elimination of ROS by antioxidants, in which the cells continue to produce ROS through the ROS‐generating machinery. So far, no drugs can clinically cure diabetes (Leahy *et al*, 2010). Thus, preserving β‐cell number and/or function by intervention in the Pdia4/Ndufs3/p22^Phox^ pathways is a promising therapy.

With regard to advances in knowledge of Pdia4 in general, Ndufs3 and p22^phox^ are recognized as specific partners/substrates of Pdia4 in ROS‐generating pathways in β‐cells for the first time in this study. Pdia4 regulated mitochondrial and cytosolic ROS production via its binding to Ndufs3 and p22^phox^, respectively (Figs 4, 6, 7, and 8). Pdia4 served as a chaperone to enhance the stability and/or activity of mitochondrial Ndufs3 or cytosolic p22^phox^ outside the ER compartment. Furthermore, the active site and interacting motifs in Pdia4 and their partners were identified (Fig 6). These findings pave the way for understanding how a chaperone like Pdia4 is engaged in regulation of oxidative stress. Pdia4 possesses a C‐terminal KEEL motif and was originally considered to be an ER‐resident protein chaperone that could assist in protein folding. (Ni & Lee, 2007; Galligan & Petersen, 2012). This study pushes the boundary of understanding of Pdia4 from purely ER‐relevant functions to an unconventional ER‐irrelevant function in β‐cells (Figs 1G and H, and 6B) and supports the novel notion that a chaperone with the ER retention motif can reach other subcellular compartments for their physiological needs. Pdia4 is demonstrated to utilize a hitherto undocumented mechanism to modulate oxidative stress in β‐cells. This mechanism is distinguished from those in which ER stress proteins, antioxidant enzymes, and ser/thr kinases, regulate β‐cell functions, survival, and/or cell death via modulation of ER stress, ROS, and phosphorylation (Lei & Vatamaniuk, 2011; Ardestani & Maedler, 2016; Herbert & Laybutt, 2016). We also figured out the reason why Pdia4 was named glucose‐regulated protein 72 (Fig 1 and Appendix Fig S1). There is an ER stress‐response element (ERSE) present in the Pdia4 promoter that functionally responds to high glucose (Appendix Fig S1C). One piece of our data also shed light on the expression of Pdia4 in mouse organs and its distribution in β‐cell compartments. In comparison with other Pdis, Pdia4 was found to be more specific in terms of substrate specificity and functional non‐redundancy (Appendix Fig S7). Finally, the data on the unique mode of action and substrate specificity proposed that Pdia4, a non‐essential gene, are indeed druggable.

Overall we believe the data presented provide conceptual advances in Pdia4 research and the application of β‐cells and diabetes. Furthermore, this work presents a compelling case for the further investigation of Pdia4 as a crucial player of β‐cell pathogenesis and diabetes.

## Materials and Methods

### Plasmid construction and lentiviral infection

The pLKO.1‐GFP lentiviral vectors, GK, KD, OVE, Ndufs3 KD, p22^phox^ KD, Ndufs3/tNdufs3, and p22^phox^/tp22^phox^, contained a scramble RNAi, a Pdia4 RNAi sequence, a Pdia4 cDNA, a Ndufs3 RNAi sequence, a p22^phox^ RNAi sequence, a full‐length/truncated Ndufs3 cDNA, and a full‐length/truncated p22^phox^ cDNA, respectively (Appendix Tables S1 and S2). To produce lentiviral particles, HEK 293T cells were transfected with lentiviral plasmids plus packaging plasmids using the TransIT‐LT1 reagent (Mirus Bio, WI) as described elsewhere (Kuo *et al*, 2017). A FACSAria cell sorter (BD Biosciences) was used to isolate stable cells for further use. The plasmids encoding Flag‐tagged Pdia4 and its deletion/point mutation mutants, Myc/Flag‐tagged Ndufs3, p22^phox^, and their deletion mutants were constructed using PCR cloning.

### Reagents and cells

DHE, DAPI, CellROX, MitoGreen, MitoRed, MitoSOX, Hochest 33342, and chloromethyl‐2',7'‐dichlorodihydrofluorescein diacetate (CM‐H_2_DCFDA) were purchased from Molecular Probes (Eugene, OR). Glucose, BSA, trypsin, palmitate, NADPH, and histopaque‐1077 were purchased from Sigma (St. Louis, MO). Lucigenin, and collagenase P were from Roche (Switzerland). GHTT was purified as described previously (Almeida *et al*, 2011; Kuo *et al*, 2017). Recombinant proteins, 6×His‐Pdia4, Gst‐p22^phox^ and Gst‐Ndufs3 were purchased from Enzo (Farmingdale, NY). Gst‐TecSH3, containing a SH3 domain of Tec, was produced as published (Kuo *et al*, 2017). The antibodies used in this study were purchased (Appendix Table S3). 293T cells (CRL‐3216), Min6 cells (Yagi *et al*, 1995), and pancreatic islets were grown in complete DMEM medium (Thermo Fisher, Waltham, MA) containing 10% and 20% fetal bovine serum (FBS), respectively, and 3.3 mM glucose unless indicated otherwise. Human islets were purchased from Lonza (Switzerland) and handled according to the protocol of the Academia Sinica Institutional Review Board (AS‐IRB01‐14015). Informed consent was obtained from all human subjects and the experiments conformed to the principles of the WMA Declaration of Helsinki and the Department of Health and Human Services Belmont Report.

### Generation of conventional knockout mice, conditional knockout, and transgenic mice

B6, BKS (Jackson Laboratory stock No. 000662), and B6.BKS(D)‐*Lepr^db^*/J (Jackson Laboratory stock No. 000697) were purchased from the Jackson laboratory. To generate conventional knockout mice (Appendix Fig S2), *Pdia4* gene targeting vector was first constructed using a bacterial artificial chromosome (BAC) recombineering strategy. Briefly, a 230‐kb Bac clone contains an entire allele of mouse *Pdia4* gene as indicated. The first Neo cassette containing two homology arms, two loxP sites (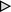) and a Neo gene underwent homologous recombination and was inserted into the intron 6 of *Pdia4* gene in the Bac. The first Neo cassette in the recombined Bac was popped out by *in vitro* incubation with Cre recombinase. The second Neo cassette containing two homology arms, two frt sites (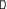), one loxP site (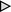), and a Neo gene was inserted into the intron 2 of *Pdia4* gene in the Bac. Following linearization, this BAC construct was electroporated into B6 ES cells. After recombination screening, the targeted ES lines were selected for blastocyst injection and, in turn, generation of chimeric mice. Chimeric mice were bred with B6 mice to obtain *Pdia4^f/+^* mice. *Pdia4^f/f^* mice were crossed with *EIIa‐Cre* deleter (Jackson Laboratory stock No. 003724) mice to obtain the *Pdia4^+/−^* mice whose exons 3 to 6 were deleted. Sibling mating of *Pdia4^+/−^* mice produced wild‐type (*Pdia4^+/+^*), *Pdia4^+/−^* and *Pdia4^−/−^* mice. Alternatively, *pdia4^f/f^* mice were crossed with Ins2‐Cre deleter mice (Jackson Laboratory stock No. 003573), a transgenic B6 composed of the mouse insulin 2 promoter linked to a Cre cDNA to obtain *Pdia4^f/f^* Cre^tg/tg^ B6 mice, the islet‐specific (conditional) knockout mice (Appendix Fig S4). To generate Pdia4 transgenic mice (Appendix Fig S5), a vector composed of the human insulin (hINS) promoter linked to a human Pdia4 cDNA was constructed. A linearized KpnI/DraIII fragment from this vector was microinjected into the pronuclei of B6 fertilized eggs, followed by oviduct transfer into surrogate mothers. One line (Pdia4^tg/tg^ mice) was selected and crossed with B6 mice. To breed B6 background to a *Lepr^db/db^* background, the B6 mice with Pdia4 knockout and overexpression were first crossed with BKS mice and this backcrossing was repeated until the SNPs of BKS mice were confirmed as published (Genomics 83 (2004) 902‐911). The resultant mice were then crossed with B6.BKS(D)‐*Lepr^db^*/J mice in order to obtain *Pdia4^−/−^Lepr^db/db^* and Pdia4^tg/tg^ *Lepr^db/db^* mice. Similarly, *Pdia4^f/f^* Cre^tg/tg^ B6 mice were bred into BKS mice and crossed with B6.BKS(D)‐*Lepr^db^*/J mice in order to obtain *Pdia4^f/f^* Cre^tg/tg^ *Lepr^db/db^* mice. Moreover, Pdia4 knockout, conditional knockout, and transgenic mice on *Lepr^db/db^* background were analyzed using genetic confirmation as published (Mao *et al*, 2006). The BKS mice were used as control littermates. All the mouse lines were confirmed using PCR, Southern blots or immunoblots. All animals had free access to chow and water, and were maintained at 21–23°C with 12 h light‐12 h dark cycles in the institutional animal facility. The experiments of B6, BKS, and *Lepr^db/db^* females at the indicated ages for diabetes were approved by the Academia Sinica Institutional Animal Care and Utilization Committee (11‐03‐158).

### Drug administration and measurement of metabolic parameters

WT, *Pdia4^−/−^*, and *Pdia4^tg/tg^* B6 mice had free access to a high‐fat diet (60% of fat) from 4 to 28 weeks of age. Their FBG, PBG, and Hb_A1c_ were measured using a glucometer and a DCA 2000 analyzer (Bayer, Germany) at the indicated ages as published (Chang *et al*, 2013). Food intake, water consumption, survival rate, diabetic incidence, and lifespan were monitored weekly. For glucose tolerance test (GTT), the mice at the indicated ages were fasted for 16 h. The mice received an intraperitoneal injection with glucose (1 g/kg). The levels of blood glucose were measured using blood samples taken from the tail vein at 0, 30, 60, 120, and 180 min after glucose injection with an Elite glucometer. Homeostatic model assessment of β‐cell function (HOMA‐β) and that of insulin resistance (HOMA‐IR) were calculated using the following formulae: HOMA‐β = 20 × fasting insulin (mU/ml)/[fasting glucose (mmol/l) − 3.5] and HOMA‐IR = fasting glucose (mmol/l) × fasting insulin (μU/ml)/22.5. Hb_A1C_, immunohistochemical staining, serum insulin, and serum ROS were measured as described in the Materials and Methods section. Alternatively, 8‐week‐old diabetic *Lepr^db/db^* mice were daily fed with GHTT and sitagliptin at the indicated dosages from 8 to 24 weeks. Their GTT, Hb_A1c_, serum ROS, HOMA indices, and serum insulin were monitored.

To compare the efficacy of ROS scavenger, vitamin C, and Pdia4 ablation in diabetes, *Lepr^db/db^* mice were fed with feed containing a daily dose of vitamin C at 42, 125, and 375 mg/kg. To assess the effect of food restriction on diabetes, *Pdia4^−/−^Lepr^db/db^* mice were given feed (6.8 g/mouse/day). The mice were monitored for their diabetic incidence based on post‐meal glucose.

### Measurement of insulin and serum Pdia4

The level of serum insulin was determined by ELISA assays as published previously (Chang *et al*, 2013). Alternatively, mouse pancreata were digested with collagenase P (Roche, Switzerland) and harvested with histopaque‐1077 gradient centrifugation. For GSIS, mouse pancreatic islets were isolated and pre‐incubated in serum‐free oxygen‐saturated Krebs‐Ringer bicarbonate (KRB) buffer containing 3.3 mM glucose at 37°C for 30 min. The islets (5 islets/well) were then incubated with KRB buffer containing high glucose (16.7 mM) or low glucose (3.3 mM) for an additional 30 min. The supernatants were collected for insulin ELISA assays. To detect the insulin content in pancreatic islets, fasted 14‐week‐old WT, *Pdia4^−/−^* and Pdia4^tg/tg^ BKS mice were intraperitoneally injected with glucose (1 g/kg body weight). After 0.5 h, the mouse islets were isolated and divided into 2 aliquots. One aliquot of the islets was treated with GolgiPlug, followed by intracellular staining with an anti‐insulin antibody (0 h). The other aliquot of the islets were grown in complete DMEM medium plus 3.3 mM glucose for 24 h and GolgiPlug was added 6 h before insulin staining. The islets underwent intracellular staining with an anti‐insulin antibody (24 h). Both aliquots of the islets underwent flow cytometry analysis. The level of serum Pdia4 was determined by an ELISA kit (LSBio, WA).

### Real‐time poly chain reaction (RT–PCR)

Total RNA of Min6 cells and mouse pancreatic islets were extracted and converted to cDNA. Real‐time RT–PCR was performed with the above cDNA using the primer sets (Appendix Table S4). The relative expression level (REL) of typical Pdis versus atypical Pdis was quantified in relation to the level of L13.

### Transmission electron microscopy

Pancreatic islets were isolated from mouse lines, fixed with formaldehyde, and embedded with resin. The samples underwent ultramicrotomy, heavy metal staining, and observation under Tecnai G2 F20 TEM. β‐cells were recognized by their typical appearance and photographed. The number and average area of mitochondria and insulin granules in β‐cells were quantified using Image J software.

### Immunohistochemical (IHC) analysis

Mouse pancreata were removed and frozen in optimal cutting temperature medium. For insulin staining, the sections were incubated at 25°C for 30 min with anti‐insulin antibody, followed by diaminobenzidine tetrahydrochloride development. In addition, DHE was used to stain cytosolic ROS. For BrdU labeling assays, the mice had free access to water containing BrdU (1 mg/ml) for 23 days. After sacrifice, the pancreatic sections were stained with anti‐BrdU antibody, followed by colorimetric development (BD Biosciences, San Jose, CA). Alternatively, the pancreatic sections were subjected to TUNEL assays (Chemicon, Temecula, CA). The slides were photographed and analyzed using the Axio Vision program (Carl Zeiss). For cell death, WT, *Pdia4^−/−^*, and Pdia4^tg/tg^ islets were grown in complete DMEM medium containing 3.3 (LG) and 30 mM glucose (HG) for 12 h. One aliquot of the islets were stained with PI and quantified. The other aliquot underwent immunoblotting analysis using the indicated antibodies. For confocal analysis, Min6 cells, stained with the antibody against Pdia4, p22^phox^ and/or Ndufs3, and mouse islets, stained with the antibody against Pdia4, insulin and/or glucagon, were visualized using confocal microscopy.

### Measurement of ROS and the activity of Nox and ETC

Pancreatic islets isolated from the mice were pre‐treated with 3.3 mM (LG) and 16.7 mM (HG) glucose for 30 min. The islets were then incubated with Hochest 33342 plus CM‐H_2_DCFDA or MitoGreen plus MitoSOX for additional 30 min. Alternatively, Min6 cells and those infected with lentiviral particles were grown in RPMI medium in the presence or absence of NAC (1 mM) and GHTT (10 μg/ml) for 30 min. The cells were then incubated with CellROX plus Hochest 33342 or MitoGreen plus Mitosox in the presence of 0.5 mM (LG) and 25 mM (HG) glucose for an additional 30 min. The ROS signal of the islets or cells was recorded using confocal microscopy and quantified using the Zen software. To measure serum ROS (Tsai *et al*, 2011), the sera of mouse tail veins were incubated with lucigenin and monitored using Victor 3 (Perkin Elmer). Mouse islets were pre‐treated with 3.3 mM (LG) and 16.7 mM (HG) glucose for 30 min. Membrane and mitochondrial fractions of the islets were prepared with protein extraction kits (Abcam, UK) according to the manufacturer’s protocol. The membrane fractions were incubated with lucigenin and NADPH and tested for Nox activity as published (Minkenberg & Ferber, 1984). The mitochondrial fractions were measured for the activity of ETC I to IV using MitoCheck CI, CII/III and CIV kits based on the manufacturer’s guidelines (Cayman, MI).

### Immunoblotting analysis

All the cells were lysed with RIPA buffer unless indicated otherwise. The cell lysates or fractions underwent immunoblotting analysis. Alternatively, Min6 cells, human islets, and/or mouse islets were treated with glucose at the indicated dosages or 0.4 mM palmitate for 48 h. After extensive washing, the cells or islets were either lysed with RIPA buffer or further fractionated into mitochondrial, membrane, and cytosolic fractions with Abcam protein extraction kits. For ER proteins, the cell lysates were subjected to discontinuous sucrose gradients to purify ER organelles using centrifugation, followed by ER organelle harvest and lysis.

### Interaction and domain mapping analysis of Pdia4 with Ndufs3 and p22^phox^

To characterize the *in vivo* interaction of Pdia4 with Ndufs3, p22^phox^, and Ero1, 293T cells were transfected with the plasmid encoding Flag‐tagged Pdia4 or its mutants and the plasmid expressing Myc/Flag‐tagged p22^phox^, Myc/Falg‐tagged Ndufs3 or their deletion mutants. The mitochondrial, membrane and ER fractions of the cells were fractionated and precipitated with the indicated antibodies, followed by immunoblotting analysis. The same procedure was performed to assess the *in vivo* interaction of Pdis with Ndufs3 and p22^phox^. Alternatively, 293T cells transfected with the above plasmids were pre‐treated with GHTT (10 μg/ml) for 2 h, followed by the same procedure. For *in vitro* interaction of Pdia4 with Ndufs3 and p22^phox^, His‐tagged Pdia4 (50 ng) was incubated with an equimolar amount of Gst‐TecSH3 (32 ng), Gst‐Ndufs3 (41 ng), or Gst‐p22^phox^ (33 ng), *in vitro*. After pull‐down with nickel beads, the precipitates and recombinant proteins underwent immunoblotting analysis. To test the *in vitro* interaction of Gst‐Pdia4 with His‐tagged Nox1, Nox2, Nox3, and Nox4, the recombinant proteins were incubated for 1 h, followed by an addition of Gst beads. The recombinant proteins and precipitates were subjected to immunoblotting analysis. To test the protease‐mediated regulation of Ndufs3 and p22^phox^ by Pdia4, His‐tagged Pdia4 was incubated with Gst‐Ndufs3 and ‐p22^phox^ in the presence of trypsin/EDTA (0.05%) for 10 min. The mixture then underwent immunoblotting analysis using the indicated antibodies.

### Dual‐luciferase assay

A 1,809 bp fragment of mouse Pdia4 promoter was used to replace the SV40 promoter upstream of firefly luciferase in pGL3 vector (Promega, Madison, WI) to generate pPdia4‐Luc. The plasmid pβ‐actin‐RL containing the β‐actin promoter linked to *Renilla* luciferase reporter gene was purchased from Promega. Min6 cells transfected with pPdia4‐Luc a construct composed of a Pdia4 promoter linked to a firefly luciferase gene and internal control, pβ‐actin‐RL plasmid, were incubated with DMEM medium containing 10% FBS in the presence of 3.3 mM glucose (LG) and 16.7 mM glucose (HG) for 6 h. Total lysates underwent dual‐luciferase assay. Pdia4 promoter activity is indicated in folds obtained from the ratio of firefly luciferase activity to *Renilla* luciferase activity in the lysates.

### Liquid chromatography‐mass spectroscopy (LC‐MS)

To determine Pdia4 partners related to ROS production in β‐cells, Min6 cells were lyzed with RIPA buffer. The lysates were precipitated with anti‐Pdia4 antibody and isotype antibody. The precipitates underwent LC‐MS analysis, followed by Mascot database search against the International Protein Index mouse V 3.87 database. Similarly, Min6 cells were fractionated into the cytosolic, nuclear, membrane, mitochondrial, and ER fractions as described in the Materials and Methods section. The above proteins and mouse serum underwent electrophoresis, respectively. The protein bands corresponding to Pdia4 were eluted from the sodium dodecyl sulfate gel and digested with Asp‐N endopeptidase (NEB, USA) overnight. The peptide mixture was subjected to LC‐MS/MS analysis in the PRM (parallel reaction monitoring) mode using a synthesized Pdia4 peptide (DEHATKRSRTKEEL) as a standard peptide, followed by identification of the target m/z(s) of the peptide precursor and product ions using Skyline software (MacCoss Lab, University of Washington).

### Statistics

Data from three independent experiments or more are presented as mean ± standard deviation (SD). ANOVA test and log rank were used for statistical analysis of differences between groups, and *P* (*) < 0.05; *P* (**) < 0.01 and *P* (***) < 0.001 are considered statistically significant.

## Author contributions

W‐CY conceptualized and supervised this study. T‐FK, S‐WH, S‐HH, CL‐TC, C‐SF, M‐GH, T‐YC, M‐TY, T‐NW, C‐YY, K‐CT, GY, and W‐CY designed and performed experiments, analyzed data, interpreted results, and wrote the manuscript, with input from S‐HK, C‐YH, and S‐TJ.

## Conflict of interest

The authors declare that they have no conflict of interest.

## Supporting information



Appendix

Source Data for Figure 1

Source Data for Figure 2

Source Data for Figure 3

Source Data for Figure 4

Source Data for Figure 5

Source Data for Figure 6

Source Data for Figure 8

## Data Availability

The Proteomics data have been deposited in PeptideAtlas with the accession code PASS01396 (https://db.systemsbiology.net/sbeams/cgi/PeptideAtlas/PASS_View?identifier=PASS01396). All other data supporting the findings of this study are available within the paper or as supplementary Source Data files. Any other additional data that may be of interest are available from the corresponding author upon reasonable request.

## References

[emmm201911668-bib-0001] Almeida S , Zhou L , Gao FB (2011) Progranulin, a glycoprotein deficient in frontotemporal dementia, is a novel substrate of several protein disulfide isomerase family proteins. PLoS One 6: e26454 22028881 10.1371/journal.pone.0026454 PMC3196579

[emmm201911668-bib-0002] Ardestani A , Paroni F , Azizi Z , Kaur S , Khobragade V , Yuan T , Frogne T , Tao W , Oberholzer J , Pattou F *et al* (2014) MST1 is a key regulator of beta cell apoptosis and dysfunction in diabetes. Nat Med 20: 385–397 24633305 10.1038/nm.3482 PMC3981675

[emmm201911668-bib-0003] Ardestani A , Maedler K (2016) MST1: a promising therapeutic target to restore functional beta cell mass in diabetes. Diabetologia 59: 1843–1849 27053234 10.1007/s00125-016-3892-9

[emmm201911668-bib-0004] Bindokas VP , Kuznetsov A , Sreenan S , Polonsky KS , Roe MW , Philipson LH (2003) Visualizing superoxide production in normal and diabetic rat islets of Langerhans. J Biol Chem 278: 9796–9801 12514170 10.1074/jbc.M206913200

[emmm201911668-bib-0005] Brem H , Tomic‐Canic M , Entero H , Hanflik AM , Wang VM , Fallon JT , Ehrlich HP (2007) The synergism of age and db/db genotype impairs wound healing. Exp Gerontol 42: 523–531 17275236 10.1016/j.exger.2006.11.018

[emmm201911668-bib-0006] Cerf ME (2013) Beta cell dysfunction and insulin resistance. Front Endocrinol 4: 37 10.3389/fendo.2013.00037 PMC3608918 23542897

[emmm201911668-bib-0007] Chang YC , Chuang LM (2010) The role of oxidative stress in the pathogenesis of type 2 diabetes: from molecular mechanism to clinical implication. Am J Transl Res 2: 316–331 20589170 PMC2892404

[emmm201911668-bib-0008] Chang CL , Liu HY , Kuo TF , Hsu YJ , Shen MY , Pan CY , Yang WC (2013) Antidiabetic effect and mode of action of cytopiloyne. Evid Based Complement Alternat Med 2013: 685642 23573144 10.1155/2013/685642 PMC3610345

[emmm201911668-bib-0009] Defronzo RA (2009) Banting Lecture. From the triumvirate to the ominous octet: a new paradigm for the treatment of type 2 diabetes mellitus. Diabetes 58: 773–795 19336687 10.2337/db09-9028 PMC2661582

[emmm201911668-bib-0010] Donath MY , Halban PA (2004) Decreased beta‐cell mass in diabetes: significance, mechanisms and therapeutic implications. Diabetologia 47: 581–589 14767595 10.1007/s00125-004-1336-4

[emmm201911668-bib-0011] Dos Santos JM , Tewari S , Mendes RH (2019) The role of oxidative stress in the development of diabetes mellitus and its complications. J Diabetes Res 2019: 1–3 10.1155/2019/4189813 PMC6525877 31192263

[emmm201911668-bib-0012] Efrat S (2019) Beta‐cell dedifferentiation in type 2 diabetes: concise review. Stem Cells 37: 1267–1272 31298804 10.1002/stem.3059

[emmm201911668-bib-0013] Ellerman DA , Myles DG , Primakoff P (2006) A role for sperm surface protein disulfide isomerase activity in gamete fusion: evidence for the participation of ERp57. Dev Cell 10: 831–837 16740484 10.1016/j.devcel.2006.03.011

[emmm201911668-bib-0014] Evans JL , Maddux BA , Goldfine ID (2005) The molecular basis for oxidative stress‐induced insulin resistance. Antioxid Redox Signal 7: 1040–1052 15998259 10.1089/ars.2005.7.1040

[emmm201911668-bib-0015] Galligan JJ , Petersen DR (2012) The human protein disulfide isomerase gene family. Hum Genomics 6: 6 23245351 10.1186/1479-7364-6-6 PMC3500226

[emmm201911668-bib-0016] Gao XY , Yan D , Zhao YA , Tao H , Zhou YS (2015) Moderate calorie restriction to achieve normal weight reverses beta‐cell dysfunction in diet‐induced obese mice: involvement of autophagy. Nutr Metab 12: 34–43 10.1186/s12986-015-0028-z PMC4595003 26445593

[emmm201911668-bib-0017] Garbi N , Tanaka S , Momburg F , Hammerling GJ (2006) Impaired assembly of the major histocompatibility complex class I peptide‐loading complex in mice deficient in the oxidoreductase ERp57. Nat Immunol 7: 93–102 16311600 10.1038/ni1288

[emmm201911668-bib-0018] Gerber PA , Rutter GA (2017) The role of oxidative stress and hypoxia in pancreatic beta‐cell dysfunction in diabetes mellitus. Antioxid Redox Signal 26: 501–518 27225690 10.1089/ars.2016.6755 PMC5372767

[emmm201911668-bib-0019] Goplen D , Wang J , Enger PO , Tysnes BB , Terzis AJ , Laerum OD , Bjerkvig R (2006) Protein disulfide isomerase expression is related to the invasive properties of malignant glioma. Cancer Res 66: 9895–9902 17047051 10.1158/0008-5472.CAN-05-4589

[emmm201911668-bib-0020] Harrity T , Farrelly D , Tieman A , Chu C , Kunselman L , Gu L , Ponticiello R , Cap M , Qu F , Shao C *et al* (2006) Muraglitazar, a novel dual (alpha/gamma) peroxisome proliferator‐activated receptor activator, improves diabetes and other metabolic abnormalities and preserves beta‐cell function in db/db mice. Diabetes 55: 240–248 16380499

[emmm201911668-bib-0021] Herbert TP , Laybutt DR (2016) A reevaluation of the role of the unfolded protein response in islet dysfunction: maladaptation or a failure to adapt? Diabetes 65: 1472–1480 27222391 10.2337/db15-1633

[emmm201911668-bib-0022] Hinder LM , O'Brien PD , Hayes JM , Backus C , Solway AP , Sims‐Robinson C , Feldman EL (2017) Dietary reversal of neuropathy in a murine model of prediabetes and metabolic syndrome. Dis Model Mech 10: 717–725 28381495 10.1242/dmm.028530 PMC5483005

[emmm201911668-bib-0023] Huang X , Sun M , Li D , Liu J , Guo H , Dong Y , Jiang L , Pan QI , Man Y , Wang S *et al* (2011) Augmented NADPH oxidase activity and p22phox expression in monocytes underlie oxidative stress of patients with type 2 diabetes mellitus. Diabetes Res Clin Pract 91: 371–380 21237524 10.1016/j.diabres.2010.12.026

[emmm201911668-bib-0024] Kim‐Muller JY , Fan J , Kim YJ , Lee SA , Ishida E , Blaner WS , Accili D (2016) Aldehyde dehydrogenase 1a3 defines a subset of failing pancreatic beta cells in diabetic mice. Nat Commun 7: 12631 27572106 10.1038/ncomms12631 PMC5013715

[emmm201911668-bib-0025] Kuo TF , Chen TY , Jiang ST , Chen KW , Chiang YM , Hsu YJ , Liu YJ , Chen HM , Yokoyama KK , Tsai KC *et al* (2017) Identification of protein disulfide isomerase a4 as a novel therapeutic target of cancer. Oncogene 36: 5484–5496 28534513 10.1038/onc.2017.156

[emmm201911668-bib-0026] Leahy JL , Hirsch IB , Peterson KA , Schneider D (2010) Targeting beta‐cell function early in the course of therapy for type 2 diabetes mellitus. J Clin Endocrinol Metab 95: 4206–4216 20739389 10.1210/jc.2010-0668

[emmm201911668-bib-0027] Lei XG , Vatamaniuk MZ (2011) Two tales of antioxidant enzymes on beta cells and diabetes. Antioxid Redox Signal 14: 489–503 20618069 10.1089/ars.2010.3416 PMC3026656

[emmm201911668-bib-0028] Lenzen S , Drinkgern J , Tiedge M (1996) Low antioxidant enzyme gene expression in pancreatic islets compared with various other mouse tissues. Free Rad Biol Med 20: 463–466 8720919 10.1016/0891-5849(96)02051-5

[emmm201911668-bib-0029] Leung KK , Leung PS (2008) Effects of hyperglycemia on angiotensin II receptor type 1 expression and insulin secretion in an INS‐1E pancreatic beta‐cell line. JOP 9: 290–299 18469441

[emmm201911668-bib-0030] Li XA , Lee AS (1991) Competitive inhibition of a set of endoplasmic reticulum protein genes (GRP78, GRP94, and ERp72) retards cell growth and lowers viability after ionophore treatment. Mol Cell Biol 11: 3446–3453 2046663 10.1128/mcb.11.7.3446-3453.1991 PMC361074

[emmm201911668-bib-0031] Maattanen P , Kozlov G , Gehring K , Thomas DY (2006) ERp57 and PDI: multifunctional protein disulfide isomerases with similar domain architectures but differing substrate‐partner associations. Biochem Cell Biol 84: 881–889 17215875 10.1139/o06-186

[emmm201911668-bib-0032] Manukyan D , von Bruehl ML , Massberg S , Engelmann B (2008) Protein disulfide isomerase as a trigger for tissue factor‐dependent fibrin generation. Thromb Res 122(Suppl 1): S19–S22 10.1016/S0049-3848(08)70013-6 18691493

[emmm201911668-bib-0033] Mao HZ , Roussos EI , Peterfy M (2006) Genetic analysis of the diabetes‐prone C57BLKS/J mouse strain reveals genetic contribution from multiple strains. Bioph. Biochem. Acta 1762: 440–446 10.1016/j.bbadis.2006.01.002 16481151

[emmm201911668-bib-0034] Matthews DR , Cull CA , Stratton IM , Holman RR , Turner RC (1998) UKPDS 26: Sulphonylurea failure in non‐insulin‐dependent diabetic patients over six years. UK Prospective Diabetes Study (UKPDS) Group. Diabet Med 15: 297–303 9585394 10.1002/(SICI)1096-9136(199804)15:4<297::AID-DIA572>3.0.CO;2-W

[emmm201911668-bib-0035] Minkenberg I , Ferber E (1984) Lucigenin‐dependent chemiluminescence as a new assay for NAD(P)H‐oxidase activity in particulate fractions of human polymorphonuclear leukocytes. J Immunol Methods 71: 61–67 6725961 10.1016/0022-1759(84)90206-0

[emmm201911668-bib-0036] Naguleswaran A , Alaeddine F , Guionaud C , Vonlaufen N , Sonda S , Jenoe P , Mevissen M , Hemphill A (2005) Neospora caninum protein disulfide isomerase is involved in tachyzoite‐host cell interaction. Int J Parasitol 35: 1459–1472 16129440 10.1016/j.ijpara.2005.06.006

[emmm201911668-bib-0037] Nakamura U , Iwase M , Uchizono Y , Sonoki K , Sasaki N , Imoto H , Goto D , Iida M (2006) Rapid intracellular acidification and cell death by H2O2 and alloxan in pancreatic beta cells. Free Rad Biol Med 40: 2047–2055 16716905 10.1016/j.freeradbiomed.2006.01.038

[emmm201911668-bib-0038] Newsholme P , Rebelato E , Abdulkader F , Krause M , Carpinelli A , Curi R (2012) Reactive oxygen and nitrogen species generation, antioxidant defenses, and beta‐cell function: a critical role for amino acids. J Endocrinol 214: 11–20 22547566 10.1530/JOE-12-0072

[emmm201911668-bib-0039] Ni M , Lee AS (2007) ER chaperones in mammalian development and human diseases. Febs Lett 581: 3641–3651 17481612 10.1016/j.febslet.2007.04.045 PMC2040386

[emmm201911668-bib-0040] Norgaard P , Westphal V , Tachibana C , Alsoe L , Holst B , Winther JR (2001) Functional differences in yeast protein disulfide isomerases. J Cell Biol 152: 553–562 11157982 10.1083/jcb.152.3.553 PMC2195995

[emmm201911668-bib-0041] Ou W , Silver J (2006) Role of protein disulfide isomerase and other thiol‐reactive proteins in HIV‐1 envelope protein‐mediated fusion. Virology 350: 406–417 16507315 10.1016/j.virol.2006.01.041

[emmm201911668-bib-0042] Parker R , Phan T , Baumeister P , Roy B , Cheriyath V , Roy AL , Lee AS (2001) Identification of TFII‐I as the endoplasmic reticulum stress response element binding factor ERSF: its autoregulation by stress and interaction with ATF6. Mol Cell Biol 21: 3220–3233 11287625 10.1128/MCB.21.9.3220-3233.2001 PMC86961

[emmm201911668-bib-0043] Pawar H , Kashyap MK , Sahasrabuddhe NA , Renuse S , Harsha HC , Kumar P , Sharma J , Kandasamy K , Marimuthu A , Nair B *et al* (2011) Quantitative tissue proteomics of esophageal squamous cell carcinoma for novel biomarker discovery. Cancer Biol Ther 12: 510–522 21743296 10.4161/cbt.12.6.16833 PMC3218592

[emmm201911668-bib-0044] Quan W , Lim YM , Lee MS (2012) Role of autophagy in diabetes and endoplasmic reticulum stress of pancreatic beta‐cells. Exp Mol Med 44: 81–88 22257883 10.3858/emm.2012.44.2.030 PMC3296816

[emmm201911668-bib-0045] Robertson RP (2004) Chronic oxidative stress as a central mechanism for glucose toxicity in pancreatic islet beta cells in diabetes. J Biol Chem 279: 42351–42354 15258147 10.1074/jbc.R400019200

[emmm201911668-bib-0046] Robertson RP , Harmon J , Tran PO , Poitout V (2004) Beta‐cell glucose toxicity, lipotoxicity, and chronic oxidative stress in type 2 diabetes. Diabetes 53(Suppl 1): S119–S124 14749276 10.2337/diabetes.53.2007.s119

[emmm201911668-bib-0047] Robertson RP , Harmon JS (2007) Pancreatic islet beta‐cell and oxidative stress: the importance of glutathione peroxidase. Febs Lett 581: 3743–3748 17433304 10.1016/j.febslet.2007.03.087 PMC2762945

[emmm201911668-bib-0048] Schultz‐Norton JR , McDonald WH , Yates JR , Nardulli AM (2006) Protein disulfide isomerase serves as a molecular chaperone to maintain estrogen receptor alpha structure and function. Mol Endocrinol (Baltimore, MD) 20: 1982–1995 10.1210/me.2006-0006 16690750

[emmm201911668-bib-0049] Severino A , Campioni M , Straino S , Salloum FN , Schmidt N , Herbrand U , Frede S , Toietta G , Di Rocco G , Bussani R *et al* (2007) Identification of protein disulfide isomerase as a cardiomyocyte survival factor in ischemic cardiomyopathy. J Am Coll Cardiol 50: 1029–1037 17825711 10.1016/j.jacc.2007.06.006

[emmm201911668-bib-0050] Tangvarasittichai S (2015) Oxidative stress, insulin resistance, dyslipidemia and type 2 diabetes mellitus. World J Diabetes 6: 456–480 25897356 10.4239/wjd.v6.i3.456 PMC4398902

[emmm201911668-bib-0051] Tiedge M , Lortz S , Drinkgern J , Lenzen S (1997) Relation between antioxidant enzyme gene expression and antioxidative defense status of insulin‐producing cells. Diabetes 46: 1733–1742 9356019 10.2337/diab.46.11.1733

[emmm201911668-bib-0052] Trachootham D , Alexandre J , Huang P (2009) Targeting cancer cells by ROS‐mediated mechanisms: a radical therapeutic approach? Nat Rev Drug Discov 8: 579–591 19478820 10.1038/nrd2803

[emmm201911668-bib-0053] Tsai P‐Y , Ka S‐M , Chang J‐M , Chen H‐C , Shui H‐A , Li C‐Y , Hua K‐F , Chang W‐L , Huang J‐J , Yang S‐S *et al* (2011) Epigallocatechin‐3‐gallate prevents lupus nephritis development in mice via enhancing the Nrf2 antioxidant pathway and inhibiting NLRP3 inflammasome activation. Free Rad Biol Med 51: 744–754 21641991 10.1016/j.freeradbiomed.2011.05.016

[emmm201911668-bib-0054] Turano C , Coppari S , Altieri F , Ferraro A (2002) Proteins of the PDI family: unpredicted non‐ER locations and functions. J Cell Physiol 193: 154–163 12384992 10.1002/jcp.10172

[emmm201911668-bib-0055] Weaver JR , Grzesik W , Taylor‐Fishwick DA (2015) Inhibition of NADPH oxidase‐1 preserves beta cell function. Diabetologia 58: 113–121 25277953 10.1007/s00125-014-3398-2

[emmm201911668-bib-0056] Winter AD , McCormack G , Page AP (2007) Protein disulfide isomerase activity is essential for viability and extracellular matrix formation in the nematode *Caenorhabditis elegans* . Dev Biol 308: 449–461 17586485 10.1016/j.ydbio.2007.05.041

[emmm201911668-bib-0057] Wu J , Luo X , Thangthaeng N , Sumien N , Chen Z , Rutledge MA , Jing S , Forster MJ , Yan LJ (2017) Pancreatic mitochondrial complex I exhibits aberrant hyperactivity in diabetes. Biochem Biophys Rep 11: 119–129 28868496 10.1016/j.bbrep.2017.07.007 PMC5580358

[emmm201911668-bib-0058] Xiong Y , Manevich Y , Tew KD , Townsend DM (2012) S‐glutathionylation of protein disulfide isomerase regulates estrogen receptor alpha stability and function. Intl Cell Biol 2012: 273549 10.1155/2012/273549 PMC3359683 22654912

[emmm201911668-bib-0059] Yagi N , Yokono K , Amano K , Nagata M , Tsukamoto K , Hasegawa Y , Yoneda R , Okamoto N , Moriyama H , Miki M *et al* (1995) Expression of intercellular adhesion molecule 1 on pancreatic beta‐cells accelerates beta‐cell destruction by cytotoxic T‐cells in murine autoimmune diabetes. Diabetes 44: 744–752 7789642 10.2337/diab.44.7.744

[emmm201911668-bib-0060] Yang J‐S , Lu C‐C , Kuo S‐C , Hsu Y‐M , Tsai S‐C , Chen S‐Y , Chen Y‐T , Lin Y‐J , Huang Y‐C , Chen C‐J *et al* (2017) Autophagy and its link to type II diabetes mellitus. BioMedicine 7: 1 28612706 10.1051/bmdcn/2017070201 PMC5479440

