# Supplementary material for: Pdia4 regulates β‐cell pathogenesis in diabetes: molecular mechanism and targeted therapy
Source: EMBO Mol Med. 2021 Sep 20;13(10):e11668. doi: 10.15252/emmm.201911668 (PMC8495450; doi:10.15252/emmm.201911668)
Supplement: Supplementary file 1 — Appendix [file EMMM-13-e11668-s009.pdf]

Appendix

Content:

Appendix Figure S1. Expression pattern of Pdis in Min6 cells and mouse islets.

Appendix Figure S2. Pdia4 deficiency lowers blood glucose, improves GTT and β-cell function, and alleviates diabetes development in *Pdia4*$^{-/-}$*Lepr*$^{db/db}$ mice.

Appendix Figure S3. Diabetes development in aged *Pdia4*$^{-/-}$*Lepr*$^{db/db}$ versus *Lepr*$^{db/db}$ mice.

Appendix Figure S4. Diabetes development, islet structure, β-cell function, serum ROS and serum insulin in islet-specific Pdia4 knockout mice.

Appendix Figure S5. Diabetes development in high fat diet-induced WT, *Pdia4*$^{-/-}$, and Pdia4$^{tg/tg}$ B6 mice.

Appendix Figure S6. Pdia4 overexpression accelerates diabetes, impairs β-cell function, and deteriorates GTT in Pdia4$^{tg/tg}$*Lepr*$^{db/db}$ mice.

Appendix Figure S7. Interaction and function of Pdia4 and/or other Pdis in p22^{phox} and Ndufs3 pathways.

Appendix Figure S8. Effect of GHTT on biochemical parameters in the blood of *Lepr*$^{db/db}$ mice.

Appendix Figure S9. Effect of Pdia4 on the markers of cell death, dedifferentiation and differentiation in β cells.

Appendix Table S1. List of RNAi sequences

Appendix Table S2. List of cDNA sequences

Appendix Table S3. Antibodies used in this study

Appendix Table S4. PCR primers used in this study

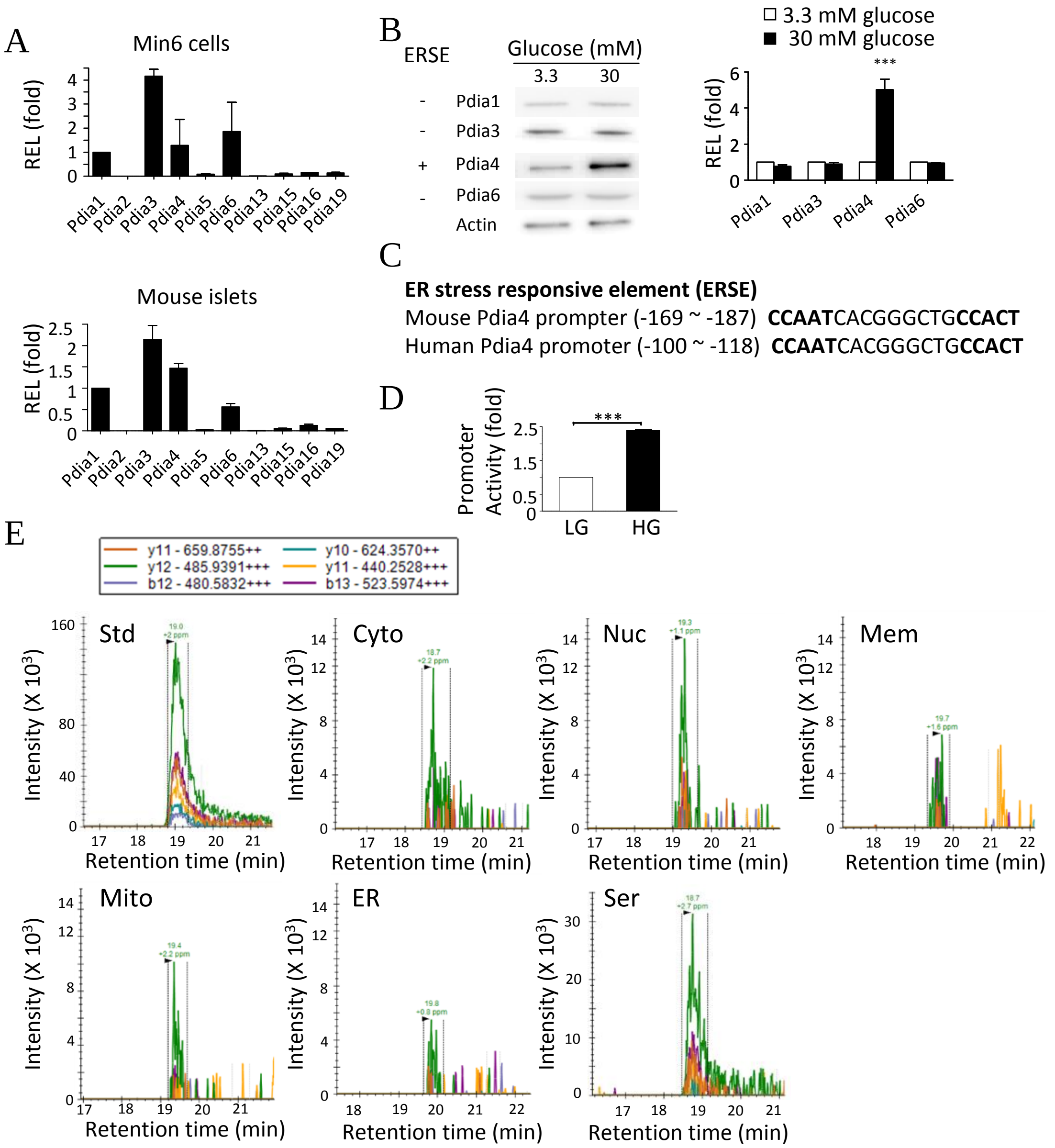

Appendix Figure S1. Expression pattern of Pdis in Min6 cells and mouse islets. (A) Total RNA of Min6 cells (top) and mouse islets (bottom) was isolated and converted into the first strand of cDNA. The cDNA samples were used as templates for real-time PCR. The relative expression level (REL) of typical Pdis versus atypical Pdis was quantified in relation to the level of actin. (B) Protein level of Pdia1, Pdia3, Pdia4 and Pdia6 in Min6 cells grown in the presence of glucose at 3.3 mM (LG) and 30 mM (HG) was measured using immunoblotting analysis. Their induction fold was determined. (C) Comparison of the ER stress responsive element (ERSE) located in the promoter region of human and mouse *Pdia4* genes. (D) Min6 cells transfected with pPdia4-Luc and pβ-actin-RL plasmids were incubated with 3.3 mM (LG) and 16.7 mM glucose (HG) for 6 h. Total lysates underwent dual luciferase assay. Pdia4 promoter activity is indicated in folds obtained from the ratio of firefly luciferase activity to *Renilla* luciferase activity in the lysates. (E) C-terminal peptide (DEHATKRSRTKEEL) of Pdia4 was identified in the cytosolic (Cyto), nuclear (Nuc), membrane (Mem), mitochondrial (Mito), and ER fractions of Min6 cells, mouse serum (Ser) and a standard peptide (Std) using LC-MS analysis. Data are representative of 3 independent experiments.

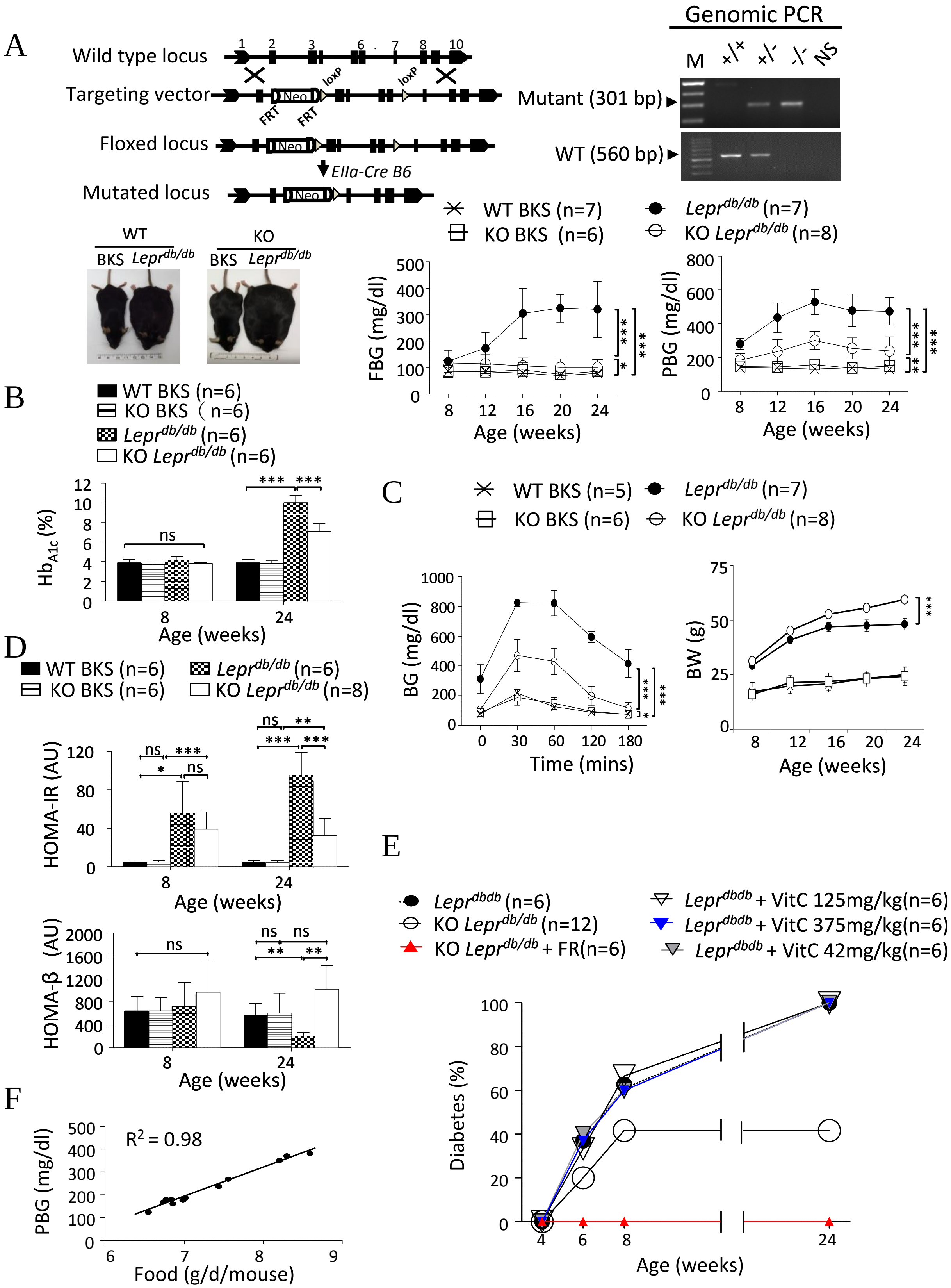

Appendix Figure S2. Pdia4 deficiency lowers blood glucose, improves GTT and β-cell function, and alleviates diabetes development in *Pdia4*$^{-/-}$*Lepr*$^{db/db}$ mice. (A) Construction,

genotyping and characterization of conventional Pdia4 knockout mice. The knockout targeting vector, loci of wild-type (WT), floxed and mutated *Pdia4* gene, FRT site (▯), loxP site (▷) and *Neo* gene are indicated (left). *Pdia4*$^{-/-}$ B6 mice were bred into BKS background. *Pdia4*$^{-/-}$ BKS mice were mated to generate WT, *Pdia4*$^{+/-}$ and *Pdia4*$^{-/-}$ BKS mice, followed by PCR genotyping (right). WT and *Pdia4*$^{-/-}$ mice on BKS and *Lepr*$^{db/db}$ backgrounds were used and photographed. FBG and PBG of WT and *Pdia4*$^{-/-}$ (KO) mice on BKS and *Lepr*$^{db/db}$ backgrounds were measured using a glucometer at the indicated ages. (B) Hb_{A1C} of the mice from (A) was measured. (C-D) 24-week-old WT and *Pdia4*$^{-/-}$ (KO) mice on BKS and *Lepr*$^{db/db}$ backgrounds from (A) were measured for GTT (left, C), body weight (right, C) and HOMA-IR (top, D) and HOMA-β (bottom, D). (E) *Lepr*$^{db/db}$ and *Pdia4*$^{-/-}$*Lepr*$^{db/db}$ mice had free access to a standard diet and a diet containing vitamin C (VitC) at the indicated doses and food restriction (FR, 6.8 g/day/mouse) from 4 to 24 weeks. Their PBG and diabetes incidence were analyzed. (F) Regression analysis of PBG and food intake in *Pdia4*$^{-/-}$*Lepr*$^{db/db}$ mice as (A) was performed. The number of mice (n) is indicated in parentheses.

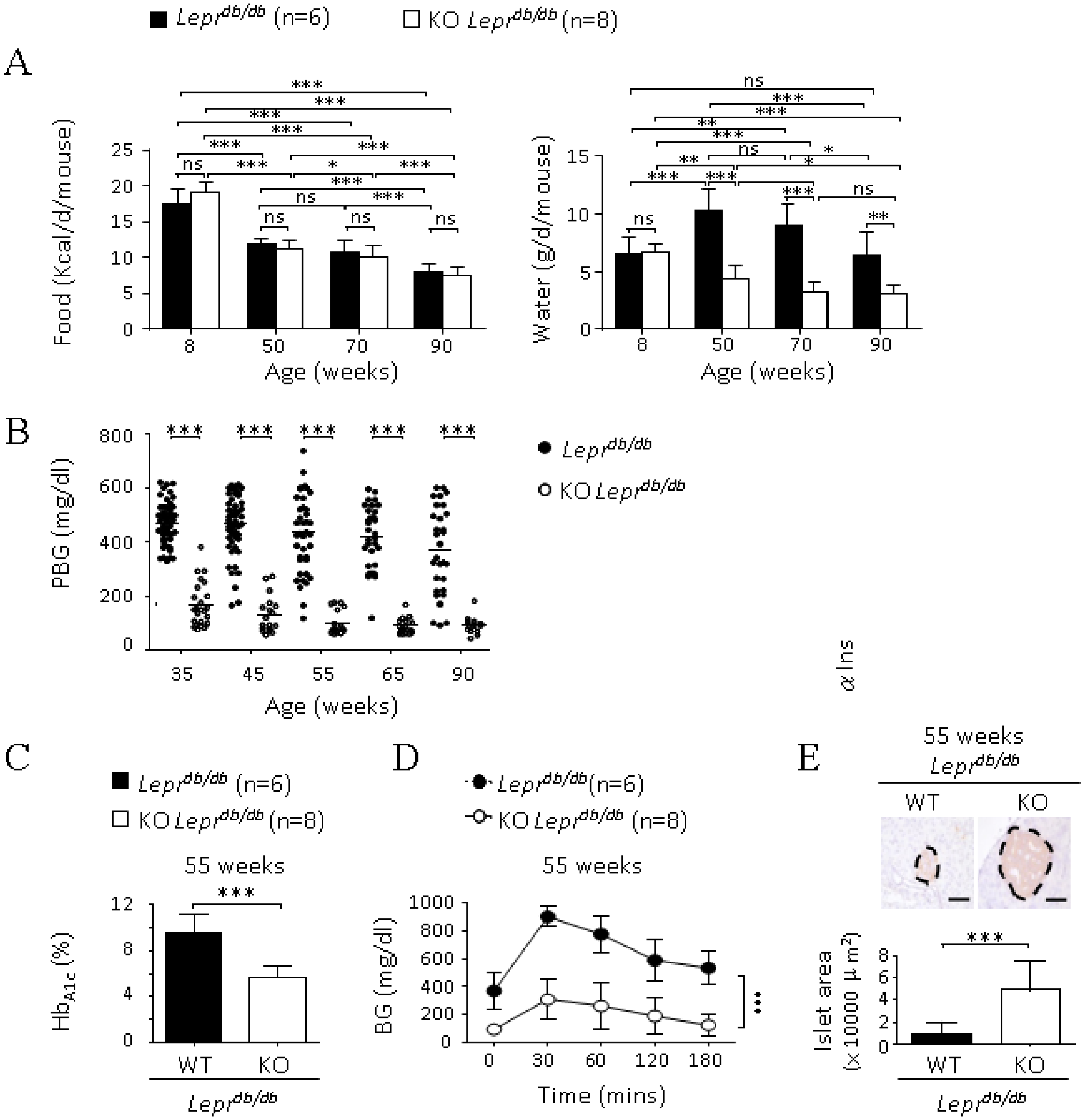

Appendix Figure S3. Diabetes development in aged *Pdia4*$^{-/-}$*Lepr*$^{db/db}$ versus *Lepr*$^{db/db}$ mice. (A-B) *Lepr*$^{db/db}$ (WT) and *Pdia4*$^{-/-}$*Lepr*$^{db/db}$ (KO) mice from Appendix Figure S2 were monitored for their consumption of food and water (A) and PBG (B), from birth to 90 weeks. (C-E) The Hb_{A1C} (C), GTT (D), and islet structure (E), of 55-week-old mice were determined. The number of mice (n) is indicated in parentheses.

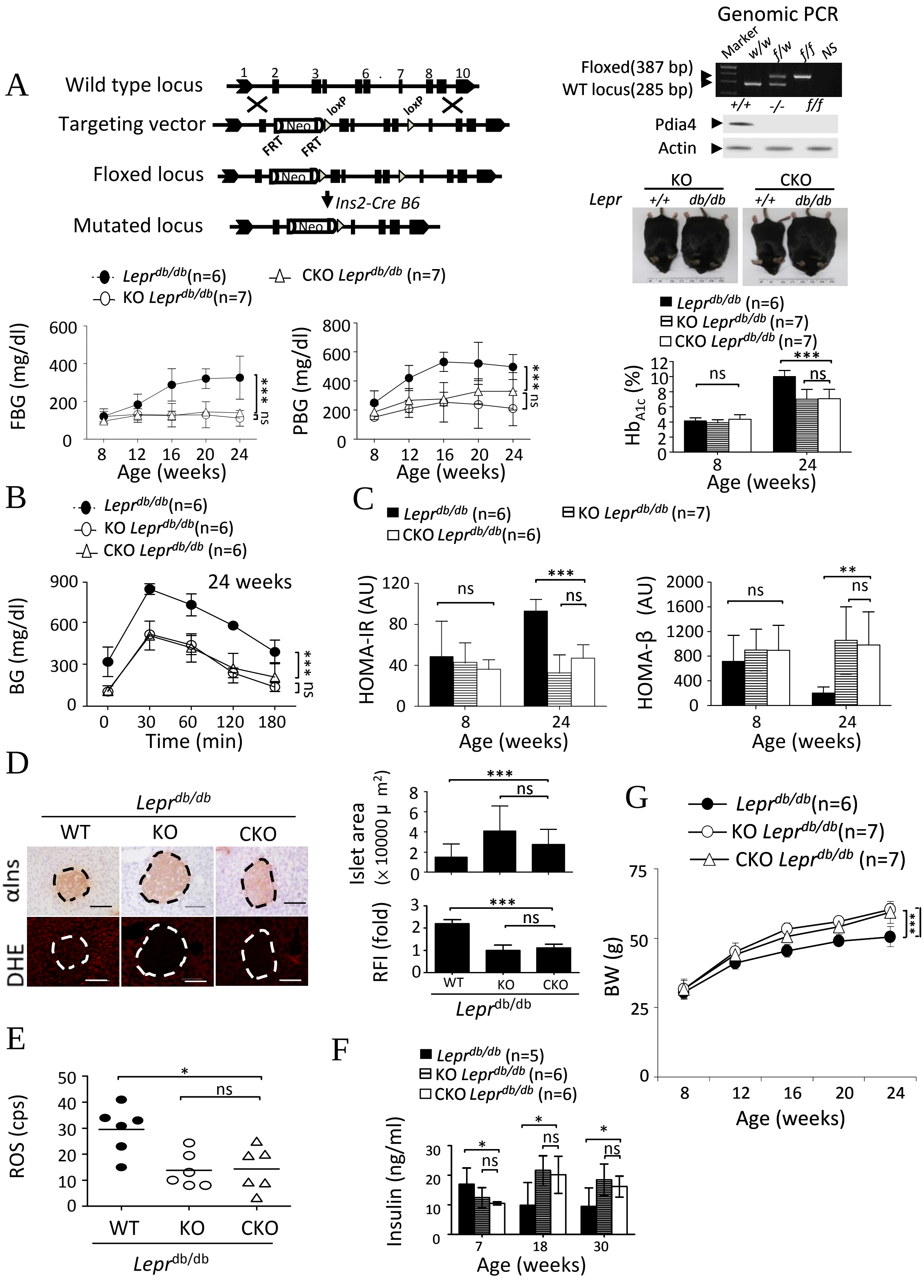

Appendix Figure S4. Diabetes development, islet structure, β-cell function, serum ROS and serum insulin in islet-specific Pdia4 knockout mice. (A) Construction, genotyping and

characterization of conditional Pdia4 knockout mice. Conditional knockout mice were generated based on the following strategy. The *Pdia4*$^{f/+}$ B6 mice from Appendix Figure S2A underwent sibling mating to generate *Pdia4*$^{f/f}$ B6 mice. *Pdia4*$^{f/f}$ B6 mice were crossed with Ins2-Cre deleter mice to obtain the *Pdia4*$^{f/f}$Cre$^{tg/tg}$ B6 mice. Genotyping was performed by PCR and immunoblotting analysis. In addition, the mice were crossed with BKS mice and, then, B6.BKS(D)-*Lepr*^{db}/J mice to obtain *Pdia4*$^{f/f}$*Lepr*$^{db/db}$Cre$^{tg/0}$ (CKO *Lepr*$^{db/db}$) mice for diabetes study. FBG, PBG and Hb_{A1C} of islet-specific knockout (CKO *Lepr*$^{db/db}$) mice versus conventional knockout mice (KO *Lepr*$^{db/db}$) and *Lepr*$^{db/db}$ mice were determined. (B-C) GTT (B) and HOMA indices (C) of the mice (A) were measured. (D) Pancreata of the mice (B) were stained with anti-insulin (αIns) antibody and DHE. Islet size (μm^2) and relative fluorescence intensity (RFI) were quantified. Scale bar: 100 μm. (E-F) Serum ROS (E) and insulin (F) of the mice from (B) were measured. (G) Body weight of the mice (A) was monitored at the indicated age. The number of mice (n) is indicated in parentheses.

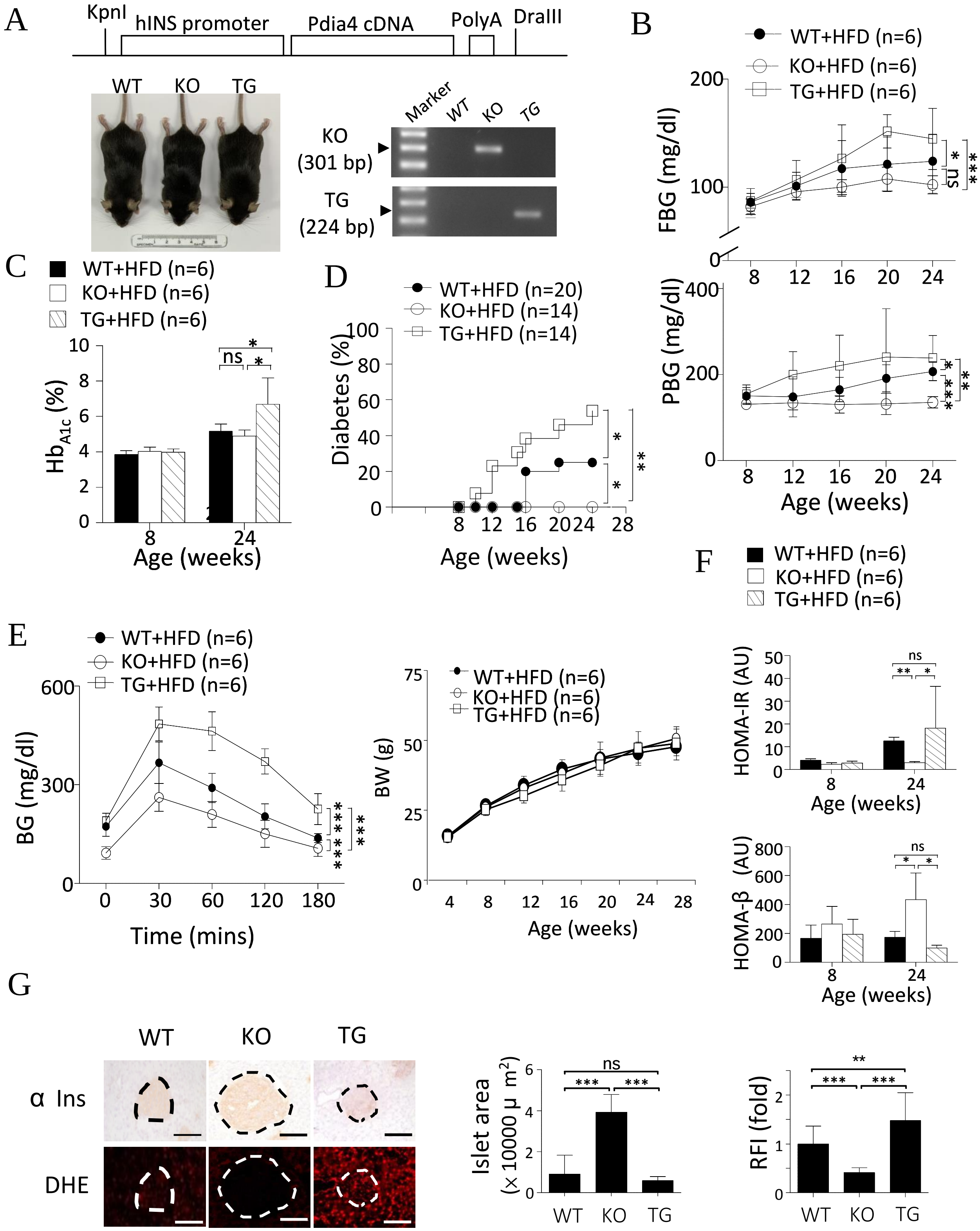

Appendix Figure S5. Diabetes development in high fat diet-induced WT, *Pdia4*$^{-/-}$, and Pdia4$^{tg/tg}$ B6 mice. (A) A schematic diagram illustrating the Pdia4 transgenic construct composed of the human insulin (hINS) promoter linked to a human Pdia4 cDNA. A linearized KpnI/DraIII fragment from this

construct was microinjected into the pronuclei of B6 fertilized eggs to transgenic lines. These transgenic lines were characterized using Southern blots. One line, Pdia4$^{tg/tg}$ mice, was selected and genotyped with PCR. (B-F) WT, *Pdia4*$^{-/-}$ (KO) and Pdia4$^{tg/tg}$ (TG) B6 mice had free access to a high fat diet (60% of fat) from 4 to 28 weeks of age. Their FBG (top, B), PBG (bottom, B), Hb_{A1c} (C), diabetes incidence (D), GTT (left, E), body weight (right, E), HOMA-IR (top, F) and HOMA-β (bottom, F) were measured at the indicated ages. (G) Pancreata of the mice (B) were stained with anti-insulin (αIns) antibody and DHE (left). Islet size (μm^2) and RFI were quantified (right). Scale bar: 100 μm. The number of mice (n) is indicated in parentheses.

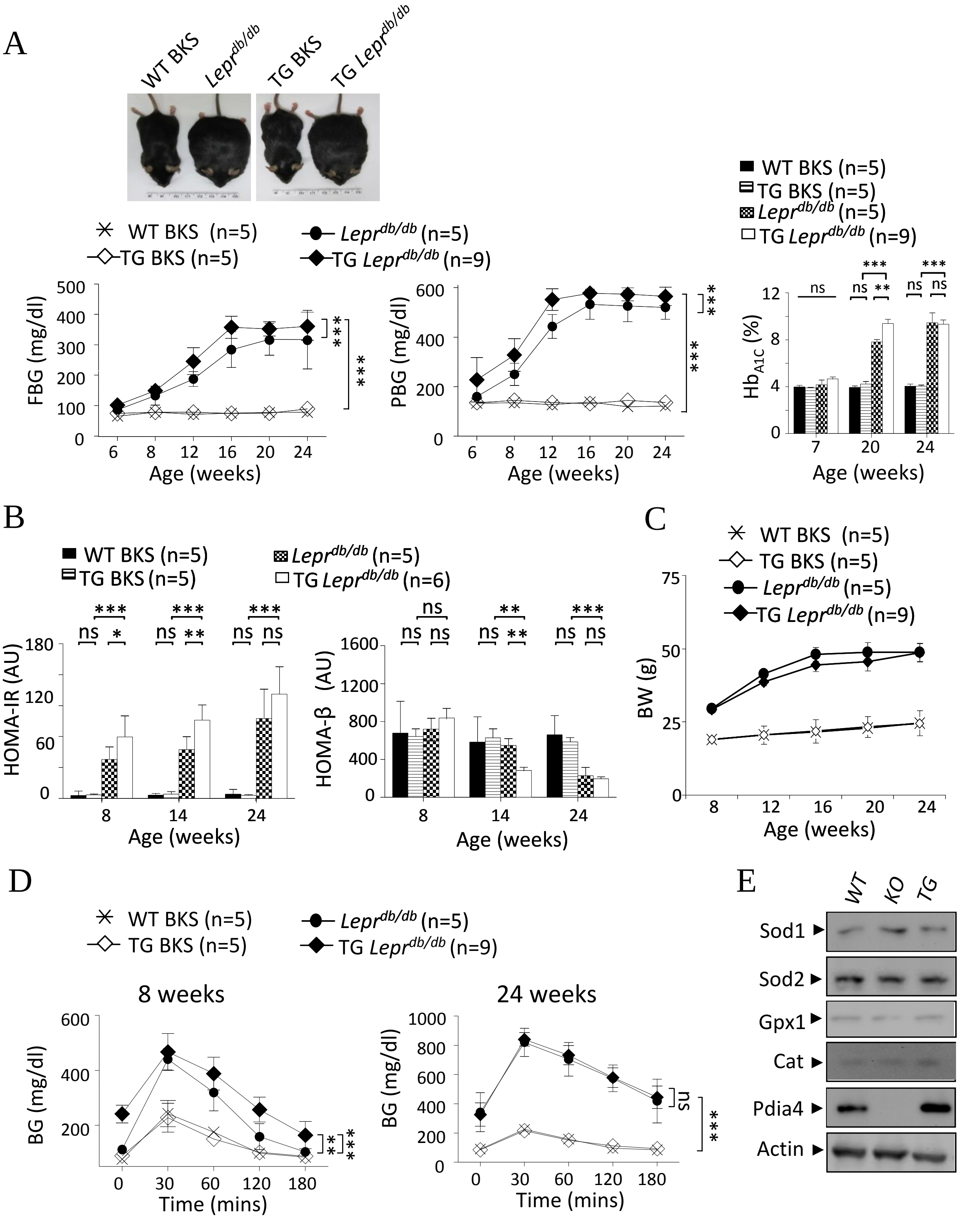

Appendix Figure S6. Pdia4 overexpression accelerates diabetes, impairs β-cell function, and deteriorates GTT in Pdia4$^{tg/tg}$*Lepr*$^{db/db}$ mice. (A) Pdia4$^{tg/tg}$ B6 (TG) mice (Appendix Figure S5A) were bred with BKS mice and, then, B6.BKS(D)-*Lepr*^{db}/J mice to generate Pdia4$^{tg/tg}$*Lepr*$^{db/db}$ (TG *Lepr*$^{db/db}$) mice. FBG, PBG, and Hb_{A1C} of wild-type (WT) and Pdia4$^{tg/tg}$ (TG) mice on BKS and

Lepr$^{db/db}$ backgrounds were measured at the indicated ages. (B-D) HOMA indices (B), body weight (C), and GTT (D) of the mice (A) were performed at the indicated ages. (E) Expression level of anti-oxidant proteins in WT, *Pdia4*$^{-/-}$ (KO) and Pdia4$^{tg/tg}$ (TG) BKS mice. Total lysates of WT, *Pdia4*$^{-/-}$ and Pdia4$^{tg/tg}$ BKS islets were subjected to immunoblotting analysis using the indicated antibodies. The number of mice (n) is indicated in parentheses.

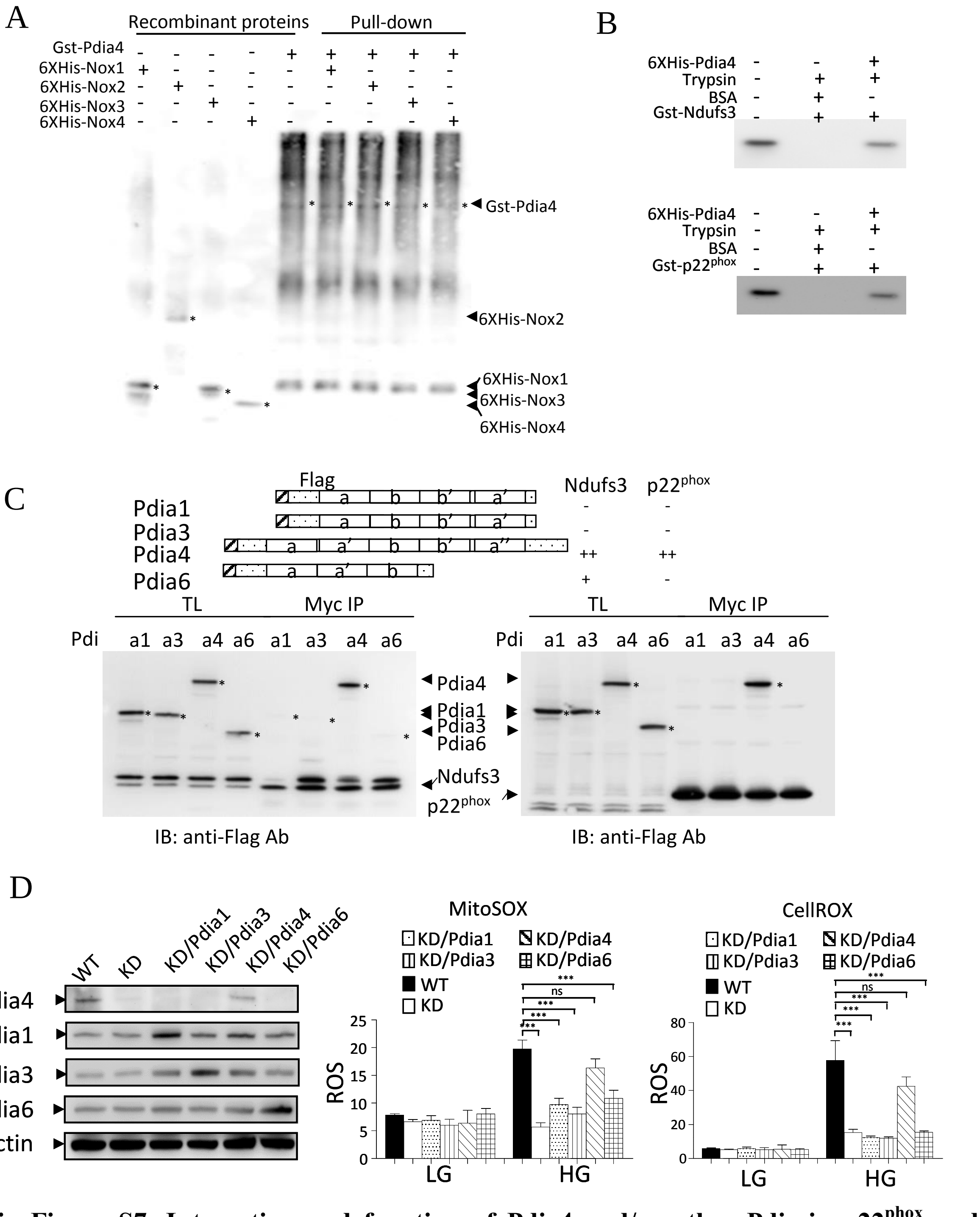

Appendix Figure S7. Interaction and function of Pdia4 and/or other Pdis in p22^{phox} and Ndufs3 pathways. (A) Gst-Pdia4 was incubated with an equimolar ratio of the recombinant proteins, His-tagged Nox1, Nox2, Nox3, and Nox4 *in vitro*. After pull-down with Gst beads, the precipitates and recombinant proteins underwent SDS-PAGE and immunoblotting analysis using anti-Gst and anti-His antibodies. (B) His-tagged Pdia4 and an irrelevant protein, bovine serum albumin (BSA), were incubated with an equimolar Gst-Ndufs3 (top) or Gst-p22^{phox} (bottom) in the presence of trypsin, followed by immunoblotting analysis using the indicated antibodies. (C) The construct encoding Flag-tagged Pdia1, Pdia3, Pdia4 or Pdia6 and that expressing pMyc/Falg-tagged Ndufs3 (left) or p22^{phox} (right) were co-transfected into 293T cells. After lysis, total lysates

were precipitated with anti-Myc antibody plus protein G beads. The lysates (TL) and immunoprecipitates (IP) underwent immunoblotting analysis using anti-Flag antibody. (D) Parental Min6 cells (WT) or Min6 KD cells with Pdia4 knockdown were infected with lentiviral particles expressing the indicated Pdis. The cells were examined for the protein level of Pdis (left). The level of mitochondrial (middle) and cytosolic ROS (right) in the cells treated with low glucose (LG) and high glucose (HG) was measured. Data are representative of 3 independent experiments.

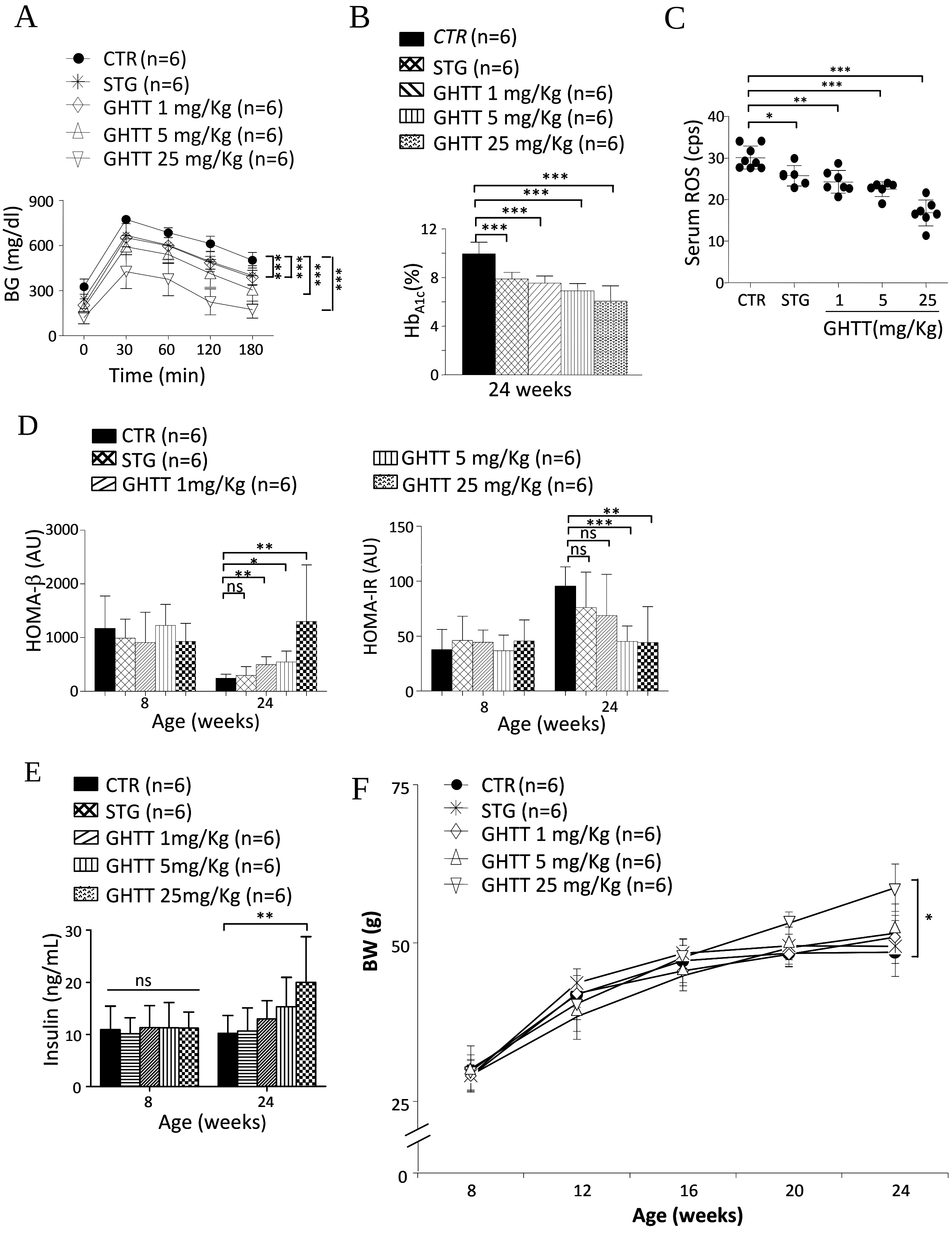

Appendix Figure S8. Effect of GHTT on biochemical parameters in the blood of *Lepr*$^{db/db}$ mice. The mice from Figure 8E were measured for their GTT (A), Hb_{A1c} (B), serum ROS (C),

HOMA-β (left, D), HOMA-IR (right, D), serum insulin (E), and body weight (F). The number of mice (n) is indicated in parentheses.

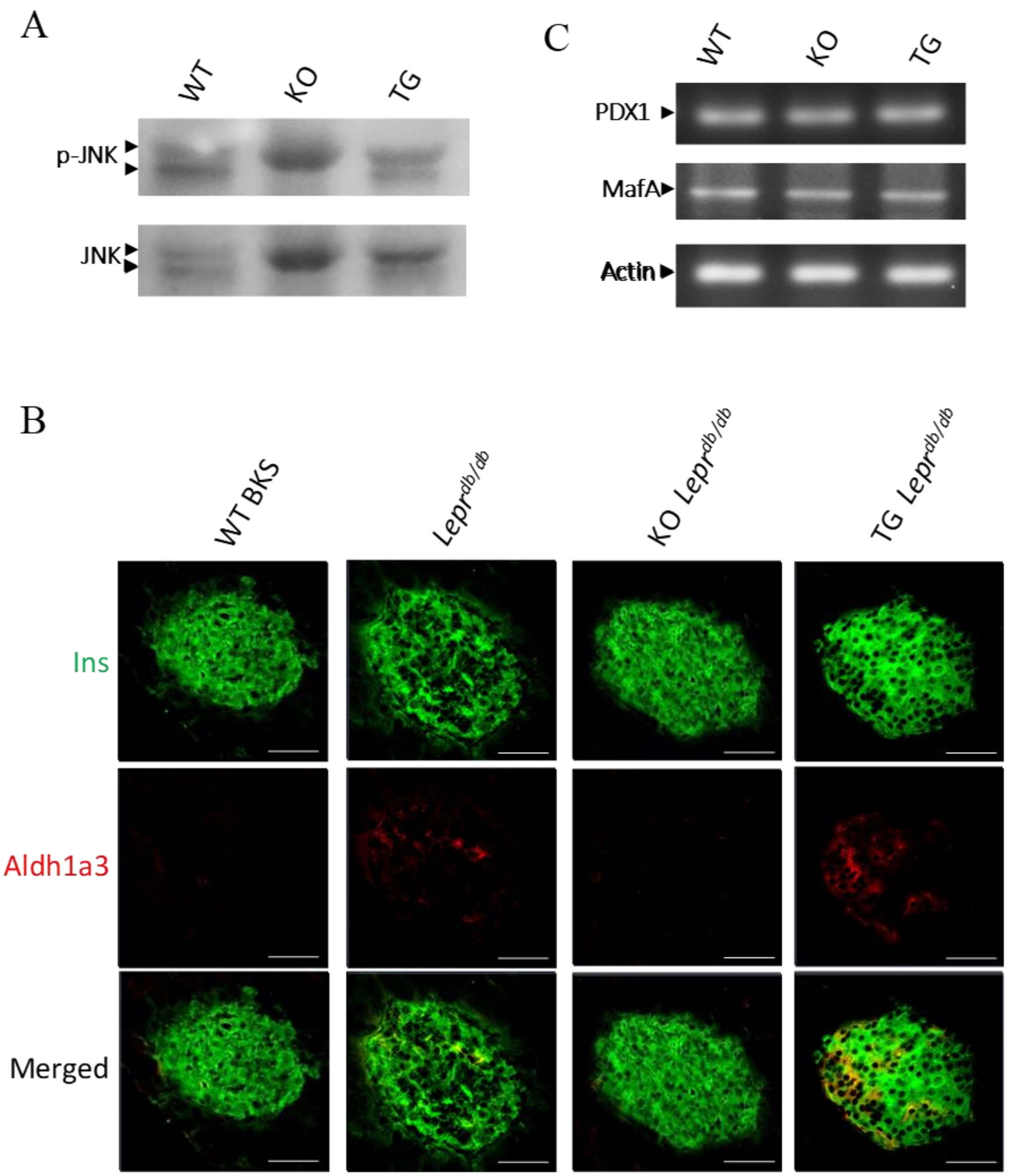

Appendix Figure S9. Effect of Pdia4 on the markers of cell death, dedifferentiation and differentiation in β cells. (A) Level of JNK and p-JNK in the islet cells of WT, *Pdia*$^{-/-}$ (KO) and Pdia4$^{tg/tg}$ (TG) BKS mice. The islet cells were harvested and lysed. Their total lysates were subjected to immunoblotting analysis using the indicated antibodies. (B) Confocal images of Aldh1a3, insulin (Ins) and both (Merged) of the islets were analyzed. The pancreatic sections of WT BKS, *Lepr*$^{db/db}$, *Pdia4*$^{-/-}$*Lepr*$^{db/db}$ (KO *Lepr*$^{db/db}$) and Pdia4$^{tg/tg}$*Lepr*$^{db/db}$ (TG *Lepr*$^{db/db}$) mice, aged 26-30 weeks, were stained with the indicated antibodies and the images were acquired using a confocal microscope. (C) Expression level of PDX1 and MafA in the islet cells of WT, *Pdia4*$^{-/-}$ (KO) and Pdia4$^{tg/tg}$ (TG) BKS mice. Total RNA of the WT, *Pdia4*$^{-/-}$ (KO) and Pdia4$^{tg/tg}$ TG) islets was isolated and converted into the first strand cDNA, followed by RT-PCR analysis.

Appendix Table S1. List of RNAi sequences

Gene	Sequence
GK (control)	CCGGGCTTGTGTTGACCAAAGAGAACTCGAGTTCTCTTTGGTCAACACAAGCTTTTTG
Pdia4	CCGGGCTTGTGTTGACCAAAGAGAACTCGAGTTCTCTTTGGTCAACACAAGCTTTTTG
p22^{phox}	CCGGCGTTTCACACAGTGGTATTTCCTCGAGGAAATACCACTGTGTGAAACGTTTTTG
Ndufs3	CCGGCTGACACCCATTGACTCTATACTCGAGTATAGAGTCAATGGGTGTCAGTTTTTG

Appendix Table S2. List of cDNA sequences

Gene	Sequence
Ndufs3	ATGGCGGCGGCTGCAGCCAGGGTCTGGTGTCGTGGGCTCTTGGGGGCCGCTTCCGTAG<br>GCAGGGGGGCTGGGCGACCCTCCGTGCTGTGGCAGCACGTAAGAAGGGAGAGCGCGGC<br>GGCTGACAAGCGCCCCACTGTCAGACCCCGGAGTGATGTGACCCACAAGCAGCTCTCA<br>GCATTTGGAGAGTATGTGGCTGAAATCTTACCCAAGTATGTCCAACAAGTTCAGGTGT<br>CCTGCCTTGATGAGTTAGAAATCTGTATCCATCCCGATGGAGTCATCCCAACGCTGAC<br>TTTTCTCAGGGATCACACCAATGCACAATTCAAATCCTTGGCTGACTTGACGGCAGTG<br>GATGTCCCAACTCGGCAGAACCGTTTTGAGATTGTCTACAACCTGCTGTCTCTGCGGT<br>TCAACTCTAGGATTCGTGTGAAGACCTATGCAGATGAGCTGACACCCATTGACTCTAT<br>AGTGTCTGTGCACATCGCGGCCAATTGGTATGAGAGGGAGGTCTGGGACATGTTTGGA<br>GTTTTCTTTTTTAACCACCCTGATTTAAGAAGGATCCTGACAGATTATGGCTTCGAGG<br>GACATCCTTTCCGGAAAGACTTTCCCCTCACTGGCTATGTTGAGCTTCGTTACGACGA<br>TGAGGTAAAGCGGGTAGTGGCTGAACCAGTGGAGCTGGCACAAGAATTCCGCAAGTTT<br>GACCTGAACAGCCCCTGGGAGGCTTTCCCTGCCTATCGCCAGCCTCCTGAGAGTCTCA<br>AGCTCGAAGCTGGAGACAAGAAGCCTGAAACCAAGTAA
tNdufs3	ATGGCGGCGGCTGCAGCCAGGGTCTGGTGTCGTGGGCTCTTGGGGGCCGCTTCCGTAG<br>GCAGGGGGGCTGGGCGACCCTCCGTGCTGTGGCAGCACGTAAGAAGGGAGAGCGCGGC<br>GGCTGACAAGCGCCCCACTGTCAGACCCCGGAGTGATGTGACCCACAAGCAGCTCTCA<br>GCATTTGGAGAGTATGTGGCTGAAATCTTACCCAAGTATGTCCAACAAGTTCAGGTGT<br>CCTGCCTTGATGAGTTAGAAATCTGTATCCATCCCGATGGAGTCATCCCAACGCTGAC<br>TTTTCTCAGGGATCACACCAATGCACAATTCAAATCCTTGGCTGACTTGACGGCAGTG<br>GATGTCCCAACTCGGCAGAACCGTTTTGAGATTGTCTACAACCTGCTG
p22^{phox}	ATGGGGCAGATCGAGTGGGCCATGTGGGCCAACGAACAGGCGCTGGCGTCTGGCCTGA<br>TTCTCATCACTGGGGGCATCGTGGCTACTGCTGGACGTTTCACACAGTGGTATTTCGG<br>CGCCTACTCTATCGCTGCAGGTGTGCTCATCTGTCTGCTGGAGTATCCCCGGGGAAAG<br>AGGAAAAAGGGGTCCACCATGGAGCGATGTGGACAGAAGTACCTGACCTCTGTGGTGA<br>AGCTTTTCGGGCCCCTCACCAGGAATTACTACGTCCGGGCTGCCCTCCACTTCCTGTT<br>GTCGGTGCCTGCAGGCTTCCTCCTGGCCACCATCCTGGGGACCGTCTGCTTGGCCATT<br>GCCAGTGTGATCTATCTGCTGGCAGCCATCCGAGGTGAGCAGTGGACTCCCATTGAGC<br>CTAAACCCAAGGAGCGGCCACAGGTTGGAGGCACCATCAAGCAACCACCTACCAACCC<br>CCCACCCCGGCCACCCGCAGAGGTCCGAAAGAAGCCGAGTGAGGGTGAAGAGGAGGCA<br>GCCTCAGCTGGAGGACCCCAGGTTAACCCAATGCCAGTGACAGATGAGGTCGTGTGA
tp22^{phox}	ATGGGGCAGATCGAGTGGGCCATGTGGGCCAACGAACAGGCGCTGGCGTCTGGCCTGA<br>TTCTCATCACTGGGGGCATCGTGGCTACTGCTGGACGTTTCACACAGTGGTATTTCGG<br>CGCCTACTCTATCGCTGCAGGTGTGCTCATCTGTCTGCTGGAGTATCCCCGGGGAAAG<br>AGGAAAAAGGGGTCCACCATGGAGCGATGTGGACAGAAGTACCTGACCTCTGTGGTGA<br>AGCTTTTCGGGCCCCTCACCAGGAATTACTACGTCCGGGCTGCC<br>CTCCACTTCCTGTTGTCGGTG
Pdia4	ATGAGGCCCCGGAAAGCCTTCCTGCTCCTGCTGCTCTTGGGGCTGGTGCAGCTGCTGG<br>CCGTGGCGGGTGCCGAGGGCCCGGACGAGGATTCTTCTAACAGAGAAAATGCCATTGA<br>GGATGAAGAGGAGGAGGAGGAGGAAGATGATGATGAGGAAGAAGACGACTTGGAAGTT<br>AAGGAAGAAAATGGAGTCTTGGTCCTAAATGATGCAAACTTTGATAATTTTGTGGCTG<br>ACAAAGACACAGTGCTGCTGGAGTTTTATGCTCCATGGTGTGGACATTGCAAGCAGTT<br>TGCTCCGGAATATGAAAAAATTGCCAACATATTAAAGGATAAAGATCCTCCCATTCCT<br>GTTGCCAAGATCGATGCAACCTCAGCGTCTGTGCTGGCCAGCAGGTTTGATGTGAGTG<br>GCTACCCCACCATCAAGATCCTTAAGAAGGGGCAGGCTGTAGACTACGAGGGCTCCAG<br>AACCCAGGAAGAAATTGTTGCCAAGGTCAGAGAAGTCTCCCAGCCCGACTGGACGCCT<br>CCACCAGAAGTCACGCTTGTGTTGACCAAAGAGAACTTTGATGAAGTTGTGAATGATG

	CAGATATCATTCTGGTGGAGTTTTATGCCCCATGGTGTGGACACTGCAAGAAACTTGC<br>CCCCGAGTATGAGAAGGCCGCCAAGGAGCTCAGCAAGCGTTCTCCTCCAATTCCCCTG<br>GCAAAGGTCGACGCCACCGCAGAAACAGACCTGGCCAAGAGGTTTGATGTCTCTGGCT<br>ATCCCACCCTGAAAATTTTCCGCAAAGGAAGGCCTTATGACTACAACGGCCCACGAGA<br>AAAATATGGAATCGTTGATTACATGATCGAGCAGTCCGGGCCTCCCTCCAAGGAGATT<br>CTGACCCTGAAGCAGGTCCAGGAGTTCCTGAAGGATGGAGACGATGTCATCATCATCG<br>GGGTCTTTAAGGGGGAGAGTGACCCAGCCTACCAGCAATACCAGGATGCCGCTAACAA<br>CCTGAGAGAAGATTACAAATTTCACCACACTTTCAGCACAGAAATAGCAAAGTTCTTG<br>AAAGTCTCCCAGGGGCAGTTGGTTGTAATGCAGCCTGAGAAATTCCAGTCCAAGTATG<br>AGCCCCGGAGCCACATGATGGACGTCCAGCAGGGCTCCACCCAGGACTCGGCCATCAA<br>GGACTTCGTGCTGAAGTACGCCCTGCCCCTGGTTGGCCACCGCAAGGTGTCAAACGAT<br>GCTAAGCGCTACACCAGGCGCCCCCTGGTGGTCGTCTACTACAGTGTGGACTTCAGCT<br>TTGATTACAGAGCTGCAACTCAGTTTTGGCGGAGCAAAGTCCTAGAGGTGGCCAAGGA<br>CTTCCCTGAGTACACCTTTGCCATTGCGGACGAAGAGGACTATGCTGGGGAGGTGAAG<br>GACCTGGGGCTCAGCGAGAGTGGGGAGGATGTCAATGCCGCCATCCTGGACGAGAGTG<br>GGAAGAAGTTCGCCATGGAGCCAGAGGAGTTTGACTCTGACACCCTCCGCGAGTTTGT<br>CACTGCTTTCAAAAAAGGAAAACTGAAGCCAGTCATCAAATCCCAGCCAGTGCCCAAG<br>AACAACAAGGGACCCGTCAAGGTCGTGGTGGGAAAGACCTTTGACTCCATTGTGATGG<br>ACCCCAAGAAGGACGTCCTCATCGAGTTCTACGCGCCATGGTGCGGGCACTGCAAGCA<br>GCTAGAGCCCGTGTACAACAGCCTGGCCAAGAAGTACAAGGGCCAAAAGGGCCTGGTC<br>ATCGCCAAGATGGACGCCACTGCCAACGACGTCCCCAGCGACCGCTATAAGGTGGAGG<br>GCTTCCCCACCATCTACTTCGCCCCCAGTGGGGACAAAAAGAACCCAGTTAAATTTGA<br>GGGTGGAGACAGAGATCTGGAGCATTTGAGCAAGTTTATAGAAGAACATGCCACAAAA<br>CTGAGCAGGACCAAGGAAGAGCTTTGA

Appendix Table S3. Antibodies used in this study

Antibody	Company	Catalog No.	Application
Pdia4	Enzo	ADI-SPS-720-D	IB (1:2000), IHC, (1:1000), IP (5 μg)
$p22^{phox}$	Santa Cruz	sc-20781	IB (1:1000)
Ndufs3	Proteintech	15066-1-AP	IB (1:1000)
Myc	Thermo Fisher	MA1-980	IB (1 μg/ml), IP (5 μg)
Flag	OriGene	TA50011-100	IB (1 μg/ml), IP (5 μg)
Actin	Santa Cruz	sc-376421	IB (1:5000)
PARP	Cell Signaling	9542	IB (1:1000)
Na/K ATPase	Thermo Fisher	ST0533	IB (1:1000)
Grp94	Thermo Fisher	MA3-016	IB (1:1000)
Insulin	Abcam	ab63820	IHC (1:500)
Glucagon	Boster	M00678-1	IHC (1:500)
Shdb	Abcam	ab175225	IB (1:1000)
Uqcrfs1	Proteintech	18443-1-AP	IB (1:1000)
Cox5b	Proteintech	11418-2-AP	IB (1:1000)
Nox2	Proteintech	19013-1-AP	IB (1:1000)
$p47^{phox}$	LSBio	LS-B2365	IB (1:1000)
$p67^{phox}$	Proteintech	15551-1-AP	IB (1:1000)
Rac1	Proteintech	24072-1-AP	IB (1:1000)
Pdia1	Proteintech	11245-1-AP	IB (1:1000)
Pdia3	Proteintech	15967-1-AP	IB (1:1000)
Pdia6	Proteintech	18233-1-AP	IB (1:1000)

Gst	Santa Cruz	sc-138	IB (1 μg/ml)
His	Invitrogen	MA1-21315	IB (1 μg/ml)

Appendix Table S4. PCR primers used in this study

Gene	Primer pair
Pdia1	5′-GAGTTTTGCCACCGCTTCTTAG-3′ , 5′-AAAGTTCGCCCCAACCAGTAC-3′
Pdia2	5′- GCTTCACTGACCAGCCACAACT -3′, 5′-TCCAGGCCAGTCTCCTTGTCTA-3′
Pdia3	5′-TGGCCACTGTAAGAATCTGGAA-3′, 5′-TGGAGAAGGCACATCATTGG-3′
Pdia4	5′-AAGGTGGTGGTGGGAAAG-3′, 5′-GATGTCGTTGGCAGTAGC-3′
Pdia5	5′-TGCCTGCGCTCAGAACAAA-3′, 5′-GCACGCTTGTCTGCTGTTCTT-3′
Pdia6	5′-TGGATGCCACCATGAATCAG-3′, 5′-TCGTCCGACCACCATCATAGTC-3′
Pdia13	5′-AAGCTATTAAAGGGAGACTTGGCATAT-3′,5′-CGAACATTTGTTGACTTGGAAGAG-3′
Pdia15	5′-CAGGCTTGTCAGATGTCACCAT-3′, 5′-CCGTTGTGTTCTCCCACTTTT-3′
Pdia16	5′-GCTCCTCATCACTTCTTCAG-3′ , 5′-CCATCAGCGGCAAGCCACT-3′
Pdia19	5′-GGAACCATGGCTTGTTGACTTC-3′ , 5′-GCAGTGTTGATGCTTTCCGTAA-3′
L13	5′-AGA TAC CAC ACC AAG GTC CG-3′, 5′-GGA GCA GAA GGC TTC CTG-3′
