# Supplementary figures and images for: Pdia4 regulates β‐cell pathogenesis in diabetes: molecular mechanism and targeted therapy
Source: EMBO Mol Med. 2021 Sep 20;13(10):e11668. doi: 10.15252/emmm.201911668 (PMC8495450; doi:10.15252/emmm.201911668)

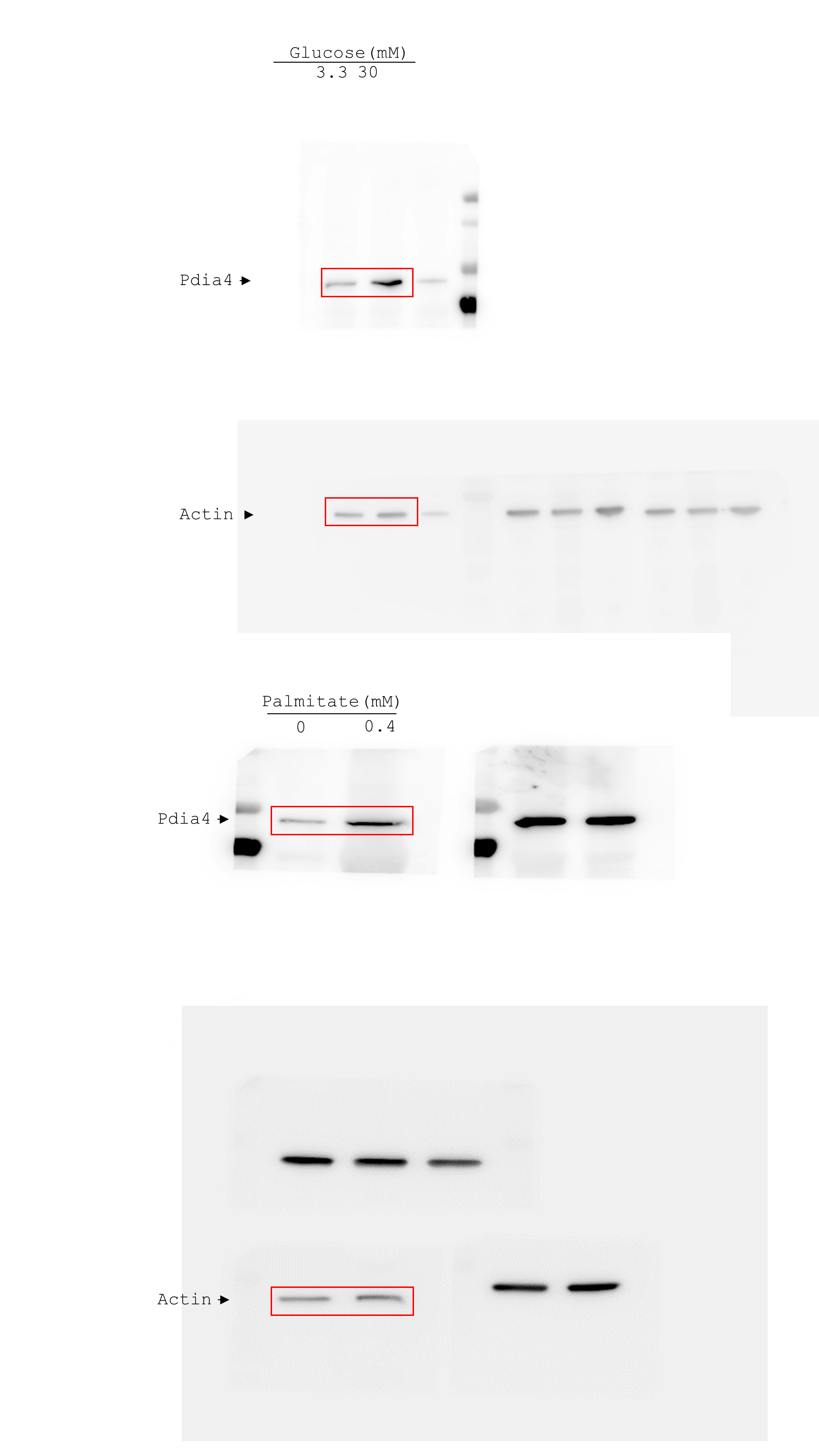

Supplement: Supplementary file 2 — Source Data for Figure 1 [file EMMM-13-e11668-s006.zip › Source_data_Fig._1D.tiff]

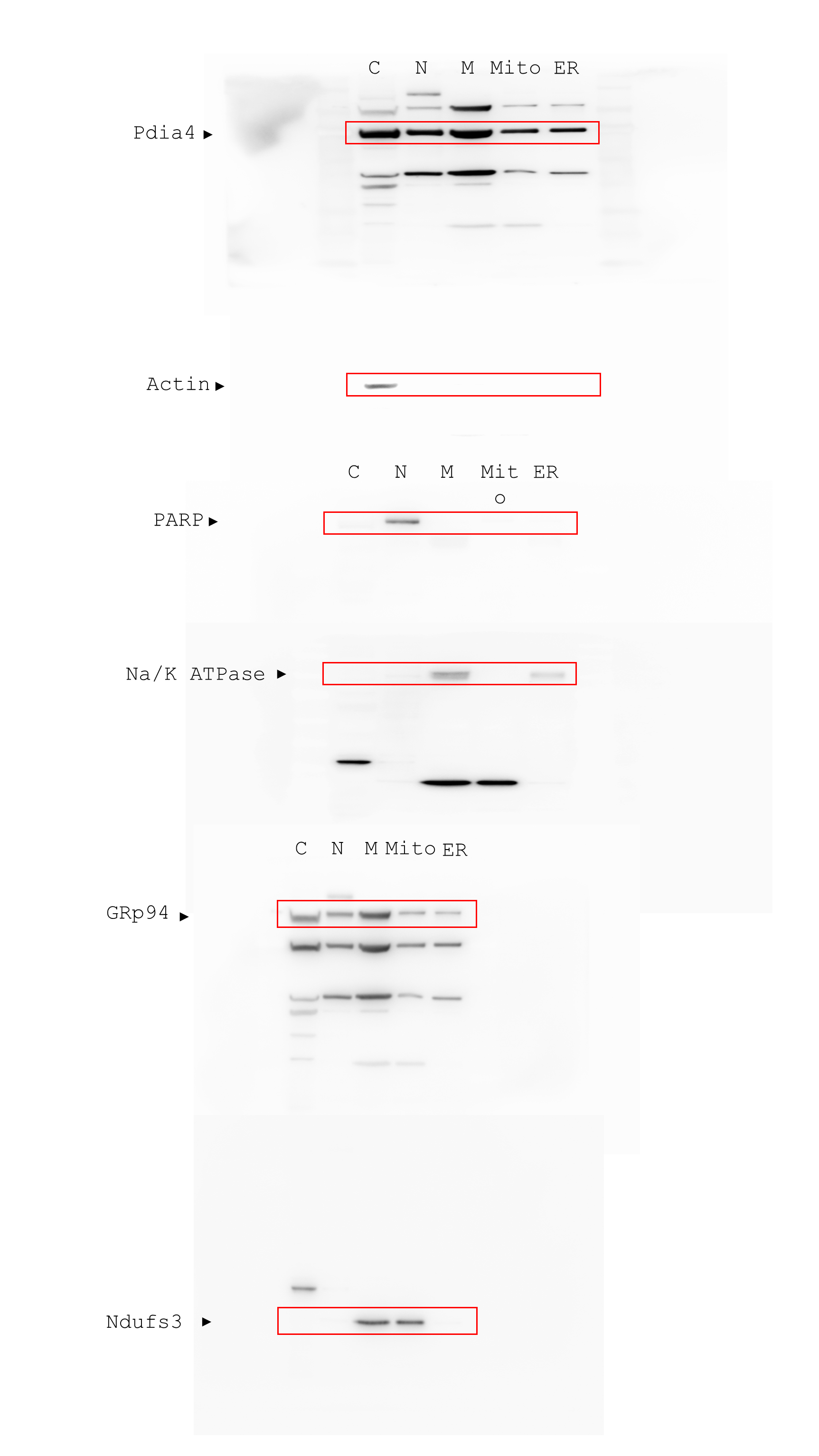

Supplement: Supplementary file 2 — Source Data for Figure 1 [file EMMM-13-e11668-s006.zip › Source_data_Fig._1H.tiff]

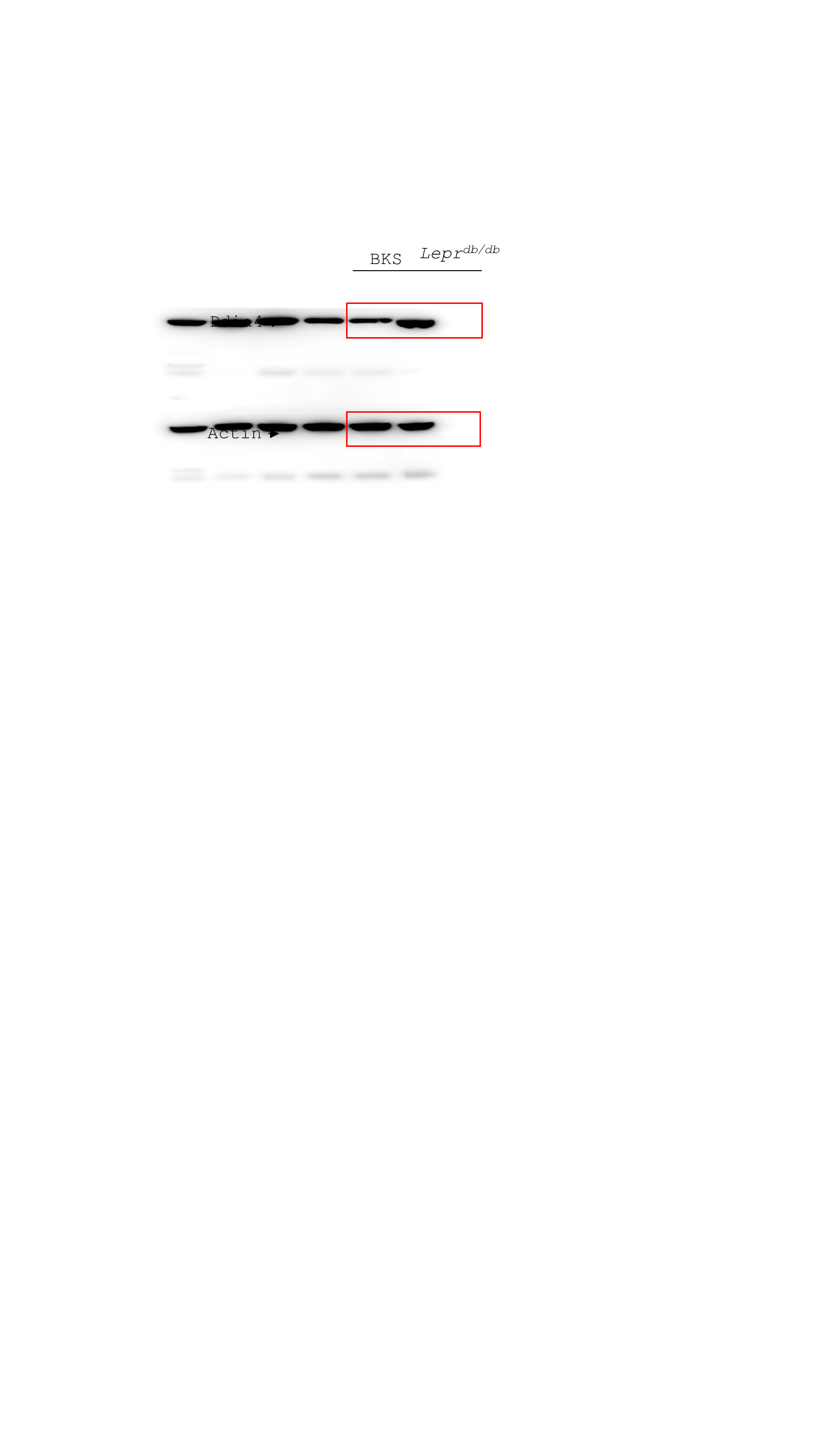

Supplement: Supplementary file 2 — Source Data for Figure 1 [file EMMM-13-e11668-s006.zip › Source_data_Fig._1E.tiff]

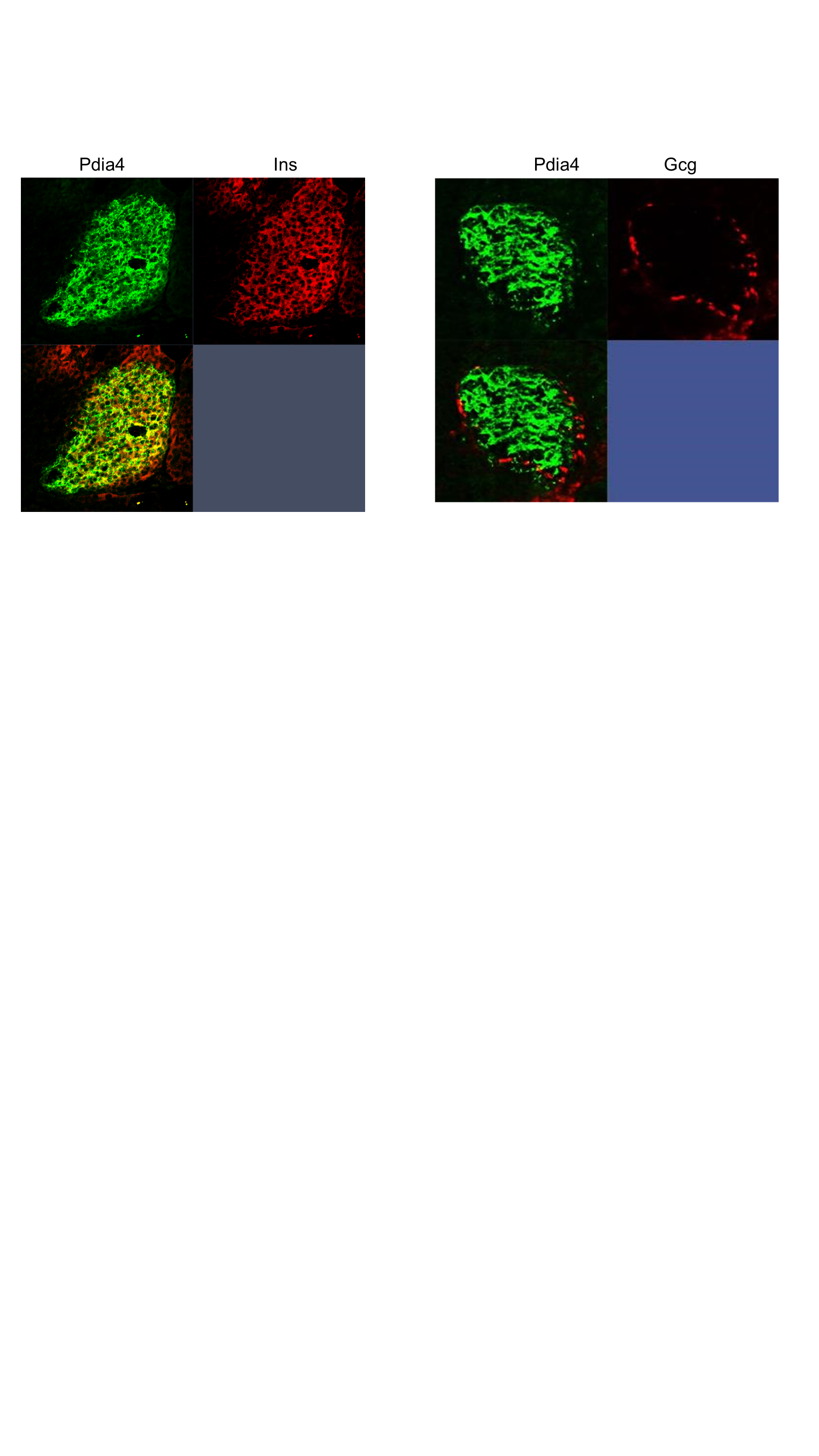

Supplement: Supplementary file 2 — Source Data for Figure 1 [file EMMM-13-e11668-s006.zip › Source_data_Fig._1B.tiff]

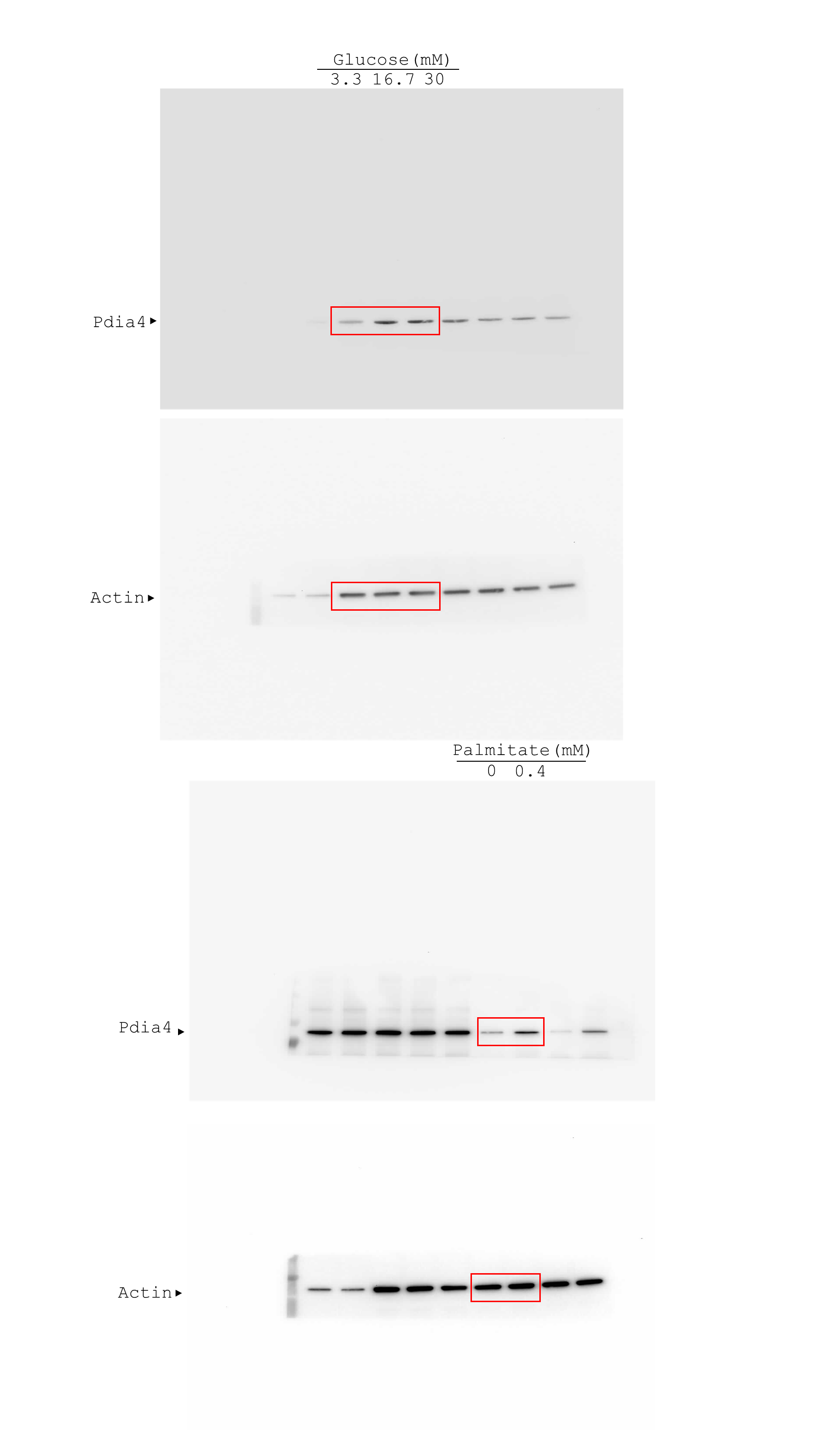

Supplement: Supplementary file 2 — Source Data for Figure 1 [file EMMM-13-e11668-s006.zip › Source_data_Fig._1C.tiff]

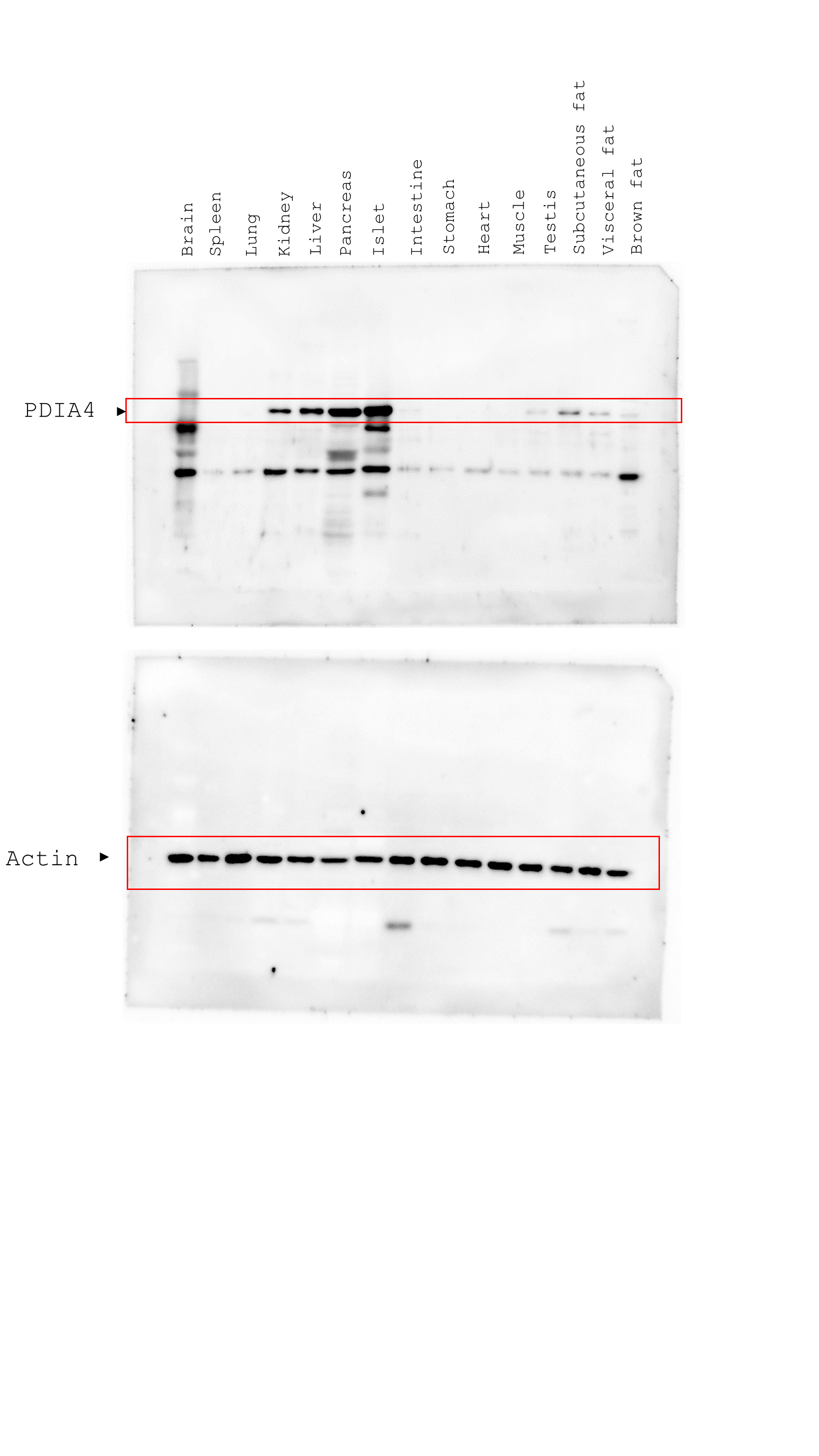

Supplement: Supplementary file 2 — Source Data for Figure 1 [file EMMM-13-e11668-s006.zip › Source_data_Fig._1A.tiff]

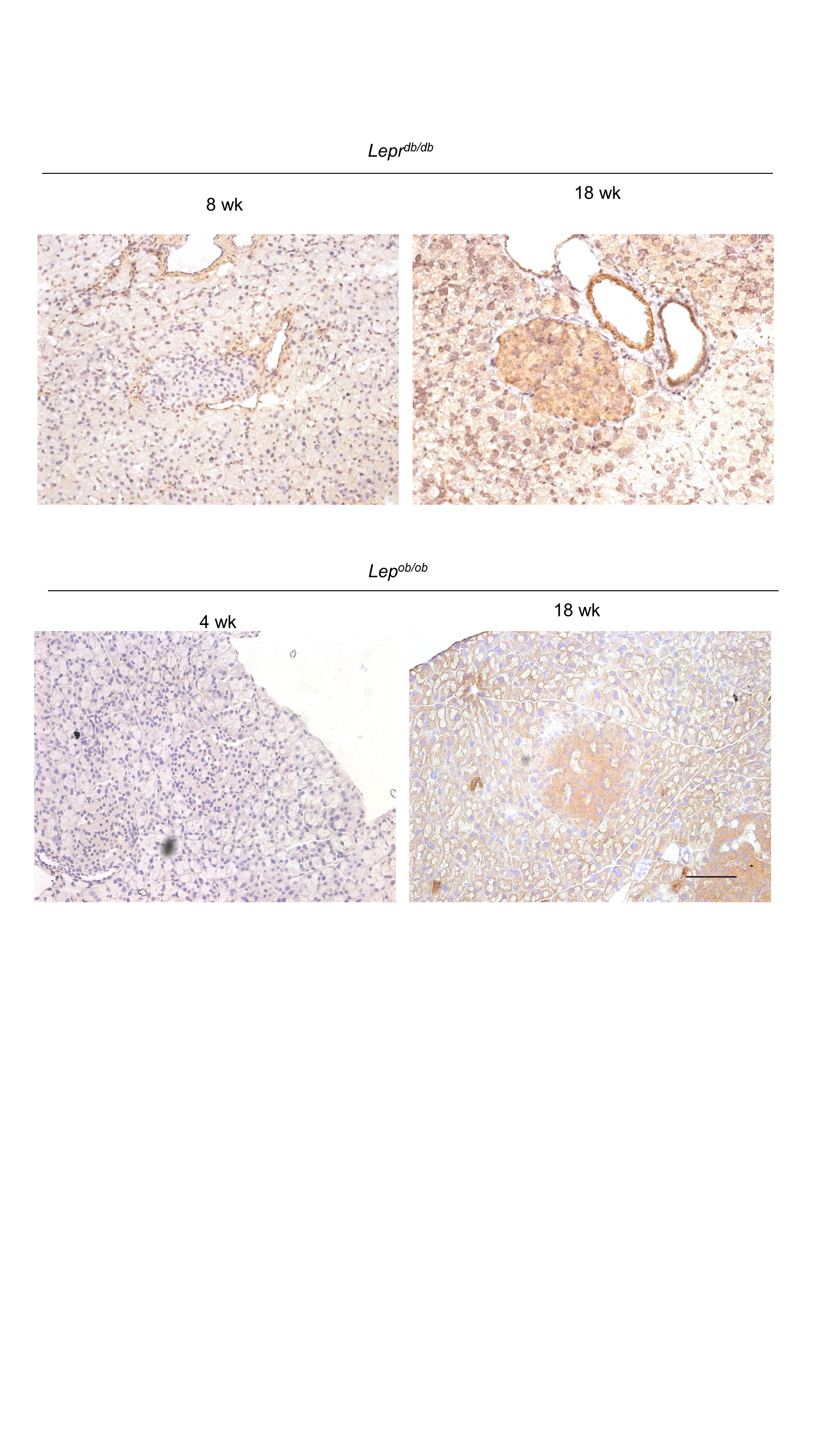

Supplement: Supplementary file 2 — Source Data for Figure 1 [file EMMM-13-e11668-s006.zip › Source_data_Fig._1F.tiff]

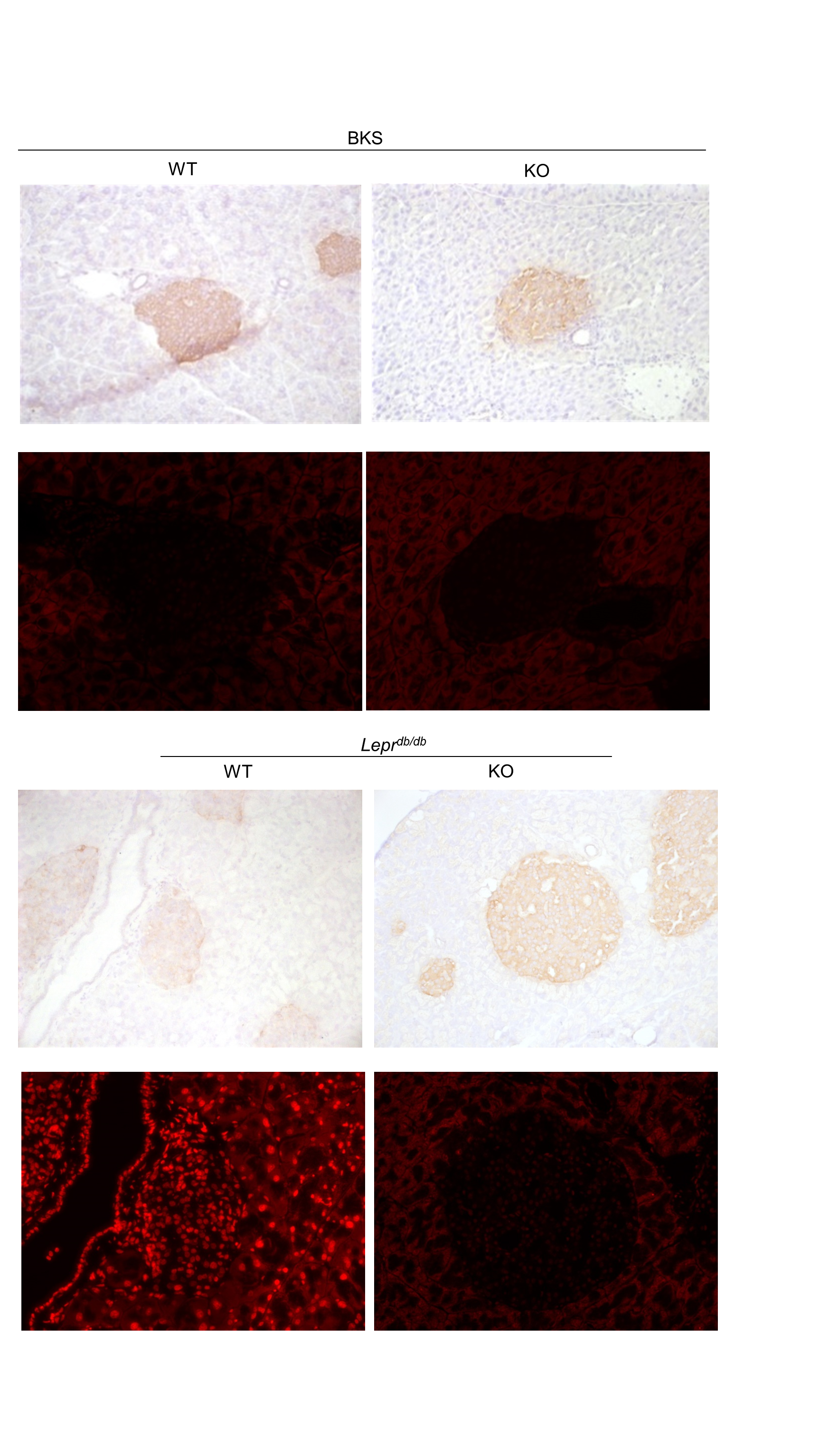

Supplement: Supplementary file 3 — Source Data for Figure 2 [file EMMM-13-e11668-s003.zip › Source_data_Fig._2B.tiff]

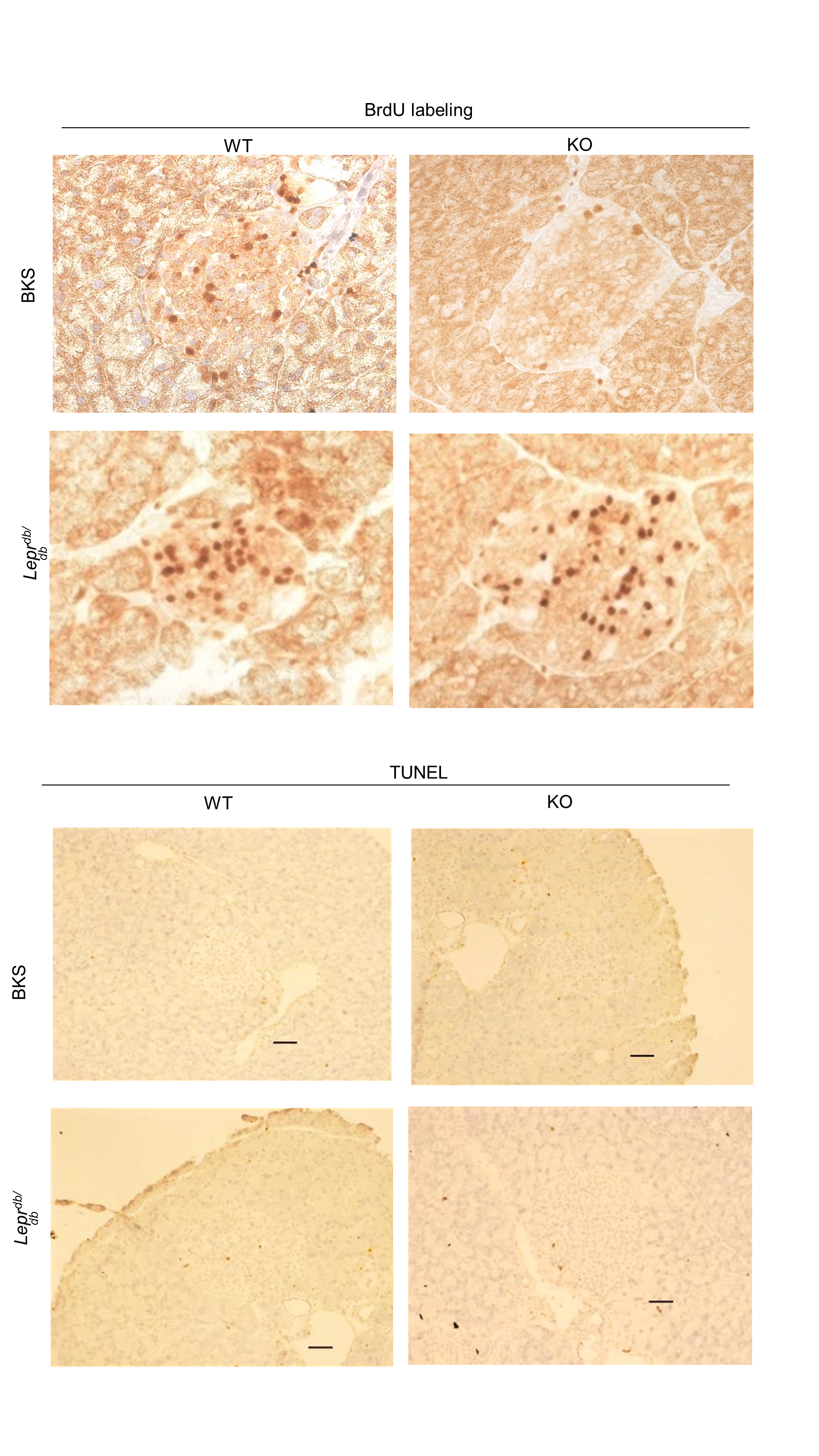

Supplement: Supplementary file 3 — Source Data for Figure 2 [file EMMM-13-e11668-s003.zip › Source_data_Fig._2D.tiff]

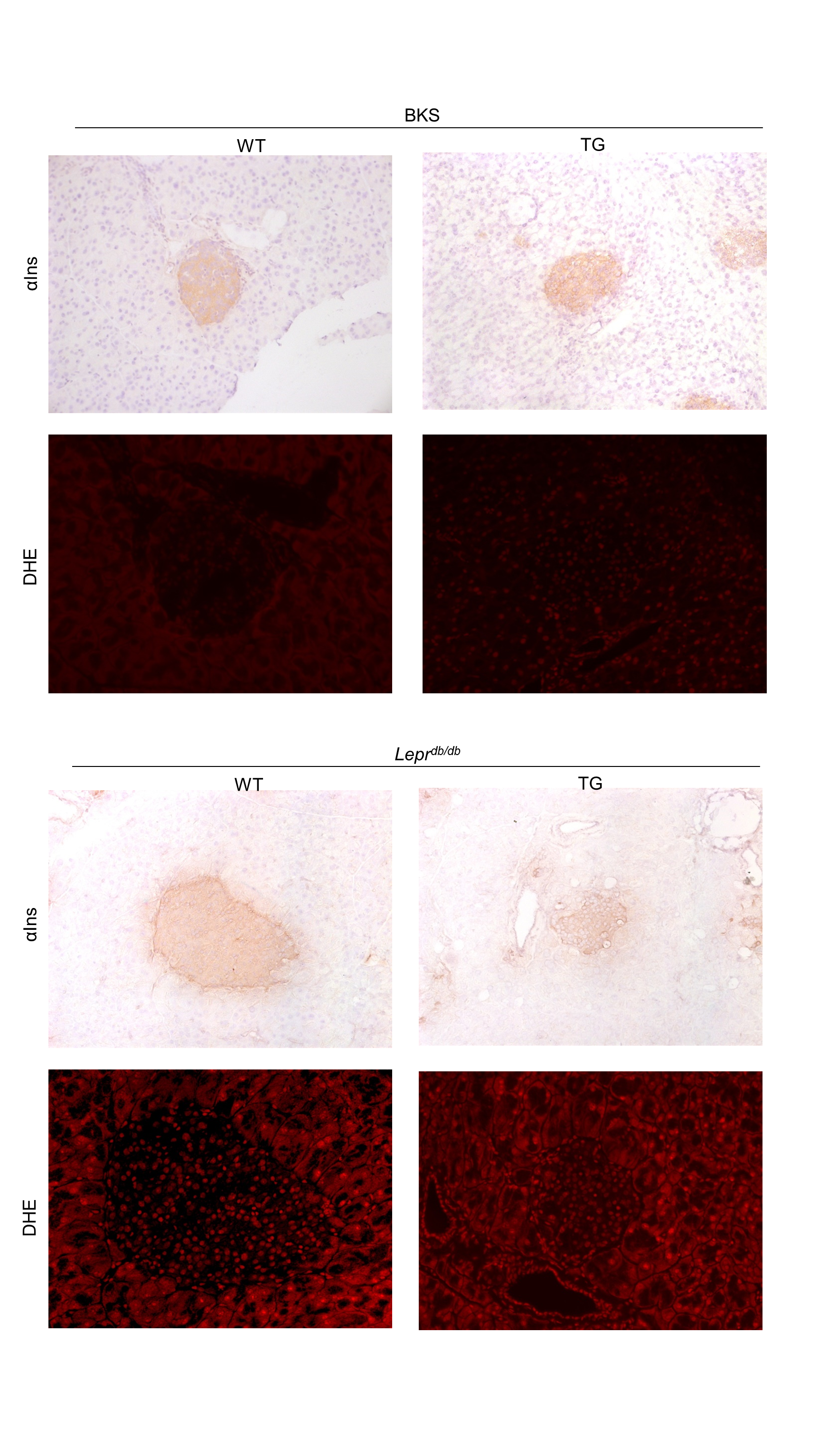

Supplement: Supplementary file 4 — Source Data for Figure 3 [file EMMM-13-e11668-s005.TIF]

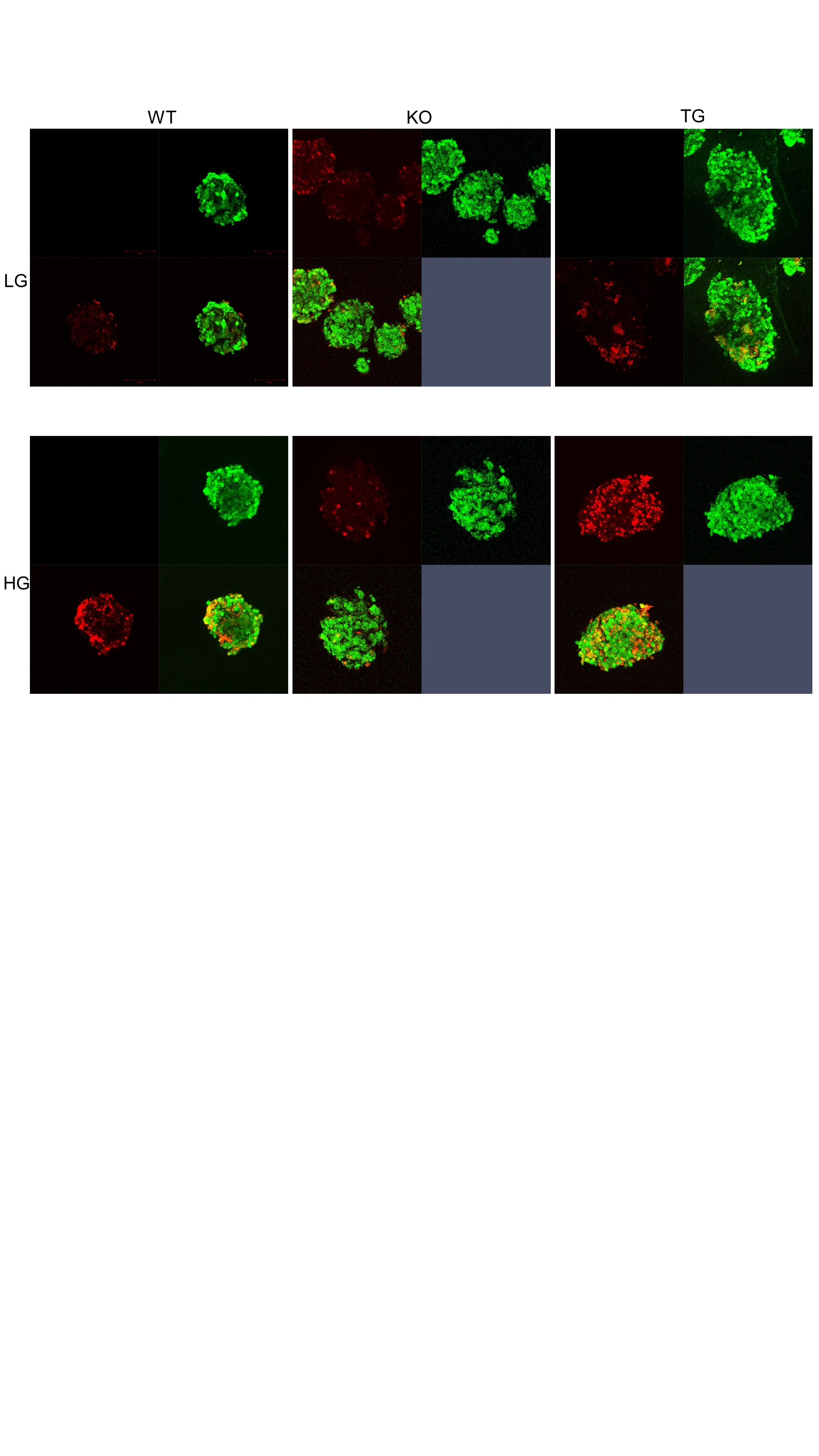

Supplement: Supplementary file 5 — Source Data for Figure 4 [file EMMM-13-e11668-s001.zip › Source_data_Fig._4C.tiff]

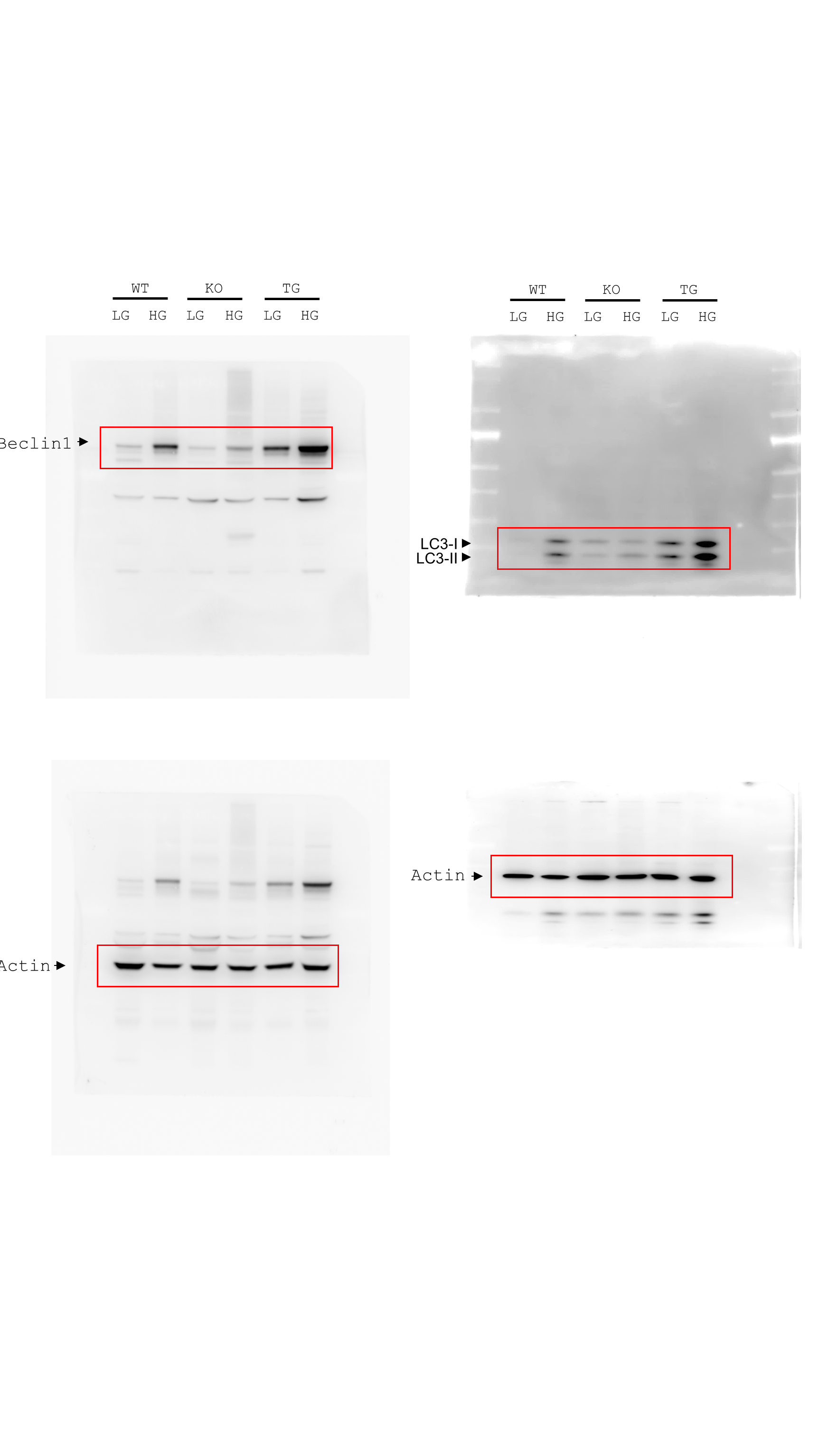

Supplement: Supplementary file 5 — Source Data for Figure 4 [file EMMM-13-e11668-s001.zip › Source_data_Fig._4B.tiff]

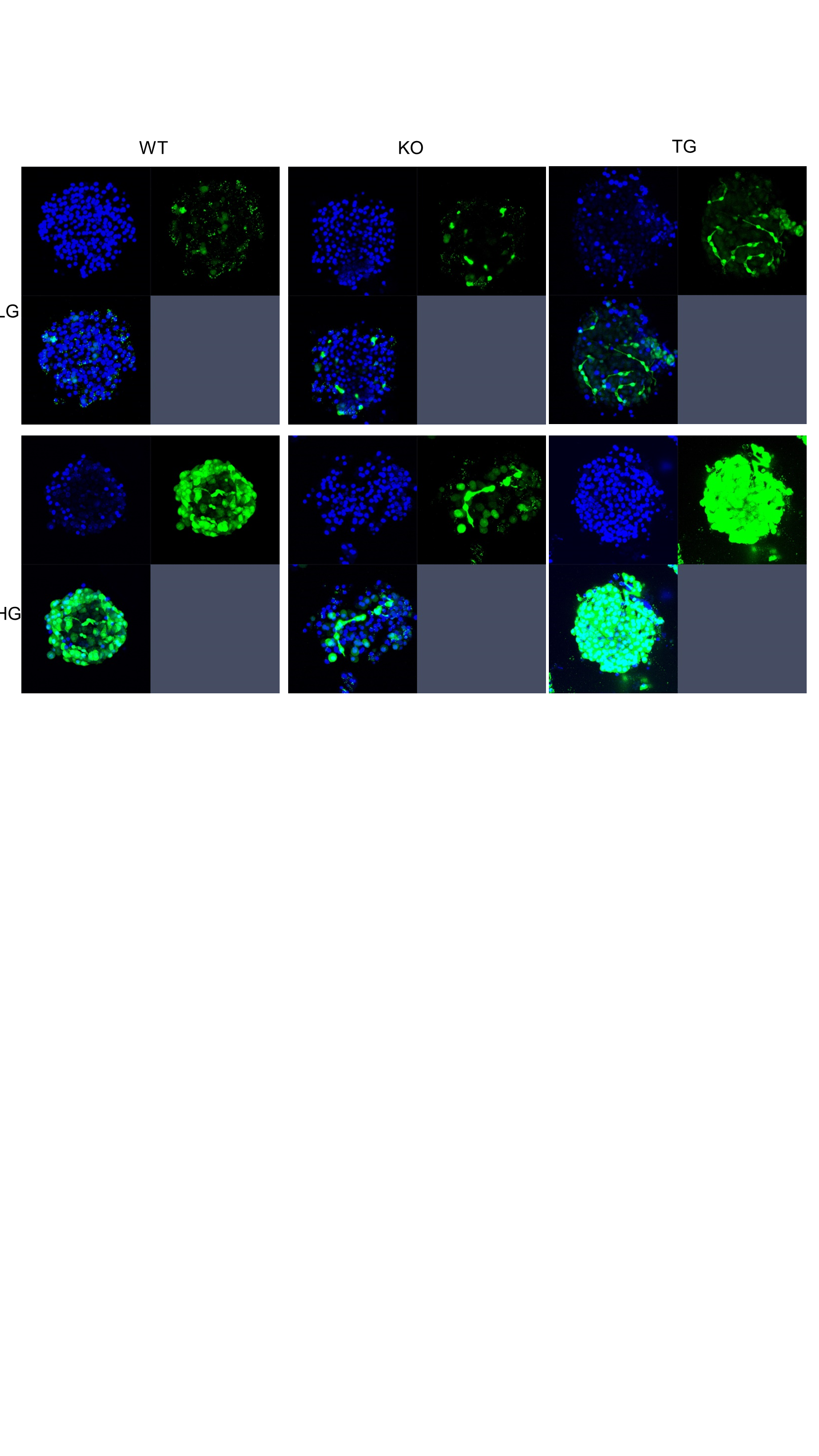

Supplement: Supplementary file 5 — Source Data for Figure 4 [file EMMM-13-e11668-s001.zip › Source_data_Fig._4D.tiff]

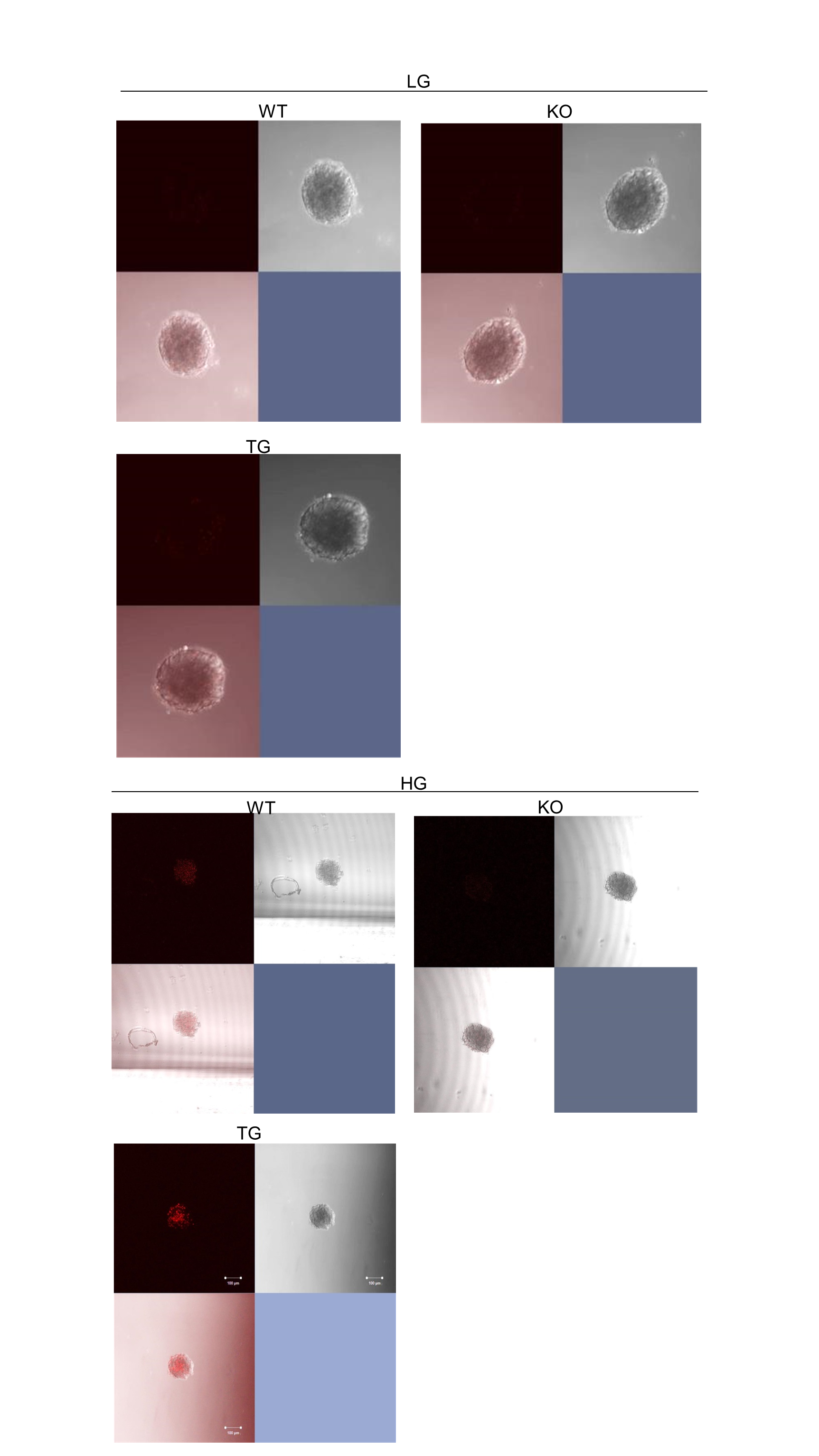

Supplement: Supplementary file 5 — Source Data for Figure 4 [file EMMM-13-e11668-s001.zip › Source_data_Fig._4A.tiff]

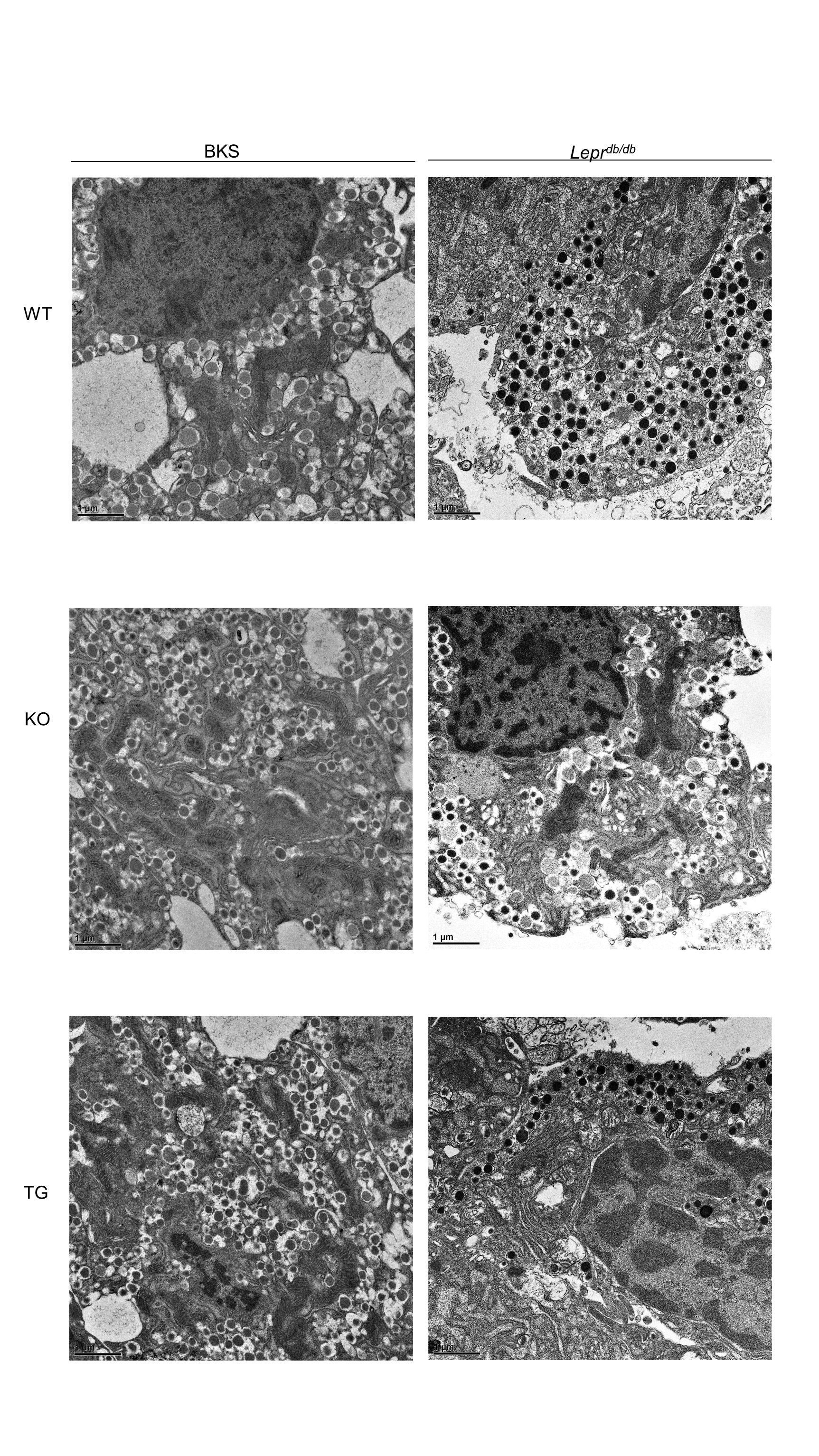

Supplement: Supplementary file 6 — Source Data for Figure 5 [file EMMM-13-e11668-s008.TIF]

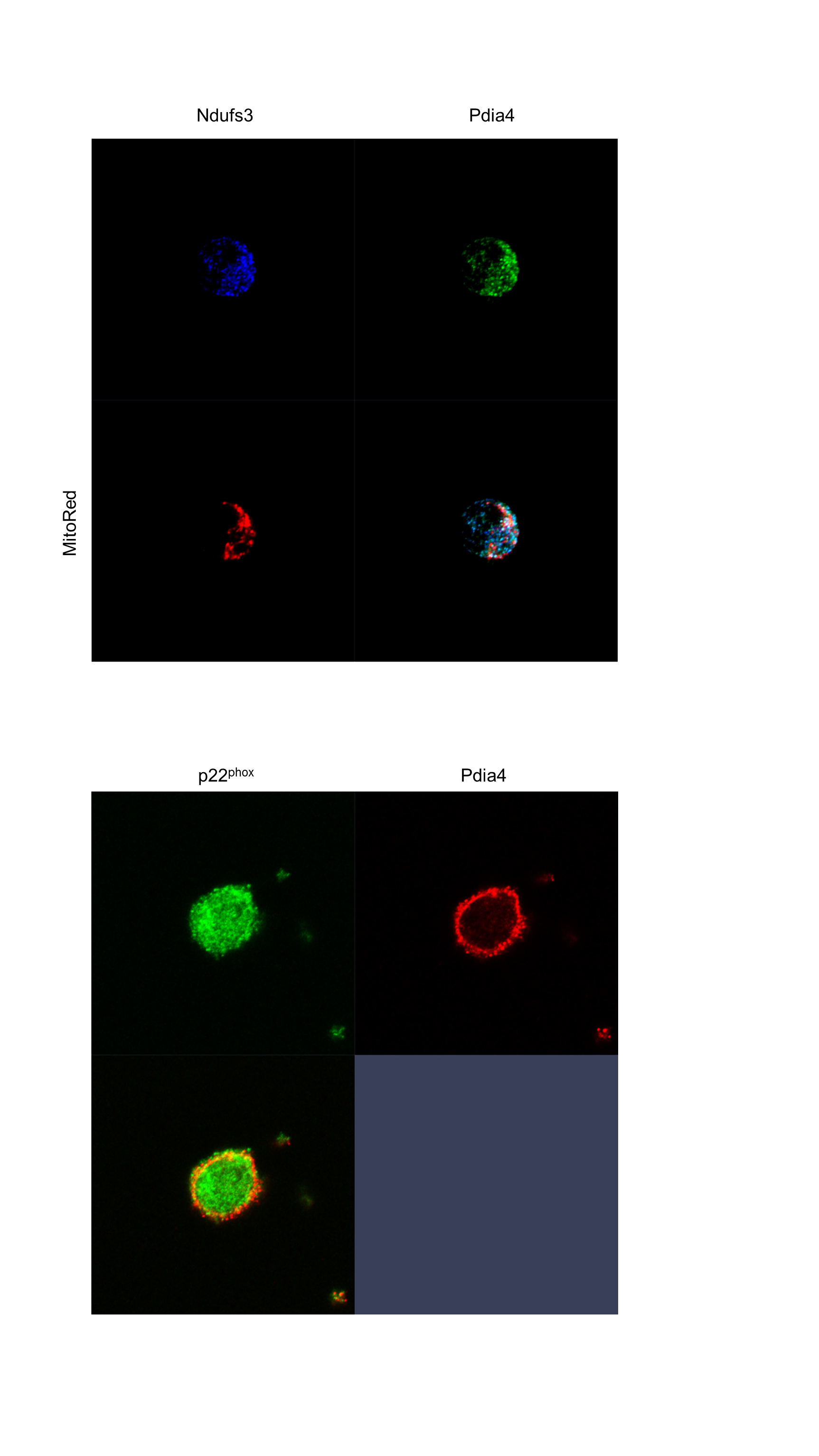

Supplement: Supplementary file 7 — Source Data for Figure 6 [file EMMM-13-e11668-s002.TIF]

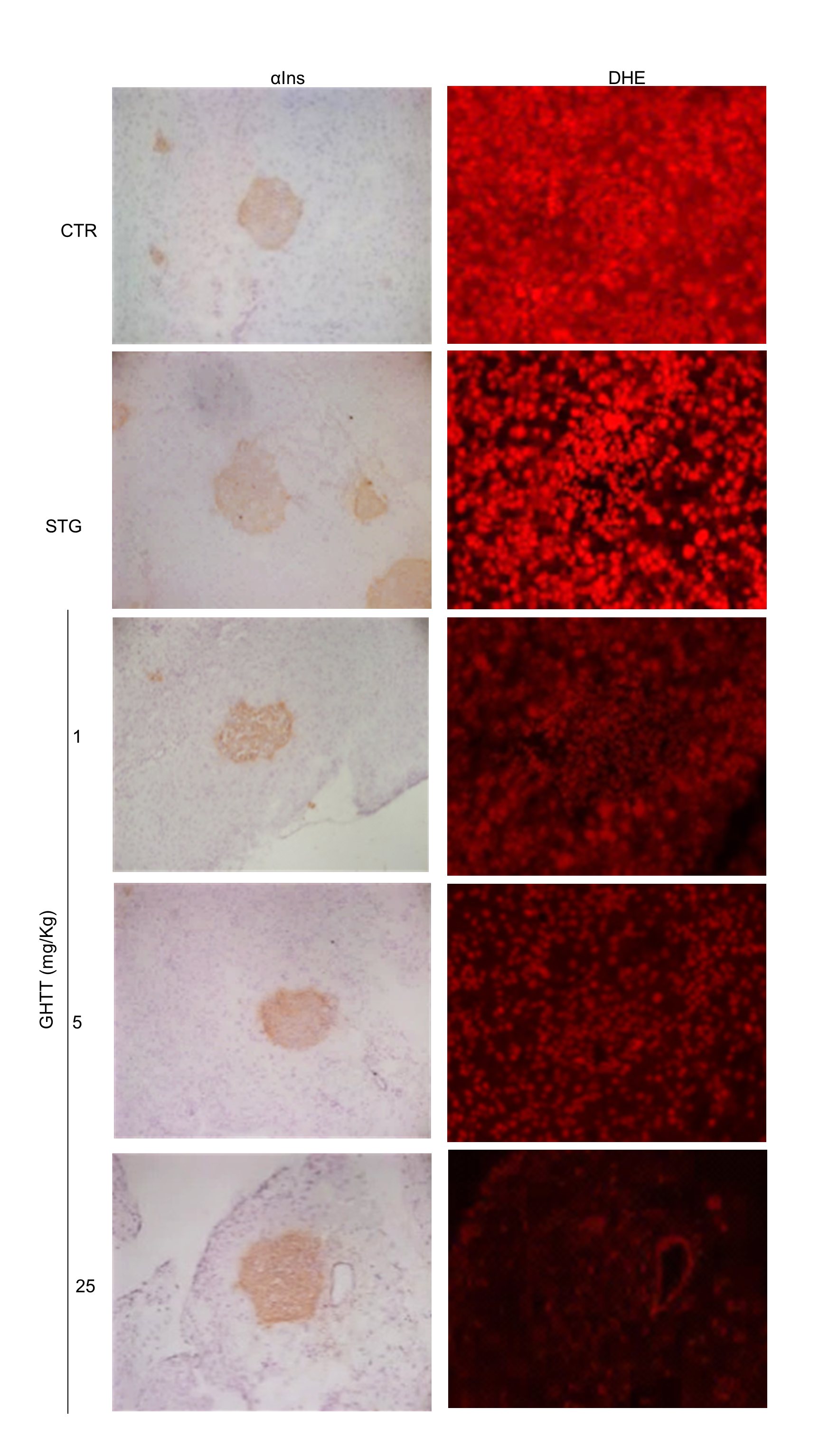

Supplement: Supplementary file 8 — Source Data for Figure 8 [file EMMM-13-e11668-s007.TIF]
