# Supplementary material for: Pdia4 regulates β‐cell pathogenesis in diabetes: molecular mechanism and targeted therapy
Source: EMBO Mol Med. 2021 Sep 20;13(10):e11668. doi: 10.15252/emmm.201911668 (PMC8495450; doi:10.15252/emmm.201911668)
Supplement: Supplementary file 9 [file EMMM-13-e11668-s004.pdf]

EMBO Molecular Medicine

Pdia4 regulates β-cell pathogenesis in diabetes: molecular mechanism and targeted therapy

Tien-Fen Kuo, Shuo-Wen Hsu, Shou-Hsien Huang, Cicero Lee-Tian Chang, Ching-Shan Feng, Ming-Guang Huang, Tzung-Yan Chen, Meng-Ting Yang, Si-Tse Jiang, Tuan-Nan Wen, Chun-Yen Yang, Chung-Yu Huang, Shu-Huei Kao, Keng-Chang Tsai, Greta Yang and Wen-Chin Yang
DOI: 10.15252/emmm.202013859

Corresponding author: Wen-Chin Yang (wcyang@gate.sinica.edu.tw)

Review Timeline:

Submission Date:	27th Oct 19
Editorial Decision:	26th Nov 19
Revision Received:	20th Dec 20
Editorial Decision:	8th Mar 21
Revision Received:	4th May 21
Editorial Decision:	19th May 21
Revision Received:	17th Aug 21
Accepted:	18th Aug 21

Editor: Jingyi Hou

Transaction Report:

(Note: With the exception of the correction of typographical or spelling errors that could be a source of ambiguity, letters and reports are not edited. Depending on transfer agreements, referee reports obtained elsewhere may or may not be included in this compilation. Referee reports are anonymous unless the Referee chooses to sign their reports.)

26th Nov 2019

Decision on your manuscript EMM-2019-11668

Dear Dr. Yang,

Thank you for submitting your work to EMBO Molecular Medicine. We have now received feedback from the three reviewers whom we asked to evaluate your manuscript. As you will see below, the reviewers raise substantial concerns about your work, which unfortunately preclude its publication in EMBO Molecular Medicine.

Overall, the reviewers acknowledge the potential interest of the study. However, they raise serious concerns with regard to the adequacy of the mouse models, the lack of in-depth mechanistic insights, unclear relevance to human diabetes, as well as the preliminary nature of the GHTT inhibitor studies. I am therefore afraid that the reviewers are not convinced of the conclusiveness and potential clinical impact of the findings. Since clear and conclusive insights into a novel clinically relevant observation are key for publication in EMBO Molecular Medicine, and together with the fact that we only accept papers that receive enthusiastic support upon initial review, I regret to say that we cannot offer to consider the manuscript further.

I am very sorry to have to disappoint you on this occasion, and hope that this negative decision does not prevent you from considering EMBO Medicine for the publication of future studies.

Yours sincerely,

Jingyi Hou

Jingyi Hou
Editor
EMBO Molecular Medicine

***** Reviewer's comments *****

Referee #1 (Remarks for Author):

Kuo et al described pathological role of Pdia4, one of protein disulfide isomerase, in progression of diabetes. Authors invitro analysis especially molecular interaction study and drug discovery based on the 3D structure is remarkable. However, some points need to be confirmed.

1) Manuscript was somewhat redundant especially some data was shown as (data not shown). If not showing these, this reviewer recommended to delete sentences. If important statements, please show data.
2) What is the strain of Leprdb/m or db/db? Researchers often utilize db/db mice from BKS strain and it is completely different from B6 strain. If authors used BKS strain, resultant Leprdb/ x Pdia

deficient mice are mixed background and cannot compare with B6 mice. IN such case, authors must perform experiment using Leprdb/m mice as control.
3) Even though authors did not show the data, the evidence that STZ-did not induce diabetes was surprising. Would like to know detail.
4) Authors stated that Pdia4 tg mice did not show any diabetic phenotype in basal condition, however no GTT challenge data was availavle. OGTT and IPGTT should be performed in non-diabetic Pdia4TG mice compared to littermate control.
5) Authors stated that that induction of beclin1 is marker for autophagy, however this is just one of the markers and no evidence of autophagy (Autophagy. 2016;12(1):1-222. doi: 10.1080/15548627.2015.1100356.)
6) Pdia4 is responsible for Ndufs3 or p22phox translocation?
7) The anti-oxidative stress effects of GHTT is observed when evaluating plasma or urine?
8) Authors should analyze plasma glucagon levels as well.
9) Authors should analyze several typical beta cell differentiation markers (ex, PDX1 or Mafs) and death marker (ex, MST1 or JNK)
10) Figure 1A and H: show full get image for Pdia4, since curious for truncation or variant form of Pdia4
11) Modulate is weak verb and authors should state much clear meaning (page 9)
12) Figure2D Brdu labeling in B6 mice: looks like Pdia4+/+ > -/-.

Referee #2 (Comments on Novelty/Model System for Author):

Concerns are raised with appropriateness of the models used in this study. The validity of the findings are difficult to ascertain because of vagueness in the description of the background strains used in db/db and Pdia4 KO mice. The phenotype may be an artefact of different background strains.

Referee #2 (Remarks for Author):

In the manuscript with the title " Pdia4 regulates β-cell pathogenesis in diabetes: molecular mechanism and targeted therapy" Kuo et al used knockout and overexpression in mice to examine the role of Pdia4 in in beta cell pathogenesis and diabetes. The paper finds that beta cell expression of Pdia4 is induced by high nutrients in diabetes, that ablation of Pdia4 alleviates diabetes, islet destruction and ROS production in diabetic mice, and that overexpression had opposite effects. A Pdia4 inhibitor suppressed diabetic development in diabetic mice. While the findings are viewed as interesting and novel, concerns are raised with appropriateness of the models used in this study. The validity of the findings is difficult to assess because of the vague description of the background strains used for db/db and Pdia4 KO mice. The phenotype may be an artefact generated by the mixing of different background strains.

The introductory information ignores two papers that have examined Pdia4 in models of disordered glucose homeostasis (doi: 10.2337/db12-0701 and 10.1371/journal.pone.0179963) in which Pdia4 was found to be upregulated in islets from diabetic mice and proposed as a biomarker for metabolic syndrome.

The background strain of the db/db mice is not clear. db/db mice become diabetic on the C57BL/KsJ background. The experimental design would not be valid if Pdia4 KO mice on B6 background were bred with db/db mice on KsJ background. Body weight of animals should be reported.

The use the beta cell toxin STZ is not an appropriate model for the analysis of islet phenotypes.

The Pdia4 inhibitor studies are preliminary. The specificity and target tissue of the inhibitor are not established.

Referee #3 (Comments on Novelty/Model System for Author):

My major concern is that the authors have primarily utilized the db/db model, which is not a bad model, but has little relevance to human obesity and diabetes. The authors did do high fat feeding, but layered this upon STZ treatments. But, there was little characterization of this model. I say "unclear at this stage" because I think the authors need a more scholarly discussion of the importance and relevance of this work to human disease and better justification for the selection of these models. I'm not saying they need to do all new studies in a different model (that's too much to ask), just that they need a better discussion and justification.

Referee #3 (Remarks for Author):

The manuscript by Kuo et al. describes extensive phenotyping studies of a factor, Pdia4, in glucose homeostasis in mainly an obesity model of diabetes. The authors show enrichment of Pdia4, a protein disulfide isomerase, in β-cells of mice, and that deletion of Pdia4, either globally or in β-cells results in protection from dysglycemia in the db/db model, presumably as a result of reductions in oxidative stress. Overexpression of Pdia4 in islets results in accelerated diabetes development and failure to accrue islet mass. Pdia4 was shown to interact with Ndufs3 and p22phox in a sequence-specific manner, and an inhibitor of Pdia4 protects against dysglycemia in db/db mice. Overall, this is an interesting study with extensive characterization of animals and copious phenotypic data. The findings are interesting, though the relationship to human disease is by-and-large lacking (as the models studies are all on leptinR-defective strains with no distinct studies relating to human disease). Nevertheless, the studies are interesting for results they represent and do raise the potential for Pdia4 to serve as a target in preventing obesity-related diabetes. As noted, strengths include the extensive phenotyping of the animals studied and the identification of a previously unrecognized factor in β-cell biology. Weaknesses include lack of relevance to typical human disease, some experiments lacking controls, and an overall uncertainty related to mechanisms that still prevail despite the interaction studies. Specific concerns and points for potential revision are noted below:

1. As presented, the bigger picture seems to be lacking. From the standpoint of oxidative stress, there is no doubt that it is important in obesity-induced diabetes, so that is not a new finding in this study. Indeed, several studies have pointed to the effect of reducing oxidative stress on the db/db model on glycemic outcomes (e.g. studies by Robertson and colleagues on Gpx1; studies on the effects of pioglitazone and other ROS-reducing drugs, etc.-none of which seemed to be referenced). In the context of islet biology, the potential role of Pdia4 is new, and the authors missed a chance to contextualize this finding mechanistically. The interaction studies with Ndufs3 and p22phox have a lot of potential, yet precisely how this factor functions in ROS generation by these proteins is lacking-is the interaction, per se, consequential? Have determined the interacting motifs and having performed extensive studies in MIN6 cells (which represent a non-ideal model of β cells), it is surprising that the authors did not do mutational studies in either Pdia4 and Ndufs3 and p22phox to show that these interactions are crucial for ROS generation. Similarly, the studies of GHTT are dissatisfying, as it is unclear if/how "inhibition" of Pdia4 affects these interactions.

2. To follow up on #1 above, the authors seemed to run out of steam after the extensive Results section, and what is presented in the Discussion is simply a restatement of the Results. Again, no discussion of the findings of this study in the context of what is really new to the field. A more scholarly discussion and even model figure showing specifically the "take home" message would have been welcome. This section needs a complete re-write.

3. In Fig. 1E, immunostaining data are not convincing. Immunostaining over background is not clear. Why did the authors not perform IF as in other figures (e.g. 1B)?

4. Significance of Pdia4 levels in the serum in Fig. 1G are not clear. It is hard to imagine that if Pdia4 is specific to β-cells that levels this high could be emanating from β-cells. If these data are correct, then likely they are emerging from another cell type. Also, the statement that Pdia4 levels could serve as a "useful biomarker" for diabetes is unjustified (how could they be any more useful than blood glucose levels?), as these data alone cannot verify that claim (levels in other disease states or states of inflammation should be verified). That statement should therefore be removed.

5. STZ studies of Pdia4-/- mice on the B6 background should either be presented or not, and should not have the extensive description for data not shown (p. 5).

6. Significance of the Vit C data (Fig. S2E) are unclear. None of the doses had an effect, raising the possibility that the Vit. C preparation/doses were inadequate. A positive control should be provided in these studies, or they should probably be removed for their lack of significance.

7. In the phenotypic characterization of the islets, the authors should consider studying the possibility that β-cells are de-differentiating in the db/db mice (as shown by the group of Accili and colleagues). Immunostaining of control and KOs for ALDH1A3 should be considered.

8. The β-cell specific knockouts are not well presented. The authors state that they used an Ins2-Cre driver, but this is not described in the methods section, nor is the expression of the Ins2-Cre line characterized. Ins2 is known to be expressed in the brain (unlike Ins1), and this can have profound effects on glucose homeostasis. No data are shown on specificity of the knockout or even if knockout was efficiently achieved in β-cells. The use of the term "clinical parameters" on page 7 is inappropriate for a preclinical model. A notable lacking control in these studies is the Cre+ controls- these should be included to ensure that the effect is not a Cre effect.

9. The transgenic studies are interesting, but could also represent the effect of overexpression of a protein in β cells at pharmacologic levels. The authors state that high levels of transgenic Pdia4 were expressed in islets, but these data are nowhere to be seen....protein levels should be shown along with specificity to ensure that other tissues do not account for the phenotype.

10. The GHTT studies need either more detailed description or should be removed. I understand that the authors are trying to show therapeutic relevance, but in reality these studies are only presented in a cursory fashion and specificity and mechanism are lacking. Notably, it is unclear how the activity of Pdia4 was specifically tested-it is unclear what the insulin turbidity assay is and how specific that is for Pdia4. Did the compound interfere with interaction with Ndufs3 and p22phox? As is, the description is unclear. Also, IC50 should be expressed in nM or µM and the assay should be described in more detail. Levels of the drug should be measured in circulation (PK parameters). How was the compound synthesized? Details are missing in the methods section.

11. As stated above, the significance of this work to human disease is not well addressed. There

are limited data with human tissues, and models utilized in this study (db/db) have limited relevance to human diabetes. This, moreover, is not at all discussed in the Discussion (which as noted above needs a re-write).

Minor:

12. On page 9, the authors state that "Amazingly, proteomic analysis.....". It is unclear why this shoud be amazing.

13. Supplemental figure quality is poor

As a service to authors, EMBO provides authors with the possibility to transfer a manuscript that one journal cannot offer to publish to another EMBO publication. The full manuscript and if applicable, reviewers reports are automatically sent to the receiving journal to allow for fast handling and a prompt decision on your manuscript. For more details of this service, and to transfer your manuscript to another EMBO title please click on Link Not Available

Please do not share this URL as it will give anyone who clicks it access to your account.

Point-to-Point Responses

First of all, the authors would like to thank Reviewers and the Editor for the constructive comments and suggestions. We would also like to thank the Editor for providing the opportunity to have our manuscript re-evaluated. We have addressed all of the Reviewers' comments for the previous manuscript (EMM-2019-11668) in the point-by-point responses below.

Our key modifications and improvements are summarized below:

1. Regarding the adequacy of mouse models: We first deleted the data on STZ-treated mouse model whose beta cells were impaired. Next, we improved our explanation of the mouse strains used in the study. For clarity, we modified the description of our breeding strategy in the Methods of the revised Supplementary Information to give more details. In short, our genetically modified mice were initially bred from B6 to BKS (Jax No. 000662) and $Lepr^{db/db}$ BKS background (Jax No. 000697) to decrease the "mixed background" problem. We thank the reviewer for pointing out that the use of B6 mice as a control was not adequate. In the revised study, we adopted BKS mice as the control and accordingly re-conducted all the experiments in genetically modified $Lepr^{db/db}$ mice on the BKS background in comparison with WT BKS mice (Figures 1E, 2B-2F, 3, 4, 5, S2, S4, S5, and S6). Please note that these are a misty gene-free $Lepr^{db/db}$ mice from the Jackson Lab and thus no $Lepr^{db/m}$ mice were generated in the breeding strategy (https://www.jax.org/strain/000697). As a result, the conclusions were the same. Finally, regarding the justification of the selection of mouse models of diabetes, we used $Lepr^{db/db}$ mice, as a first model of diabetes, and B6 mice fed with a high fat diet (HFD), as a second model of diabetes, in the revised manuscript. The reasons that we selected both models of diabetes were: (1) No perfect mouse models of diabetes exist and $Lepr^{db/db}$ mice are the most often used model of diabetes in the literature; (2) The HFD-treated mouse model of diabetes resembles human type 2 diabetes more closely than $Lepr^{db/db}$ mice in terms of disease etiology; and (3) Both models can complement each other and strengthen the conclusions. Since STZ impairs beta cells and may complicate the interpretation of the action of Pdia4, we deleted the data on the mice treated with STZ (Figure S5 of the original manuscript). However it is worth noting that use of $Lepr^{db/db}$ mice and HFD-treated B6 mouse models resulted in the same conclusions as the STZ-treated model that we deleted from the revised manuscript. Overall, the data clearly manifest the importance of Pdia4 in beta cell pathology and diabetes. Again, these mouse models are commonly used as animal models of diabetes in research of etiology and therapies for human diabetes.

2. Regarding improvement in the elucidation of the mechanism of action: On top of Beclin1, we have provided one extra marker of autophagy, LC3, to support the notion that Pdia4 promoted cell death in beta cells via ROS, and autophagy can be implicated in this cell death (Figure 4B). However, JNK seemed not to be implicated in beta cell death (Figure S9A). We also checked the markers of dedifferentiation (Figure S9B) and cell differentiation (Figure S9C). We found that Pdia4 promoted cell death in beta cells in a manner that involved autophagy (Figure 4) and dedifferentiation (A1dh1a3, Figure S9B) but not cell differentiation (PDX1 and MafA, Figure S9C). We also completed mechanistic studies of 2-β-D-glucopyranosyloxy1-hydroxytrideca 5,7,9,11-tetrayne (GHTT) in $Lepr^{db/db}$ mice (Figures 8 and S8). The data showed that Pdia4 inhibition

completely phenocopied the Pdia4 deficiency in reduced beta cell death and diabetes. Using the overall data obtained using genetic and chemical approaches, we concluded that Pdia4 acted through the interaction of Ndufs3 and $p22^{phox}$, key regulators of the electron transport complex 1 and Nox, leading to increased ROS and, consequently, cell death in beta cells and diabetes.

3. Regarding the pharmacological study of the Pdia4 inhibitor, GHTT: We completed a full study of GHTT in *Lepr*$^{db/db}$ mice (Figures 8 and S8). Briefly, we provided evidence about the immunohistochemical staining of mouse islets and pre-clinical parameters in *Lepr*$^{db/db}$ mice fed with GHTT. We also showed the mechanism by which GHTT inhibited the interaction between Pdia4 and Ndufs3 or $p22^{phox}$, leading to reduction of ROS production.

4. We have re-written a section of the discussion including discussion of the bigger picture of Pdia4 in the context of oxidative stress during diabetes, particularly the position of Pdia4 in ROS production, beta cell pathology, and diabetes. In addition, we have discussed the unique role Pdia4 in cell death and dedifferentiation but not proliferation in beta cell failure and diabetes at the molecular level. Accordingly, we have also added eight new references (42-44, 46, 47, and 54-56) to the revised manuscript.

5. In line with the modifications stated above, we have deleted the data (Figures 7E, 8D, S5) from the original manuscript, added new data (Figures 8C, 8D, 8F, 8G, S5, S8 and S9) to the revised Supplementary Information, and modified the data (Figures 1E, 1H, 2B-2F, 3, 4A-4D, 5, 7E, 8, S4, S6) in the revised manuscript.

Thank you very much for taking the time to re-evaluate our manuscript.

Referee #1 (Remarks for Author):

Kuo et al described pathological role of Pdia4, one of protein disulfide isomerase, in progression of diabetes. Authors in vitro analysis especially molecular interaction study and drug discovery based on the 3D structure is remarkable. However, some points need to be confirmed.

(1) Manuscript was somewhat redundant especially some data was shown as (data not shown). If not showing these, this reviewer recommended to delete sentences. If important statements, please show data.

Response: We thank the reviewer for the advice. We have deleted the data on STZ-treated mice from the revised manuscript because some reviewers thought that this model was not ideal. Instead, we have added data on high fat diet-induced diabetes in B6 mice, which highly resembles patients with human type 2 diabetes (Figure S5). We show the STZ data below for your reference.

Wild-type (WT) and *Pdia4*$^{-/-}$ mice on B6 background were used to investigate the role of Pdia4 in β-cell pathogenesis and diabetes. WT and *Pdia4*$^{-/-}$ B6 mice did not develop diabetes (Figure P1). However, following STZ treatment, WT B6 mice developed severe diabetes with fasting blood glucose (FBG) above 200 mg/dL whereas *Pdia4*$^{-/-}$ B6 mice developed mild diabetes with FBG of 126 to 200 mg/dL (FBG, Figure P1A). Furthermore, WT B6 mice had higher postprandial blood glucose (PBG, Figure P1A) and islet atrophy than *Pdia4*$^{-/-}$ B6 mice post STZ

treatment (αINS, Figure P1B). The severity of the islet atrophy was also associated with ROS content in islets (DHE, Figure P1B) and blood (Figure P1C).

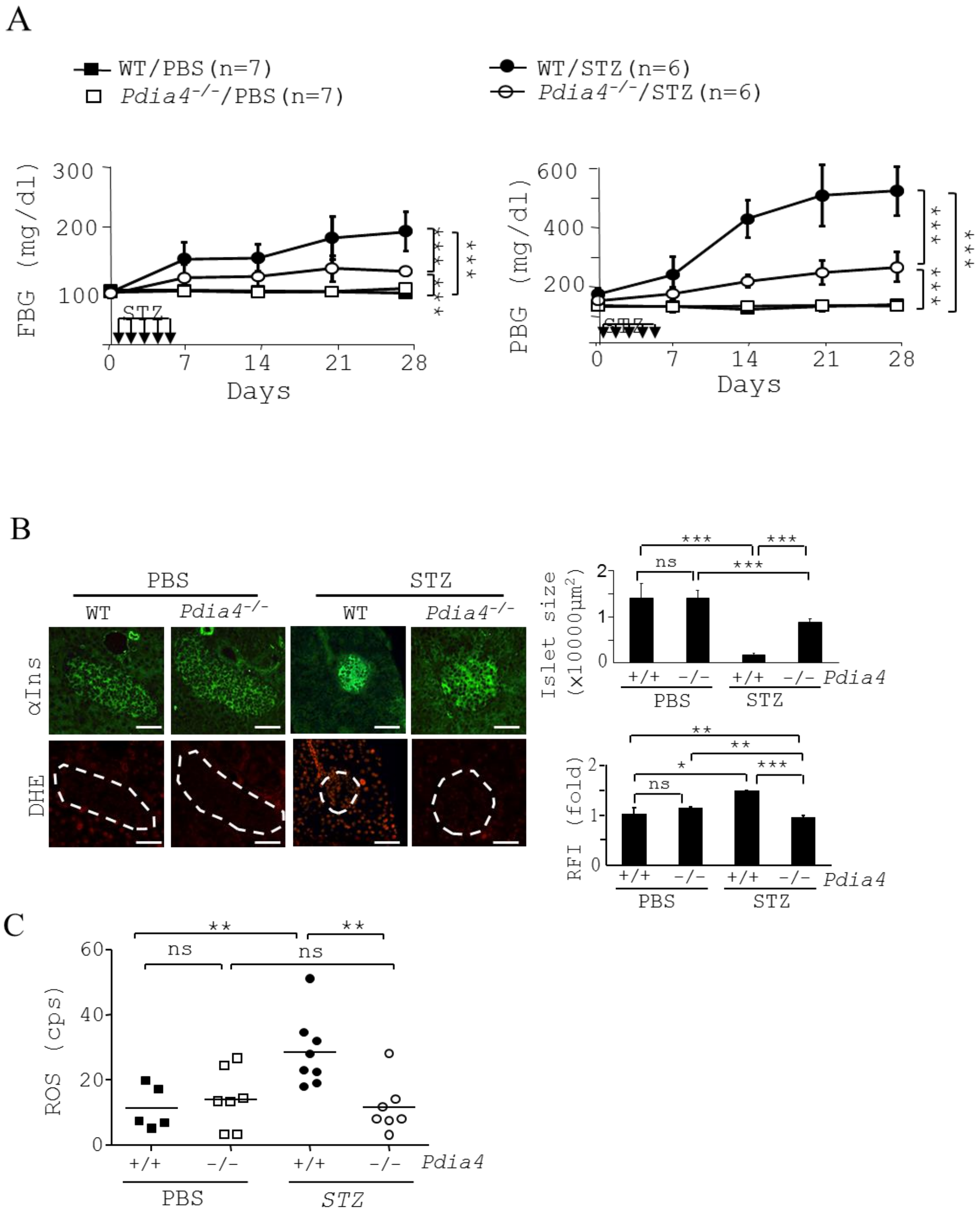

Figure P1. Pdia4 deficiency alleviates diabetes development, decreases islet atrophy, and reduces ROS production in STZ-treated B6 mice.
(A) FBG and PBG of WT and *Pdia4*$^{-/-}$ B6 mice receiving 5 doses of STZ and PBS were measured using a glucometer from day 0 to 28. (B) The pancreata were removed from the same mice (A) at sacrifice and stained with anti-insulin antibody (αIns) and secondary antibody or DHE (left). Islet area (μm^2) and relative fluorescence intensity (RFI) were quantified (right). Scale bar: 100 μm. (C) Serum ROS of the mice (A) were determined

on day 28. Data from three experiments (A-C) are presented as the mean ± SE. The number of mice (n) is indicated in parentheses.

2) What is the strain of Leprdb/m or db/db? Researchers often utilize db/db mice from BKS strain and it is completely different from B6 strain. If authors used BKS strain, resultant Leprdb/ x Pdia deficient mice are mixed background and cannot compare with B6 mice. IN such case, authors must perform experiment using Leprdb/m mice as control.
Response: We used db/db (*Lepr*$^{db/db}$ BKS) strain in our experiment. For clarity, we have provided the details about every mouse strain in the Methods of the revised Supplementary Information (page 3). Briefly, we bred our B6 mice to BKS background (Jackson Laboratory stock No. 000662). The mice were then crossed with B6.BKS(D)-*Lepr*^{db}/J mice (Jackson Laboratory stock No. 000697) in order to obtain *Lepr*$^{db/db}$ mice on the BKS background. As Reviewer pointed out that B6 mice were not good control mice for *Lepr*$^{db/db}$ mice on the BKS background, we re-conducted the mouse experiments and compared the genetically modified *Lepr*$^{db/db}$ mice with BKS control mice (except the high-fat diet-induced B6 mice for diabetes) in this revised manuscript. Of note, the data on the genetically modified *Lepr*$^{db/db}$ mice and B6 mice, fed with a high fed diet, showed similar results and conclusions.

3) Even though authors did not show the data, the evidence that STZ-did not induce diabetes was surprising. Would like to know detail.
Response: For the interest of the reviewer, we have shown the data on STZ mice in the above response (Figure P1). Since STZ could induce beta cell death via ROS, Pdia4 deficiency protected against diabetes in STZ-treated B6 mice.
However, we have deleted the data on the STZ mice in the revised manuscript because one reviewer thought it is less relevant to human diabetes. We instead added the data on high fat diet-induce diabetes in B6 mice (Figure S5), a more relevant mouse model to human diabetes, to this revised manuscript.

4) Authors stated that Pdia4 tg mice did not show any diabetic phenotype in basal condition, however no GTT challenge data was availavle. OGTT and IPGTT should be performed in non-diabetic Pdia4TG mice compared to littermate control.
Response: The OGTT data for Pdia4 transgenic mice is shown in Figure S6C of the revised manuscript.

5) Authors stated that that induction of beclin1 is marker for autophagy, however this is just one of the markers and no evidence of autophagy (Autophagy. 2016;12(1):1-222. doi: 10.1080/15548627.2015.1100356.)
Response: The reviewer is right about there being tens of molecules implicated in different steps (nucleation, elongation and maturation) of autophagy. Thus, we selected two key players as representative markers of this process. Beclin 1, an early nucleation regulator of autophagy, was up-regulated in the islet cells by Pdia4. We also showed that Pdia4 increased the expression of LC3-II, a player in the elongation step of this process in Figure 4B of this revised manuscript. Both pieces of data provided evidence that Pdia4 reduced islet cell death in correlation to the first two steps of the formation of autophagy based on markers of autophagy. Of

note, this manuscript emphasizes the novel discovery of Pdia4 in beta cell pathology and diabetes via ROS-generating pathways.

6) Pdia4 is responsible for Ndufs3 or p22phox translocation?
Response: Based on the literature (doi:10.1038/sj.cdd.4402004 and doi.org/10.1016/j.freeradbiomed.2014.07.046), Ndufs3 or p22^{phox} are transported from the cytosol to the mitochondrion and membrane of cell, respectively. In our studies, no difference was observed in the level of mitochondrial Ndufs3 or membrane p22^{phox} in WT Min6 cells and Min6 cells with Pdia4 knockdown (GK vs KD, Figure 7D). The data argue against the implication of Pdia4 in the translocation of Ndufs3 or membrane p22^{phox}.

7) The anti-oxidative stress effects of GHTT is observed when evaluating plasma or urine?
Response: We have added the effect of GHTT on serum ROS (Figure S8C) and cellular ROS (Figure 8C). We did not test urine ROS.

8) Authors should analyze plasma glucagon levels as well.
Response: We compared the plasma glucagon in *Lepr*$^{db/db}$, *Pdia4*$^{-/-}$ *Lepr*$^{db/db}$, and *Pdia4*$^{tg/tg}$ *Lepr*$^{db/db}$ mice in comparison with BKS control mice. We found that *Lepr*$^{db/db}$ and *Pdia4*$^{tg/tg}$ *Lepr*$^{db/db}$ mice and had a higher level of the plasma glucagon than *Pdia4*$^{-/-}$ *Lepr*$^{db/db}$ and BKS mice post meal (Figure P2). The data on the plasma glucagon might reflect that under hyperglycemia, *Lepr*$^{db/db}$ and *Pdia4*$^{tg/tg}$ *Lepr*$^{db/db}$ mice had more dysfunctional beta cells than *Pdia4*$^{-/-}$ *Lepr*$^{db/db}$ and BKS mice.

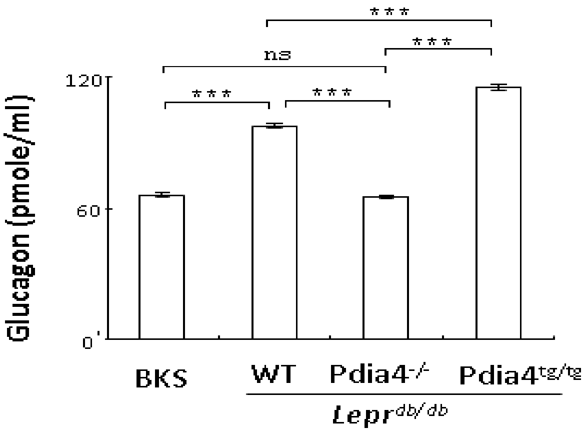

Figure P2. Plasma glucagon levels in different mouse strains. *Lepr*$^{db/db}$ (WT), *Pdia4*$^{-/-}$, and *Pdia4*$^{tg/tg}$ mice on the *Lepr*$^{db/db}$ background and BKS mice were bled and their postprandial plasma were collected and measured for glucagon.

9) Authors should analyze several typical beta cell differentiation markers (ex, PDX1 or Mafs) and death marker (ex, MST1 or JNK)
Response: We thank the reviewer for the advice. Our data show that Pdia4 did not affect beta cell proliferation but increased beta cell death (Figure 2D). Consistently, we found that Pdia4 did not affect the expression level of PDX1 and MafA (Figure S9B), implying that Pdia4 did not influence proliferation and differentiation of beta cells. In contrast, we provided evidence that this cell death could be related to Beclin 1 and LC3, two key markers of autophagy in mammalian cells (Figure 4B).

Consistently, we found that Pdia4 did not change the expression and activation (phosphorylation) of JNK in islet cells (Figure S9A). Although we did not test MST1, an upstream regulator of PDX1 and JNK (doi:10.1038/nm.3482), the overall data demonstrated that Pdia4 regulated cell death (Figure 4) and expression of Aldh1a3 (Figure S9B) but not differentiation (PDX1 and MafA, Figure S9C) or proliferation (BrdU, Figure 2D) in beta cells. This regulation involved autophagy and dedifferentiation though we did not go into the details about the mechanism of Pdia4 in the autophagy of beta cells (Figure 4). Instead, we focus this study on the functional and mechanistic studies of Pdia4 in beta cell pathology and diabetes via regulation of the ROS machinery. We will address the significance of Pdia4 in autophagy in our next manuscript.

10) Figure 1A and H: show full get image for Pdia4, since curious for truncation or variant form of Pdia4

Response: We have provided the full images in Figure 1A. The full image of Pdia4 (top, Figure P3A) and actin (bottom, Figure P3A) are shown. Please note that the antibody against Pdia4 in Figure P3A and Figure 1A was rabbit polyclonal antiserum. The data seemed to have some extra bands, implying the existence of Pdia4 isoforms or noise from rabbit polyclonal serum. In contrast, we conducted another immunoblotting analysis of the same lysates using mouse monoclonal anti-Pdia4 antibody (Figure P3B). The data clearly showed a specific band corresponding to Pdia4 (Figure P3B). Thus, our data argue against the existence of Pdia4 isoforms in various mouse tissues.
Figures P3C-P3H show full images of the immunoblot using the indicated antibodies (Figure 1H). You can see a specific band of Pdia4 in different compartments of Min6 cells, too. Our data are consistent with the fact that no Pdia4 isoforms are reported.

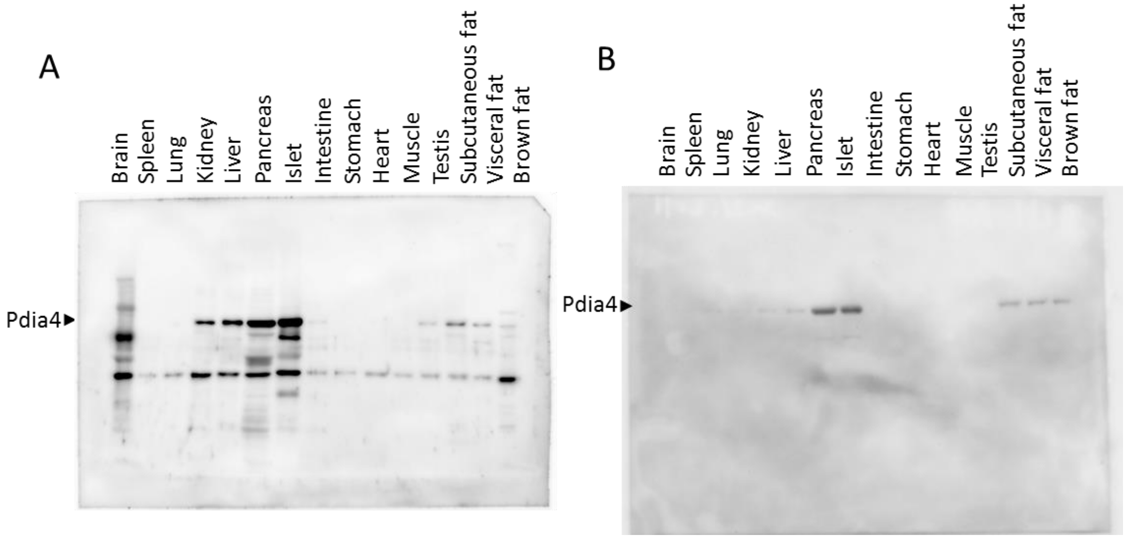

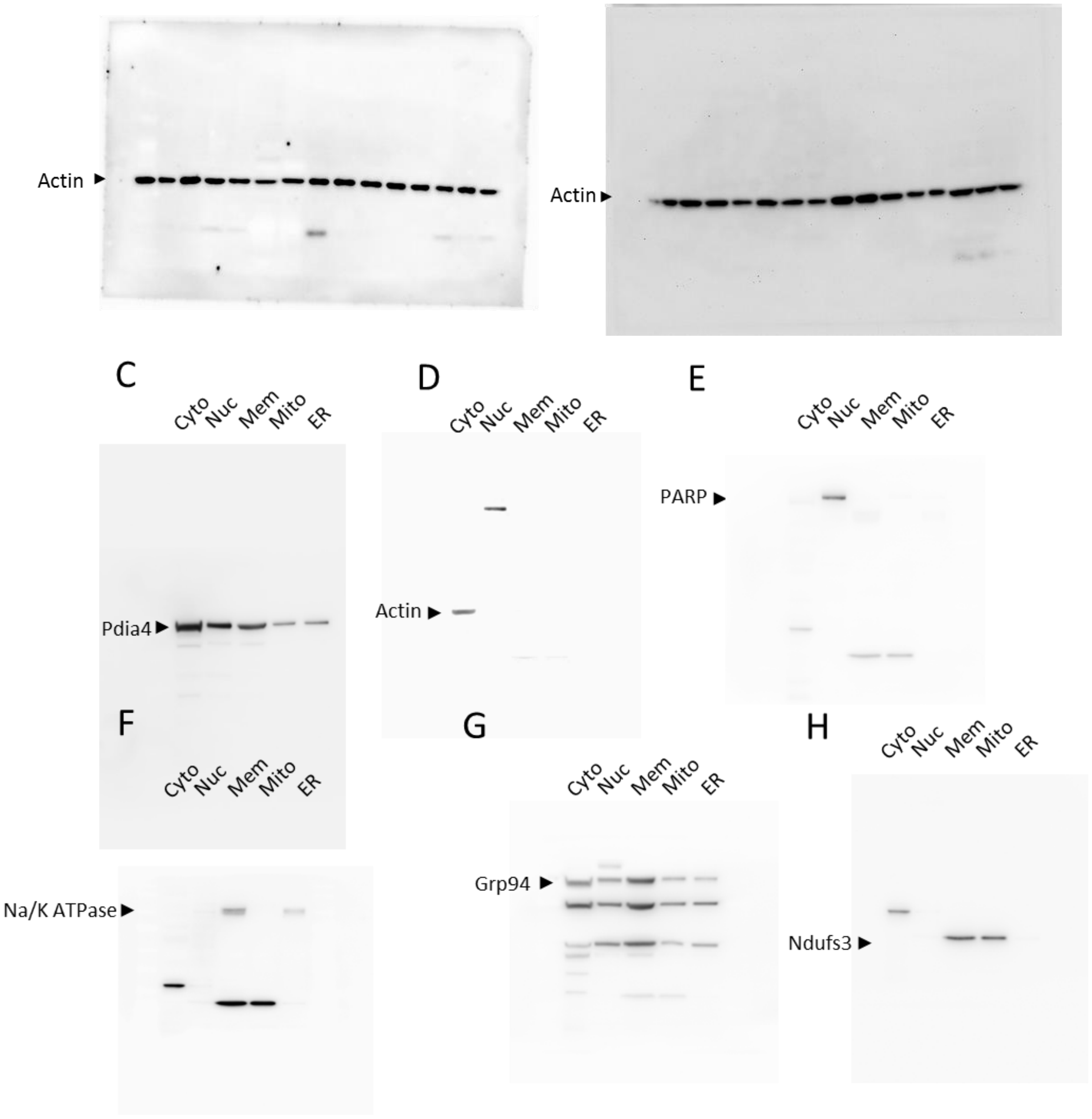

Figure P3. Immunoblot of different mouse organs using the indicated antibodies. (A) Full image of Figure 1A using rabbit polyclonal anti-Pdia4 antibody. Total lysates of different mouse organs underwent immunoblotting analysis using mouse monoclonal antibody against actin (bottom). The same blot as (A) was then hybridized with rabbit polyclonal antibody against Pdia4 (top). (B) The full image of the membrane containing the same lysates as (A) was hybridized with mouse monoclonal antibody against Pdia4 (top) and actin (bottom). (C-H) The full image of Figure 1H. Immunoblotting analysis of Pdia4 and markers in the cytosolic (Cyto), nuclear (Nuc), membrane (Mem), mitochondrial (Mito), and ER compartments of Ming 6 cells using the indicated antibodies. The same membrane was blotted with mouse monoclonal anti-Pdia4 antibody (C), followed by anti-actin (D), anti-PARP (E), anti-Na/K ATPase (F), anti-Grp94 (G), and anti-Ndufs3 (H) antibodies.

11) Modulate is weak verb and authors should state much clear meaning (page 9)
Response: We thank the reviewer for the advice. We use the verb "modulate" to briefly sum up. We explain the detailed mechanism through which Pdia4 modulates ROS production, cell death and cell dysfunction in β-cells through the regulation of Nox and ETC activities in Figure 8G and the last paragraph of the Results section (pages 11-12). We also discuss this mechanism in the first three paragraphs of the Discussion section (pages 12-13).

12) Figure2D Brdu labeling in B6 mice: looks like Pdia4+/+ > -/-.
Response: We changed the B6 control mice with BKS mice. The reviewer is right that $BrdU^{+}$ signal in WT BKS mice was higher than that in $Pdia4^{-/-}$ BKS mice. However, the data were not statistically significant.

Referee #2 (Comments on Novelty/Model System for Author):

Concerns are raised with appropriateness of the models used in this study. The validity of the findings are difficult to ascertain because of vagueness in the description of the background strains used in db/db and Pdia4 KO mice. The phenotype may be an artefact of different background strains.
Response: Since the mouse strains used in the study were complicated, for clarity, we have provided the details about every mouse strain in the Methods of the revised Supplementary Information (page 3). Briefly, we bred our B6 mice to BKS background (Jackson Laboratory stock No. 000662). The mice were then crossed with B6.BKS(D)-*$Lepr^{db}$*/J mice (Jackson Laboratory stock No. 000697) in order to obtain *$Lepr^{db/db}$* mice on the BKS background. We made the mistake of using B6 mice as control mice in the original manuscript. As one reviewer pointed out that B6 mice were not good control mice for *$Lepr^{db/db}$* mice on the BKS background, we re-conducted the mice experiments and compared the genetically modified *$Lepr^{db/db}$* mice with BKS control mice (except the high-fat diet-induced B6 mice for diabetes) in this revised manuscript.
In this revised version, all our genetically modified mice are on BKS background and we compared the genetically modified *$Lepr^{db/db}$* mice with BKS control mice. Of note, the data on the genetically modified *$Lepr^{db/db}$* mice and B6 mice, fed with a high fat diet, showed similar results as the data on *$Lepr^{db/db}$* mice, treated with the Pdia4 inhibitor GHTT. Altogether, genetic and pharmacological data support that the phenotype of our mice comes from Pdia4 but not the artefact of different background strains.

Referee #2 (Remarks for Author):

In the manuscript with the title " Pdia4 regulates β-cell pathogenesis in diabetes: molecular mechanism and targeted therapy" Kuo et al used knockout and overexpression in mice to examine the role of Pdia4 in in beta cell pathogenesis and diabetes. The paper finds that beta cell expression of Pdia4 is induced by high nutrients in diabetes, that ablation of Pdia4 alleviates diabetes, islet destruction and ROS production in diabetic

mice, and that overexpression had opposite effects. A Pdia4 inhibitor suppressed diabetic development in diabetic mice. While the findings are viewed as interesting and novel, concerns are raised with appropriateness of the models used in this study. The validity of the findings is difficult to assess because of the vague description of the background strains used for db/db and Pdia4 KO mice. The phenotype may be an artefact generated by the mixing of different background strains.
Response: Since the mouse strains used in the study were complicated, for clarity, we have provided the details about every mouse strain in the Methods of the revised Supplementary Information (page 3). Briefly, we bred our B6 mice to BKS background (Jackson Laboratory stock No. 000662). The mice were then crossed with B6.BKS(D)-*Lepr*^{db}/J mice (Jackson Laboratory stock No. 000697) in order to obtain *Lepr*$^{db/db}$ mice on the BKS background. We made the mistake of using B6 mice as control mice in the original manuscript. As one reviewer pointed out that B6 mice were not good control mice for *Lepr*$^{db/db}$ mice on the BKS background, we re-conducted the mouse experiments and compared the genetically modified *Lepr*$^{db/db}$ mice with BKS control mice (except the high-fat diet-induced B6 mice for diabetes) in this revised manuscript.
In this revised version, all our genetically modified mice are on BKS background and we compared the genetically modified *Lepr*$^{db/db}$ mice with BKS control mice. Of note, the data on the genetically modified *Lepr*$^{db/db}$ mice and B6 mice, fed with a high fat diet, showed similar results as the data on *Lepr*$^{db/db}$ mice, treated with the Pdia4 inhibitor GHTT. Altogether, genetic and pharmacological data support that the phenotype of our mice comes from Pdia4 but not the artefact of different background strains.

The introductory information ignores two papers that have examined Pdia4 in models of disordered glucose homeostasis (doi: 10.2337/db12-0701 and 10.1371/journal.pone.0179963) in which Pdia4 was found to be upregulated in islets from diabetic mice and proposed as a biomarker for metabolic syndrome.
Response: We thank the reviewer for this comment. We have added both to our revised manuscript (references #54 and # 55).

The background strain of the db/db mice is not clear. db/db mice become diabetic on the C57BL/KsJ background. The experimental design would not be valid if Pdia4 KO mice on B6 background were bred with db/db mice on KsJ background. Body weight of animals should be reported.
Response: Since the mouse strains used in the study were complicated, for clarity, we have provided the details about every mouse strain in the Methods of the revised Supplementary Information (page 3). Briefly, we bred our B6 mice to BKS background (Jackson Laboratory stock No. 000662). The mice were then crossed with B6.BKS(D)-*Lepr*^{db}/J mice (Jackson Laboratory stock No. 000697) in order to obtain *Lepr*$^{db/db}$ mice on the BKS background. As one reviewer pointed out that B6 mice were not good control mice for *Lepr*$^{db/db}$ mice on the BKS background, we compared the genetically modified *Lepr*$^{db/db}$ mice with BKS control mice (except the high-fat diet-induced B6 mice for diabetes) in this revised manuscript.

In this revised version, all our genetically modified mice are on BKS background and we compared the genetically modified *Lepr*$^{db/db}$ mice with BKS control mice. Body weight of different mouse strains is shown below (Figure P4). We found that BKS mice aged 8 weeks had an average weight of ~18 g and their body weight slightly increased to 25 g at the age of 24 weeks (Figure P4). No difference in body weight was seen in WT, *Pdia4*$^{-/-}$ and *Pdia4*$^{tg/tg}$ BKS mice. In sharp contrast, *Lepr*$^{db/db}$ mice aged 8 weeks had an average weight of > 30 g and their body weight increased to > 49 g at the age of 24 weeks. Of note, *Pdia4*$^{-/-}$ *Lepr*$^{db/db}$ mice gained more body weight than *Lepr*$^{db/db}$ and *Pdia4*$^{tg/tg}$ *Lepr*$^{db/db}$ mice over time, probably because they were able to control blood sugar with sufficient insulin and had "insulin side effect", a phenomenon of body weight gain by insulin.

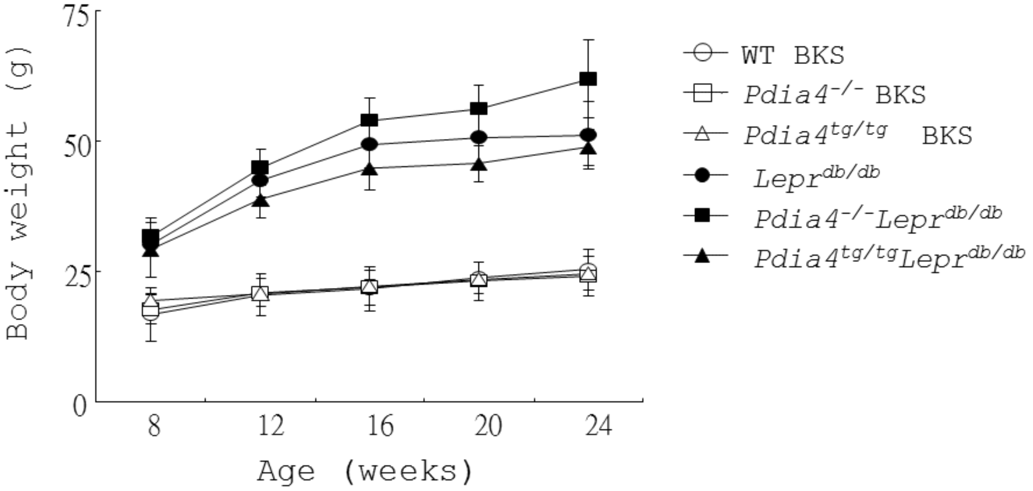

Figure P4. Body weight change in genetically modified BKS and *Lepr*$^{db/db}$ mice. WT, *Pdia4*$^{-/-}$, and *Pdia4*$^{tg/tg}$ mice on the BKS and *Lepr*$^{db/db}$ backgrounds were monitored weekly for their body weight from 8 to 24 weeks. Body weight change in 6 lines of mice is indicated.

The use the beta cell toxin STZ is not an appropriate model for the analysis of islet phenotypes.

Response: We agree with the reviewer and have deleted the data on the STZ mice in the revised manuscript. We have also added the data on high fat diet-induce diabetes in B6 mice, as a second mouse model of diabetes, which is more relevant to human diabetes (FigureS5).

The Pdia4 inhibitor studies are preliminary. The specificity and target tissue of the inhibitor are not established.

Response: We have examined the same pre-clinical parameters of diabetes in *Lepr*$^{db/db}$ mice, treated with the Pdia4 inhibitor GHTT, as those in genetically modified *Lepr*$^{db/db}$ mice. We have added the data on the impact of GHTT, the Pdia4 inhibitor, on cellular ROS (Figure 8C), interaction between Pdia4 and Ndufs3 and p22^{phox} (Figure 8D), insulin/ROS content in islets (Figure 8E), GTT (Figure S8A), HbA1c (Figure S8B), HOMA indices (Figure S8D) and serum ROS (Figure S8E).

Our data showed that Pdia4, but not Pdia1, Pdia3 and Pdia6, controlled ROS production via Ndufs3 and p22^{phox} (Figure S7C) and that Pdia4 was mainly expressed in islet cells (Figures 1A and 1B), implying that pancreatic islets should be the primary target of the Pdia4 inhibitor, GHTT.
We provided the *in vitro* specificity of GHTT for Pdia4 with other Pdi members (ia1, Pdia3 and Pdia6) based on the IC_{50} values. Pdia4 had a 2 times lower IC_{50} value for GHTT than other Pdi proteins (Table P1). The data suggested that GHTT had a reasonable specificity to Pdia4. Furthermore, GHTT did interrupted the interaction of Pdia4 with the Pdia4 partners *in vivo* (Figure 8D).

Table P1. IC_{50} values of Pdia4 and other Pdi members.

	Pdia1	Pdia3	**Pdia4**	Pdia6
**IC_{50} (μM)**	1046	671	**358**	1198

Referee #3 (Comments on Novelty/Model System for Author):

My major concern is that the authors have primarily utilized the db/db model, which is not a bad model, but has little relevance to human obesity and diabetes. The authors did do high fat feeding, but layered this upon STZ treatments. But, there was little characterization of this model. I say "unclear at this stage" because I think the authors need a more scholarly discussion of the importance and relevance of this work to human disease and better justification for the selection of these models. I'm not saying they need to do all new studies in a different model (that's too much to ask), just that they need a better discussion and justification.
Response: We thank the reviewer for valuable comments. Since no mouse models of diabetes are the same as human diabetes, we initially used *Lepr*$^{db/db}$ mice and B6 mice treated with high fat diet plus STZ. A combination of high fat diet plus STZ was used in the original manuscript because B6 mice easily develop obesity but not diabetes (need a longer time).
To better investigate the role of Pdia4 in knockout and transgenic mouse strains, in the revised manuscript, we took the reviewer's advice and used *Lepr*$^{db/db}$ mice on the BKS background (Figures 2, 3, S2, S3, and S4) and B6 mice fed with high fat diet for a longer time (Figure S5). *Lepr*$^{db/db}$ mice were used as the first animal model of diabetes. Complementarily, B6 mice fed with high fat diet served as the second animal model of diabetes to confirm the importance of Pdia4 in diabetes. Hopefully, both lines of evidence in mouse models of diabetes can better address the importance of Pdia4 in human diabetes. Collectively, the data on *Lepr*$^{db/db}$ mice and B6 mice showed similar results leading to the same conclusions. Although we have deleted the data on STZ mice in the revised manuscript, it is worthwhile to note that data obtained from the three models pointed to the same outcomes and conclusions. We have also discussed the selection of *Lepr*$^{db/db}$ mice and B6 mice, which complemented each other and revealed a novel function of Pdia4 in β-cell death and diabetes (first paragraph of the Discussion section, page 12).

Referee #3 (Remarks for Author):

The manuscript by Kuo et al. describes extensive phenotyping studies of a factor, Pdia4, in glucose homeostasis in mainly an obesity model of diabetes. The authors show enrichment of Pdia4, a protein disulfide isomerase, in β-cells of mice, and that deletion of Pdia4, either globally or in β-cells results in protection from dysglycemia in the db/db model, presumably as a result of reductions in oxidative stress. Overexpression of Pdia4 in islets results in accelerated diabetes development and failure to accrue islet mass. Pdia4 was shown to interact with Ndufs3 and p22phox in a sequence-specific manner, and an inhibitor of Pdia4 protects against dysglycemia in db/db mice. Overall, this is an interesting study with extensive characterization of animals and copious phenotypic data. The findings are interesting, though the relationship to human disease is by-and-large lacking (as the models studies are all on leptinR-defective strains with no distinct studies relating to human disease). Nevertheless, the studies are interesting for results they represent and do raise the potential for Pdia4 to serve as a target in preventing obesity-related diabetes. As noted, strengths include the extensive phenotyping of the animals studied and the identification of a previously unrecognized factor in β-cell biology. Weaknesses include lack of relevance to typical human disease, some experiments lacking controls, and an overall uncertainty related to mechanisms that still prevail despite the interaction studies. Specific concerns and points for potential revision are noted below:

1. As presented, the bigger picture seems to be lacking. From the standpoint of oxidative stress, there is no doubt that it is important in obesity-induced diabetes, so that is not a new finding in this study. Indeed, several studies have pointed to the effect of reducing oxidative stress on the db/db model on glycemic outcomes (e.g. studies by Robertson and colleagues on Gpx1; studies on the effects of pioglitazone and other ROS-reducing drugs, etc.-none of which seemed to be referenced). In the context of islet biology, the potential role of Pdia4 is new, and the authors missed a chance to contextualize this finding mechanistically. The interaction studies with Ndufs3 and p22phox have a lot of potential, yet precisely how this factor functions in ROS generation by these proteins is lacking-is the interaction, per se, consequential? Have determined the interacting motifs and having performed extensive studies in MIN6 cells (which represent a non-ideal model of β cells), it is surprising that the authors did not do mutational studies in either Pdia4 and Ndufs3 and p22phox to show that these interactions are crucial for ROS generation. Similarly, the studies of GHTT are dissatisfying, as it is unclear if/how "inhibition" of Pdia4 affects these interactions.
Response: We thank the reviewer for comprehensive evaluation and excellent suggestions. We have re-written the Discussion section of the revised manuscript by conceptualizing our new finding of the Pdia4/Ndufs and Pdia4/p22^{phox} pathways in the context of the oxidative stress (i.e., ROS) during diabetes in Figure 8G and discussed the importance of the novel finding of Pdia4/Ndufs and p22^{phox}/ROS generation pathways (first two paragraphs of the Discussion section). This work adds a new target and pathway to beta cell physiopathology and diabetes, which can be potentially used to treat and cure diabetes.

We have demonstrated the molecular interaction between Pdia4 and Ndufs3 and p22^{phox} in ROS production in beta cells (Figure 6). Moreover, a truncated version of Ndufs3 and p22^{phox} acted as a dominant-negative mutant to interfere with Pdia4-mediated ROS production (Figures 7B and 7C), suggesting that Ndufs3 and p22^{phox} were located downstream in the context of ROS production (Figure 7). Currently, primary beta cells are difficult to purify alive to a usable quantity. Therefore, we used Min6 cells, a murine beta cell line, which the reviewer argued, might not be the ideal cells (Figure 6, the revised manuscript). We also examined the role of Pdia4 in the human beta cell line, EndoC-betaH2 (J Clin Invest 124(5):2087-98). Our data showed that Pdia4 bound to Ndufs3 and p22^{phox} in human beta cells (Figure P5), similar to the data on Min6 cells. Both pieces of data support the finding that Pdia4 interacted with Ndufs3 and p22^{phox}, two key players in the ROS-generating pathways, and controlled ROS production in beta cells.
Molecules with point mutations might not interact and intervene with their endogenous partners. It was the case for Pdia4. We used over-expression of wild-type Pdia4, instead of Pdia4 with 3 CGHC mutations (Pdia4*), because Pdia4* failed to bind and interfere with Ndufs3 and p22^{phox}. Similarly, the truncated versions of Ndufs3 and p22^{phox} were to test their roles in ROS production (Figure 7). The data showed that both truncated molecules largely reduced Pdia4-mediated ROS production in Min6 cells. Complimentarily, we did test the effect of GHTT on the inhibition of Pdia4, interaction of Pdia4 with Ndufs3 and p22^{phox}, and ROS production. As a result, the Pdia4 inhibitor GHTT (Figure 8B) decreased this interaction (Figure 8D), ROS production in the cytoplasmic and mitochondrial compartments of beta cells (Figure 8C), and islet cells (Figure 8F). This is in good agreement with the literature reporting that Ndufs3 and p22^{phox} are known as key players that regulate ROS production in the mitochondria and cytosol of cells. The overall data suggest a causative relationship between the Pdia4/Ndufs3 and Pdia4/p22^{phox} axes and ROS production.

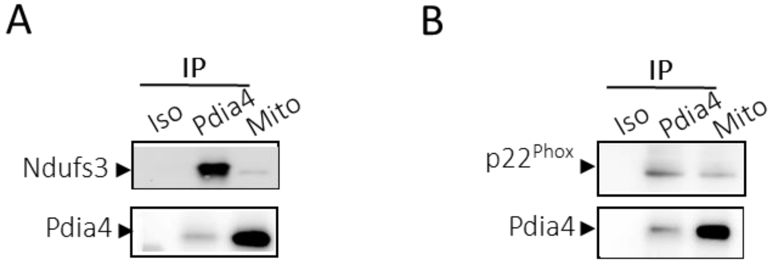

Figure P5. Pdia4 can bind Ndufs3 or p22^{phox} in human beta cells. Mitochondrial (Mito, A), and membrane (Mem, B) fractions of EndoC-betaH2 cells were precipitated with isotype (Iso) or anti-Pdia4 (Pdia4) antibody. The fractions and immunoprecipitates (IP) underwent immunoblotting analysis with the indicated antibodies.

2. To follow up on #1 above, the authors seemed to run out of steam after the extensive Results section, and what is presented in the Discussion is simply a restatement of the Results. Again, no discussion of the findings of this study in the context of what is really new to the field. A more scholarly discussion and even model figure showing specifically

the "take home" message would have been welcome. This section needs a complete re-write.

Response: We appreciate the reviewer's advice. We have re-written the whole Discussion section of the revised manuscript. First, we have outlined the bigger picture with regard to our new discovery of the Pdia4/ Ndufs3 and Pdia4/p22^{phox} cascades in the context of oxidative stress in beta cells (first two paragraphs of the Discussion section). Second, we have continued to discuss the relationship between ROS homeostasis and beta cell pathology, and diabetes in relation to Pdia4. We have stressed that targeting the Pdia4 pathway was better than ROS scavenger vitamin C in terms of diabetes therapy (second paragraph of the Discussion section). We also discussed the poorly studied dysfunction and demise of beta cells during diabetes. Pdia4 came out as a key player in the regulation of beta cell function and protection (third paragraph of the Discussion section). Next, we discussed the significance and molecular mechanism of Pdia4 in beta cell pathogenesis and diabetes (fourth paragraph of the Discussion section). In parallel, Pdia4 was identified as an ER chaperone. However, it was mainly expressed in beta cells and it was distributed everywhere in the cell compartments of beta cells (fifth paragraph of the Discussion section). Finally, we have discussed the uniqueness of Pdia4 in Pdi family in terms of beta cell death and diabetes

3. In Fig. 1E, immunostaining data are not convincing. Immunostaining over background is not clear. Why did the authors not perform IF as in other figures (e.g. 1B)?

Response: We repeated the data in Figure 1E using WT BKS and *Lepr*$^{db/db}$ mice. The fold-change of islet Pdia4 in diabetic *Lepr*$^{db/db}$ mice versus non-diabetic BKS mice was 2 fold. We did use other techniques such as immunohistochemical staining to compare the expression level of Pdia4 in non-diabetic and diabetic islets of *Lepr*$^{db/db}$ and *Lep*$^{ob/ob}$ mice (Figure 1F). Nevertheless, induction of Pdia4 expression was in diabetic islets was 2-4 fold compared to non-diabetic counterparts (Figures 1C, 1D, 1E and 1F).

4. Significance of Pdia4 levels in the serum in Fig. 1G are not clear. It is hard to imagine that if Pdia4 is specific to β-cells that levels this high could be emanating from β-cells. If these data are correct, then likely they are emerging from another cell type. Also, the statement that Pdia4 levels could serve as a "useful biomarker" for diabetes is unjustified (how could they be any more useful than blood glucose levels?), as these data alone cannot verify that claim (levels in other disease states or states of inflammation should be verified). That statement should therefore be removed.

Response: Figure 1G only showed that Pdia4 was secreted into serum in mice and humans. The serum level of Pdia4 was increased in diabetic mice and humans. This increased Pdia4 might not be attributed to release from dead beta cells because diabetic patients and mice had higher Pdia4 than non-diabetic counterparts in their lifetime. We argued that this increased serum Pdia4 came from other type of islet cells because Pdia4 was not expressed in pancreatic alpha cells (Figure 1B). Pdia4 was increased in diabetes in the serum of high fat diet-fed B6 mice (middle, Fig 1G) and patients with metabolic syndrome (reference # 55 in the revised manuscript). This publication stated that the level of Pdia4 increased over the period of disease

progression and concluded that Pdia4 was a potential marker for metabolic syndrome. Thus, it is likely that increased ROS elevated the protein level of Pdia4 in different compartments of beta cells including its release to serum.
We have changed "useful" into "potential" in the sentence (page 5), which is consistent with reference # 55, in the revised manuscript.

5. STZ studies of Pdia4-/- mice on the B6 background should either be presented or not, and should not have the extensive description for data not shown (p. 5).
Response: We thank the Reviewer for the advice. We have deleted the data on STZ-treated mice. Instead, we added the data on the high fat diet-induced diabetes in B6 mice (Figure S5). There is no "data not shown" in the revised manuscript.

6. Significance of the Vit C data (Fig. S2E) are unclear. None of the doses had an effect, raising the possibility that the Vit. C preparation/doses were inadequate. A positive control should be provided in these studies, or they should probably be removed for their lack of significance.
Response: Vitamin C is known as one of the strongest ROS scavengers. We are not able to find a positive control. Vitamin C used in the study was quality controlled by its purity and anti-oxidant activity. We compared vitamin C treatment with Pdia4 deficiency for diabetes treatment in an attempt to stress the different outcomes of ROS scavenging and inhibition of ROS production in vivo (Figure S2E).

7. In the phenotypic characterization of the islets, the authors should consider studying the possibility that β-cells are de-differentiating in the db/db mice (as shown by the group of Accili and colleagues). Immunostaining of control and KOs for ALDH1A3 should be considered.
Response: We thank the reviewer for the advice. Aldh1a3 has been reported to be a dedifferentiation marker of beta cells in diabetic mice. We tested its expression in aged BKS, $Lepr^{db/db}$, $Pdia4^{-/-}$ $Lepr^{db/db}$, and $Pdia4^{tg/tg}Lepr^{db/db}$ mice using a confocal microscope. We found that $Pdia4^{tg/tg}Lepr^{db/db}$ had a higher Aldh1a3 expression level in their pancreas than $Lepr^{db/db}$ mice (Figure S9B). In sharp contrast, similar to BKS control mice, no Aldh1a3 in the pancreatic islets of $Pdia4^{-/-}$ $Lepr^{db/db}$ mice was observed mice (Figure S9B). Our data indicated that Aldh1a3 was expressed in the islets of diabetic $Lepr^{db/db}$ mice and this expression was up-regulated in the islets of $Pdia4^{tg/tg}Lepr^{db/db}$ mice. However, Aldh1a3 was reduced in the islets of non-diabetic $Pdia4^{-/-}$ $Lepr^{db/db}$ mice and BKS mice (Figure S9B). The overall data suggested that Pdia4 was positively correlated to the expression of Aldh1a3.
Unexpectedly, we discovered that Pdia4 knockout abolished an $Aldh1a3^{+}$subset of failing beta cells (Figure S9B). The data are in good agreement with cell death and dysfunction cells in beta cells as shown by GSIS (Figures 2F and 3D), insulin content (Figure 3E), insulin granules (Figures 5A and 5C), mitochondrial parameters (Figures 5A and 5B) and cell death (Figures 4 and S9). However, the relationship between Pdia4 and islet dedifferentiation needs to be further ascertained.

8. The β-cell specific knockouts are not well presented. The authors state that they used

an Ins2-Cre driver, but this is not described in the methods section, nor is the expression of the Ins2-Cre line characterized. Ins2 is known to be expressed in the brain (unlike Ins1), and this can have profound effects on glucose homeostasis. No data are shown on specificity of the knockout or even if knockout was efficiently achieved in β-cells. The use of the term "clinical parameters" on page 7 is inappropriate for a preclinical model. A notable lacking control in these studies is the Cre+ controls-these should be included to ensure that the effect is not a Cre effect.
Response: We have described the experimental design in the Methods section of the revised Supplemental Information (page 3). Regarding the controversial issue reporting that Ins2 was expressed in mouse pancreas as well as brain based on IHC and in situ hybridization techniques, one recent paper demonstrated that Ins2-Cre mice revealed no Cre activity in mouse hypothalamic neurons and exhibited normal glucose tolerance and insulin secretion using knock-in technique of Cre in Ins2 locus (Scientific Reports 6:20438). We also found that In2-Cre mice had normal glucose tolerance and insulin secretion in our studies.
We have changed "clinical parameters" into "pre-clinical parameters" in the revised manuscript (page 6).

9. The transgenic studies are interesting, but could also represent the effect of overexpression of a protein in β cells at pharmacologic levels. The authors state that high levels of transgenic Pdia4 were expressed in islets, but these data are nowhere to be seen....protein levels should be shown along with specificity to ensure that other tissues do not account for the phenotype.
Response: The data are shown in Figure P6. The details of transgenic mice are described in Figure S5. *Pdia4*$^{tg/tg}$ mice, line TG18, had ~18 copies of Pdia4 transgenes (Figure P6B) and 3 fold higher protein level in beta cells (Figure P6C) than non-transgenic mice (NTG).

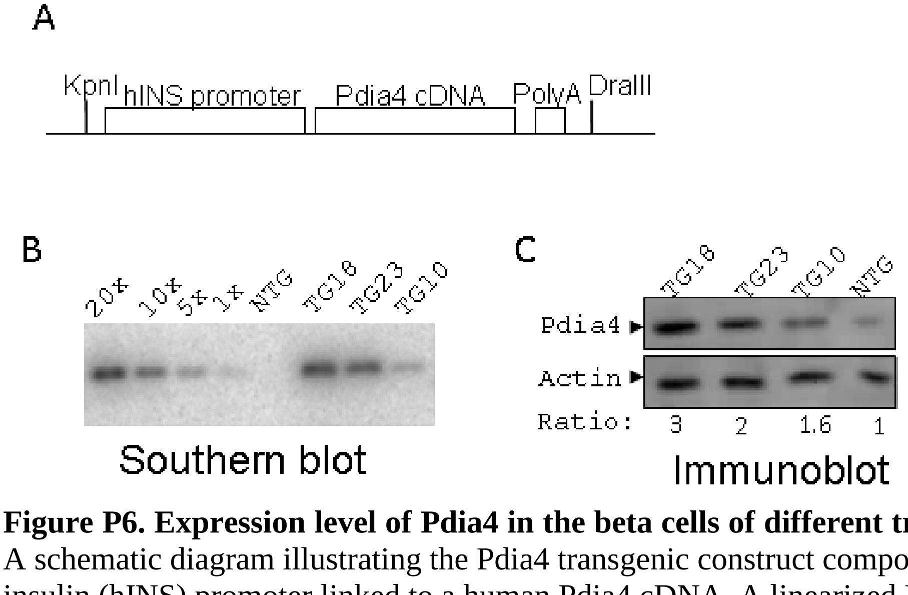

Figure P6. Expression level of Pdia4 in the beta cells of different transgenic lines. (A) A schematic diagram illustrating the Pdia4 transgenic construct composed of the human insulin (hINS) promoter linked to a human Pdia4 cDNA. A linearized KpnI/DraIII fragment from this construct was microinjected into the pronuclei of B6 fertilized eggs to transgenic lines. (B-C) These transgenic lines were characterized using Southern blots.

Plasmid DNA corresponding to 20 copies (20X), 10 copies (10X), 5 copies (5X), and 1 copy (1X) and the genomic DNA from non-transgenic line and transgenic lines 18 (TG18), 23 (TG23) and 10 (TG10) were subjected to DNA gel electrophoresis and hybridized with a Pdia4 probe (B). The Pdia4 level in total lysates of the beta cells of different transgenic mice was characterized using immunoblotting analysis (C). One line TG18 ($Pdia4^{tg/tg}$) was used throughout the study in the revised manuscript.

10. The GHTT studies need either more detailed description or should be removed. I understand that the authors are trying to show therapeutic relevance, but in reality these studies are only presented in a cursory fashion and specificity and mechanism are lacking. Notably, it is unclear how the activity of Pdia4 was specifically tested-it is unclear what the insulin turbidity assay is and how specific that is for Pdia4. Did the compound interfere with interaction with Ndufs3 and p22phox? As is, the description is unclear. Also, IC50 should be expressed in nM or µM and the assay should be described in more detail. Levels of the drug should be measured in circulation (PK parameters). How was the compound synthesized? Details are missing in the methods section.

Response: We have performed a complete pharmacological study of GHTT in *$Lepr^{db/db}$* mice (Figures 8 and S8). We have added the extra data on the impact of GHTT, the Pdia4 inhibitor, on cellular ROS (Figure 8C), interaction between Pdia4 and Ndufs3 and $p22^{phox}$ (Figure 8D), insulin/ROS content in islets (Figure 8E), GTT (Figure S8A), HbA1c (Figure S8B), HOMA indices (Figure S8D) and serum ROS (Figure S8E). Mechanistically speaking, GHTT was shown to disrupt the interaction of Pdia4 and Ndufs3 and $p22^{phox}$ (Figure 8D) and, in turn, reduced ROS content in Min6 cells (Figure 8C). The overall data on the mode of action of Pdia4, using genetic and chemical approaches, concluded that Pdia4 acted through the interaction of Ndufs3 and $p22^{phox}$, key regulators of the electron transport complex 1 and Nox, respectively, leading to increased ROS and, consequently, cell death in beta cells.

There no specific substrate for each Pdi member. Thus, insulin turbidity assay was commonly used to measure Pdi activity though its sensitivity was not great. In our study, we have provided the specificity of GHTT for Pdia4 in comparison with other Pdi members (Pia1, Pdia3 and Pdia6) based on the IC_{50} values. Pdia4 had a two times lower IC_{50} value for GHTT than other Pdi proteins (Table P1) in the responses to the reviewer #2. The data implied that GHTT had a reasonable specificity to Pdia4.

The unit of the IC_{50} values is shown in µM (Figure 8B) and the details about the Pdia4 assay is indicated in the legend of Figure 8B. GHTT was purified from an anti-diabetic plant, *Bidens pilsoa*, as described in the Methods section of the revised manuscript (page 16).

The PK data of GHTT in the plasma of mice following an intravenous injection and oral administration is shown in Figure P7. The data are for the reviewer's reference.

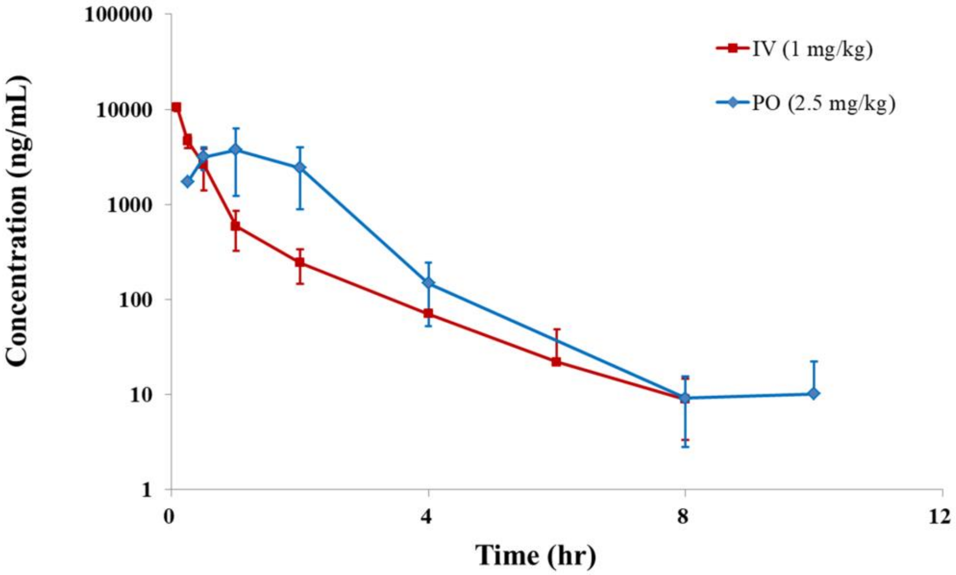

Figure P7. Pharmacokinetic profile of GHTT in the plasma of mice following an intravenous injection and oral administration. *Lepr*$^{db/db}$ mice were received an intravenous injection of GHTT (IV, 1 mg/kg) or an oral dose of GHTT (PO, 2.5 mg/kg). The mice were bled and their plasma samples were subjected to liquid chromatography hyphenated with tandem mass spectroscopy (LC-MS/MS). The mean concentration of GHTT in the plasma of the mice is indicated.

11. As stated above, the significance of this work to human disease is not well addressed. There are limited data with human tissues, and models utilized in this study (db/db) have limited relevance to human diabetes. This, moreover, is not at all discussed in the Discussion (which as noted above needs a re-write).
Response: We have re-written the Discussion section in the revised manuscript and discussed the significance of this work (page 12, first paragraph of the Discussion). We have also discussed the relationship between our mouse models (*Lepr*$^{db/db}$ and B6 mice) used in this study and human diabetes. Since no perfect mouse models of diabetes exist, the *Lepr*$^{db/db}$ mouse model of leptin deficiency is currently the most widely used mouse model of type 2 diabetes mellitus (please refer to Animal Models for the Study of Human Disease (Second Edition, 2017) edited by P. Michael Conn); https://doi.org/10.1155/2017/8503754). Here, we used two mouse models of diabetes, *Lepr*$^{db/db}$ and obesity-induced B6 mice, to complement each other. Most importantly, the overall data led to the same conclusions.

Minor:
12. On page 9, the authors state that "Amazingly, proteomic analysis.....". It is unclear why this shoud be amazing.
Response: We have deleted it (page 9).

13. Supplemental figure quality is poor
Response: We have increased the number of pixels in all the Supplemental Figures to improve their quality in the revised Supplementary Information.

8th Mar 2021

Dear Dr. Yang,

Thank you for the submission of your revised manuscript to EMBO Molecular Medicine. We have now received the enclosed report from the three referees who were asked to re-assess it.

You will see from the comments below that the referees think that while the majority of the concerns have been addressed, several issues remain. During our cross-commenting process (in which the referees are given a chance to make additional comments, including on each other's reports), Referee #3 added, "Referee 1's comments regarding backcrossing and further experimentation are fair. It is possible that backcrossing occurred via a speed congenic approach using microsatellite markers, in which case it is possible that they did the backcross and a 90-week study (though again, they would have to breed up sufficient animals, so it would still be tight). However, this would have to be clearly stated by the authors, and I do think they should show genetic evidence that the mice are congenic."

In light of the referees' comments and the points raised in the reviews below, we think it is essential to address the following issues:
1. Referee #1's concern regarding the backcrossing experiments must be carefully addressed. Genetic evidence and additional experimental details need to be clearly stated in the manuscript text.
2. Please revise the Discussion section by focusing more on the role of Pdia4 according to Referee #3's recommendation. Attention should be paid to avoiding restating the results.

All other concerns raised by Referees #1 and #3 need to be addressed as well. As acceptance or rejection of the manuscript will depend on another round of review, your responses should be as complete as possible.

Please read below for important editorial formatting and consult our author's guidelines for proper formatting your revised article for EMBO Molecular Medicine.

I look forward to receiving your revised manuscript.

Sincerely,
Jingyi

Jingyi Hou
Editor
EMBO Molecular Medicine

*** Instructions to submit your revised manuscript ***

** PLEASE NOTE ** As part of the EMBO Publications transparent editorial process initiative (see our Editorial at https://www.embopress.org/doi/pdf/10.1002/emmm.201000094), EMBO Molecular Medicine will publish online a Review Process File to accompany accepted manuscripts.

In the event of acceptance, this file will be published in conjunction with your paper and will include the anonymous referee reports, your point-by-point response and all pertinent correspondence relating to the manuscript. If you do NOT want this file to be published, please inform the editorial office at contact@embomolmed.org.

To submit your manuscript, please follow this link:

Link not available

When submitting your revised manuscript, please include:

1) a .docx formatted version of the manuscript text (including Figure legends and tables). Please make sure that the changes are highlighted to be clearly visible to referees and editors alike.

2) separate figure files*

3) supplemental information as Expanded View and/or Appendix. Please carefully check the authors guidelines for formatting Expanded view and Appendix figures and tables at https://www.embopress.org/page/journal/17574684/authorguide#expandedview

4) a letter INCLUDING the reviewers' reports and your detailed responses to their comments (as Word file)

Also, and to save some time should your paper be accepted, please read below for additional information regarding some features of our research articles:

5) The paper explained: EMBO Molecular Medicine articles are accompanied by a summary of the articles to emphasize the major findings in the paper and their medical implications for the non-specialist reader. Please provide a draft summary of your article highlighting
- the medical issue you are addressing,
- the results obtained and
- their clinical impact.

This may be edited to ensure that readers understand the significance and context of the research. Please refer to any of our published articles for an example.

6) For more information: There is space at the end of each article to list relevant web links for further consultation by our readers. Could you identify some relevant ones and provide such information as well? Some examples are patient associations, relevant databases,

OMIM/proteins/genes links, author's websites, etc...

7) Author contributions: the contribution of every author must be detailed in a separate section (before the acknowledgments).

8) EMBO Molecular Medicine now requires a complete author checklist (https://www.embopress.org/page/journal/17574684/authorguide) to be submitted with all revised manuscripts. Please use the checklist as a guideline for the sort of information we need WITHIN the manuscript as well as in the checklist. This is particularly important for animal reporting, antibody dilutions (missing) and exact p-values and n that should be indicated instead of a range.

9) Every published paper now includes a 'Synopsis' to further enhance discoverability. Synopses are displayed on the journal webpage and are freely accessible to all readers. They include a short stand first (maximum of 300 characters, including space) as well as 2-5 one sentence bullet points that summarise the paper. Please write the bullet points to summarise the key NEW findings. They should be designed to be complementary to the abstract - i.e. not repeat the same text. We encourage inclusion of key acronyms and quantitative information (maximum of 30 words / bullet point). Please use the passive voice. Please attach these in a separate file or send them by email, we will incorporate them accordingly.

You are also welcome to suggest a striking image or visual abstract to illustrate your article. If you do please provide a jpeg file 550 px-wide x 400-px high.

10) A Conflict of Interest statement should be provided in the main text

11) Please note that we now mandate that all corresponding authors list an ORCID digital identifier. This takes <90 seconds to complete. We encourage all authors to supply an ORCID identifier, which will be linked to their name for unambiguous name identification.

Currently, our records indicate that the ORCID for your account is 0000-0001-6410-2581.

Please click the link below to modify this ORCID:
Link Not Available

12) The system will prompt you to fill in your funding and payment information. This will allow Wiley to send you a quote for the article processing charge (APC) in case of acceptance. This quote takes into account any reduction or fee waivers that you may be eligible for. Authors do not need to pay any fees before their manuscript is accepted and transferred to our publisher.

*Additional important information regarding Figures

Each figure should be given in a separate file and should have the following resolution:
Graphs 800-1,200 DPI
Photos 400-800 DPI
Colour (only CMYK) 300-400 DPI"

Figures are not edited by the production team. All lettering should be the same size and style; figure

panels should be indicated by capital letters (A, B, C etc). Gridlines are not allowed except for log plots. Figures should be numbered in the order of their appearance in the text with Arabic numerals. Each Figure must have a separate legend and a caption is needed for each panel.

*Additional important information regarding figures and illustrations can be found at https://bit.ly/EMBOPressFigurePreparationGuideline

***** Reviewer's comments *****

Referee #1 (Comments on Novelty/Model System for Author):

methodology in the revised manuscript raises the novel concern in this reviewer. Also, the description of the draft (see below) doubts the knowledge of the authors.

Referee #1 (Remarks for Author):

Authors respond to this reviewer's previous concerns. However, some critical issues need to be confirmed.

1. This reviewer raised concerns about the background strain issue in the previous version of the manuscript. It was about a year ago. Authors responded in the letter and also in supplementary method, authors performed backcrossing the strain of mice. However, in this reviewers' knowledge, backcrossing into other strains required for at least about 10 crossings and taking about one year. However, in the manuscript (especially in Fig S3), the authors analyzed 90 weeks of age mice. If this is the product of backcrossing after revision, it is impossible. This reviewer would like to see the proof of backcrossing in each staging (each genomic confirmation). Only SNP analysis cannot be enough in this stage.
2. Islet area of Leprdb/db (18 weeks) in figure 2 B and figure 3B should be similar but indeed opposite pattern. How can it be?
3. Transgenic mice that authors made were based on random integration of injected linearized DNA into the genome. The tg/tg description meant some knock-in of the gene? Also, the description here +/+ in control mice was wrong, at least from the method information.
4. in page 6, in final paragraph describing beta-cell function, the authors stated that Pdia-/- mice displayed better beta-cell function than in WT BKS, but it is not true (Fig S2).
5. Diabetes cannot be described as "reduction or induction" in the animal model. The induction or reduction in number could be public health description.

Referee #3 (Remarks for Author):

The manuscript by Kuo et al. is a resubmission following substantial revision. The authors make the case that Pdia4 promotes the development of ROS in β cells through interaction with Ndufs3 and p22phox and prevents β cell death and dedifferentiation in models of type 2 diabetes in mice. In general, the revisions are substantial with copious data in the main text and supplemental figures that largely make the case the authors are endorsing. Overall, the authors have addressed my concerns, but there are some minor/moderate issues that still need to be addressed:

1. I'm still not convinced that the serum levels of Pdia4 in mice and humans with type 2 diabetes is emanating from the islet or that these represent a potential biomarker in any way. In their response,

the authors are arguing that circulating Pdia4 is arising from islet cells other than β cells or α cells. Considering that islet cell mass makes up a vanishingly small fraction of total body mass, and that β cells and α cells make up ~90% of islet mass, it strains credulity to believe that measurable circulating levels of Pdia4 could be emanating from islets in any form or fashion. Considering that Pdia4 is also expressed in multiple other cell types in the body (Fig. 1), it seems far more likely it is emanating from some combination of these other cells. In which case, the significance and conclusions based on these levels is entirely speculative and should only be presented (if at all) as a curious phenomenon. The speculation about biomarker should be removed entirely, since there is no support for this notion, nor is it clear that such a biomarker would provide any further insight beyond existing biomarkers (e.g. glucose, HbA1c, etc.).

2. Based on Fig. 1 immunoblots, the expression of Pdia4 in total pancreas cannot be accounted for exclusively by expression in the islet, since islet mass is only ~2% of pancreatic mass. Other cells in the pancreas must express Pdia4, and this is not clear from the IF image presented in Fig. 1B, but appears to be evident from the IHC stain (also, why IHC rather than IF here?) in Fig. 1F, where staining is also apparent in acinar cells. The bottom line is that it is not clear that Pdia4 is exclusive to the islet in the pancreas, and the evidence presented by the authors suggests it is in fact expressed in other cell types. This should be acknowledged, and does not detract from the argument of this study.

3. On page 9, the term "amazingly" is retained, and again, it is unclear why this result should be amazing. In general, the authors should avoid opinionated terms in the results section.

4. Finally, the discussion remains problematic. I sense that a big picture has been incorporated, but the discussion is littered with innumerable references to the figures of the paper and, as such, continues to represent mostly a restatement of the results. In general, the perspective the authors are getting across is that ROS is bad for β cells and getting rid of Pdia4 improves ROS. First, the concept of ROS causing β cell dysfunction is simply not a revelation, as I stated in my prior review (as there are numerous examples of where the reduction of ROS improves diabetes in these models). Moreover, whether the advantage of removing Pdia4 results from production of antioxidant enzymes (which is not the case here) or from the generation of such species (i.e. "nipping diabetes in the bud" as the authors rather colloquially state in the discussion) is both highly nuanced and not really tested, since Pdia4 was removed from the inception of β cell development with the Cre model used. In any case, the advance in this study is the role of Pdia4, and the discussion should be focused on the potential mechanisms of Pdia4 and how this study contributes to knowledge of this protein.

Point-to-Point Responses

***** Reviewer's comments *****

Referee #1 (Comments on Novelty/Model System for Author):

methodology in the revised manuscript raises the novel concern in this reviewer. Also, the description of the draft (see below) doubts the knowledge of the authors.

Referee #1 (Remarks for Author):

Authors respond to this reviewer's previous concerns. However, some critical issues need to be confirmed.

1. This reviewer raised concerns about the background strain issue in the previous version of the manuscript. It was about a year ago. Authors responded in the letter and also in supplementary method, authors performed backcrossing the strain of mice. However, in this reviewers' knowledge, backcrossing into other strains required for at least about 10 crossings and taking about one year. However, in the manuscript (especially in Fig S3), the authors analyzed 90 weeks of age mice. If this is the product of backcrossing after revision, it is impossible. This reviewer would like to see the proof of backcrossing in each staging (each genomic confirmation). Only SNP analysis cannot be enough in this stage.
Response: In the Supplementary Information (now renamed Appendix) of the original manuscript, we briefly describe the generation of conventional knockout mice, conditional knockout and transgenic mice of Pdia4 by breeding B6 background to Pdia4 background. We made an honest mistake in omitting to mention an intermediate step in which we crossed B6 mice to BKS mice and, then, *Lepr*$^{db/db}$ mice during this complicated breeding process based on SNP analysis. This mistake was made because we generated and bred the mice through extramural cooperation. In the first revised manuscript, we re-described the detailed procedure of the mouse breeding (But we did not re-cross our mouse lines during the previously (first) revision). As we also wrongly used B6 as the control mice, we corrected all the experiments related to this mistake in the previously (first) revised manuscript.
Regarding the proof of genomic analysis of our three mouse lines, we also wished that we could do so. However, we failed to provide genomic confirmation because the genomic sequence of BKS and *Lepr*$^{db/db}$ BKS mice is lacking and not currently available in public databases. Instead, we performed genetic analysis by comparing 42 microsatellite sequences (Table P1) among B6, *Lepr*$^{db/db}$ BKS (db/db) and our 3 mouse lines (*Pdia4*$^{-/-}$ *Lepr*$^{db/db}$ (KO), *Pdia4*$^{f/f}$*Lepr*$^{db/db}$*Cre*$^{tg/0}$(CKO) and *Pdia4*$^{tg/tg}$ *Lepr*$^{db/db}$ (TG)) based on the literature (Biochem Biphy Acta 1762:440-446). As a result, 33 out of 42 microsatelite markers of the mice were passed for quality control (Table P2). Next, principal component analysis (PCA) data showed that the KO, CKO, and TG mice were the same as db/db mice but far away from B6 mice (Figure

P1). Furthermore, phylogenetic tree data indicated that the KO, CKO, and TG mice were the same as db/db mice but distant from B6 mice based on neighborhood joining and genetic distance modes (Figure P2). Overall genetic analysis of microsatelite markers from 5 strains of the mice suggest that KO, CKO, and TG mice are highly similar to, if not the same as db/db mice in terms of genetic backgrounds. We have described this analysis in the Appendix (lines 34-35, page 3). With these data, we hope that the reviewers can agree that our three mouse lines are presumably congenic strains of db/db mice. This also explains the reason why our TG mice had similar diabetes incidence as db/db mice though the former develop diabetes faster than the latter.
We sincerely appreciate the reviewers' valuable advice to help improve quality of this manuscript.

Table P1. Location and primers of microsatelite markers in *Lepr*$^{db/db}$ mice

S.N.	ID	Chr	Position (Mbp)	PCR-F	PCR-R
1	D1Mit122	1	40.8	TGAAGCAAGAATTCAAGAAGAGG	AGGACATACCTAGTAAGGCTTTGTC
2	D3Mit17	3	144.2	CATGGCTCCATGGTTCTTG	CCACGGAGAACAACTGAAGA
3	D3Mit84	3	144.3	CTGCTCAGAGAACAGCAGATG	TCGAGTCTGTGATTGCTTGG
4	D4Mit246	4	90.7	CAAGAACACAGCACATAAACGT	CTACAAGGCTTGAACAAATTCTTG
5	D4Mit155	4	100.9	GCCTTTCCCTTCAAGTACACC	CAGATTTTAACTGTATGGATGTGTG
6	D4Mit308	4	122.5	TATGGATCCACTCTCCAGAAA	CAAAGTCTCCTCCAAGGCTG
7	D4Mit16	4	126.5	GATCACCCAAGGCTGGC	TCCCCGTGAACTTCCATC
8	D4Mit13	4	141.0	GCTGGTAGCTGGCTTTTCTC	CAGATGTTCCTACTGCTTGG
9	D5Mit431	5	117.3	TCTTCTTGGAGGTCTCCATCA	GGAGCAGAATGGTTTTGGAT
10	D5Mit367	5	117.3	TGGAGAGGAATTCCCTTGC	TGCAGAAGAGAAGCAAGAAGC
11	D7Mit253	7	102.9	TGTGGGTGCAACCAAATG	TTTGGTGATATAGATACTAGGTGTGTG
12	D7Mit238	7	106.4	GCATCTGCTTTTCTGCCTCT	AGGCACCTGACATTGACCTC
13	D8Mit177	8	49.6	CCTCTGCTCTTTAATTTCTTATATTTG	ATAGTCCAAGGTTGTCTTCTGACC
14	D9Mit126	9	23.1	GCTGCCAATCAGGTAAAGGA	GGGGTAGGTATCTGAGCAAGG
15	D9Mit64	9	28.5	TTCACCAAACCTTATCTTACTCCA	TGGAAGAAACAGTGTTGGGT
16	D9Mit259	9	70.1	AGGCTCAAACTAGGACTCAATCC	CCAGAGTCACGGTGAAGTGA
17	D9Mit156	9	55.0	TCGACCCCTGGCTTCTACTA	AGTCTTTTTCTCAAATTCCCACC
18	D9Mit305	9	75.7	AAAAATTTTATTTGTGAGATATGTGTG	CTCCTTGCCCCCAAATTATT
19	D10Mit2	10	21.3	CTGCTCACAACCCATTCCTT	GTTCATTTGAGGCACAAGCA
20	D10Mit15	10	66.8	ATGCGTACAGGCAAAACACC	GCTACATTGGTCTGTGACGC
21	D11Mit71	11	6.8	GCCATACCTGGTAGCGTGTT	AATTTTCAGATGTAGCCATAAGCC
22	D11Mit130	11	44.5	AGCATAAACAGATGTACATGCATATG	CAGGCCCAGAACTCATCTCT
23	D11Mit139	11	48.8	TTTCCCCTTCAATCCACAAG	TTCTTATGAGTGCACACGTGC
24	D11Mit153	11	49.5	ACATAACCCAGCCCTCCC	TCTGTGTTTTTCTCCAAAGGTG
25	D11Mit24	11	53.1	AGGTACATGGCACCACACCT	CAGGTTCCTGGGGTTTGTTA
26	D11Mit111	11	53.3	GAAGGTAAGTGGACCTGAGGG	GCATTTCTCATTCAGATTCGTG
27	D11Mit241	11	54.2	GCCACAAATGTTTAAAGTATAGGAA	CCCAGGGAATGAATGAATACA
28	D11Mit272	11	55.5	CCCTACCATGCACTGCATC	CTTCTTATTATCCAGGAACAGAATCA
29	D11Mit118	11	80.2	CATCTTGGGACTTGGGATGT	GTTGAAAGTTTGCATTTGTCTCC
30	D13Mit139	13	50.8	AGAATAAGTCAAGGCTATGATGTGG	TTGTTTGTTTGTTTGAAGTAGAACG
31	D13Mit146	13	96.8	AAGTTACATTTGTTTTTATTTTGTGTG	GAGCAAAACCTACCAGAAGTGG
32	D13Mit77	13	113.9	TCTTTGAAGTCCCTTTCAAAGC	ATAGCACTGCACTCATGCTCA
33	D15Mit134	15	83.4	GCCAAGTTTGGGGTTTTTGT	AGATAGCAATCTGTCTCCCTGC
34	D15Mit33	15	84.1	CAGGTTGTTAAAGGTGAGACTGG	CTGCATGTATGTACATGGTTGC
35	D15Mit37	15	87.0	TCTGACCTTCACATACACCCC	TGCAACGCAAAACTCAGAAT
36	D15Mit243	15	93.4	GCAGGATATACTTATGTGTTTAGGAGT	CAAATCCATGGAGTGTTTTTG
37	D15Mit76	15	95.4	ACAACACTGCCTTCTTTGCC	GACAGGCATTTTTGGACACA
38	D17Mit221	17	88.7	AACCAGATCATTAACAGTAATAAAGCA	TTGTGGCAAAAACAACCAAA
39	D18Mit110	18	12.1	GGGAGCCGAATTAACACAAA	AACCAAAAACCTTCAAGATAAACA
40	D18Mit20	18	15.1	TGAAAAGACCCTTAGGTTGATACA	CCCTTGATGTCTTTTGTCTTCC
41	D19Mit42	19	9.2	CCAAGGAAACTAGGGTATTTACAC	GCCTAAACCATTCTGTTGTAAGG
42	DXMit211	X	80.7	ATTTAGTGAGCCTAAGATCATGCA	TTGCTCACACACACACAAATATG

Table P2. Amplicon of microsatelite markers in *Lepr*$^{db/db}$ mice

ID	B6	db/db	CKO	KO	TG	Note
D1Mit122	150	150	150	150	150	
D3Mit17	208	202	202	200	200	Low confidence
D3Mit84	164	148	148	148	148	
D4Mit246	96	98	98	96	96	Low confidence
D4Mit155	205	205	205	205	205	
D4Mit308	88	88	88	88	88	
D4Mit16	230	230	230	230	230	
D4Mit13	92	92	92	92	92	
D5Mit431	116	142	142	126	142	Low confidence
D5Mit367	106	106	106	106	106	
D7Mit253	128	126	120	124	120	Low confidence
D7Mit238	156	156	156	141	156	Low confidence
D8Mit177	131	131	131	131	131	
D9Mit126	127	127	127	125	127	Low confidence
D9Mit64	190	186	186	186	186	
D9Mit259	112	120	120	120	120	
D9Mit156	135	135	135	135	135	
D9Mit305	70	70	70	70	70	
D10Mit2	124	85	85	85	85	
D10Mit15	180	180	180	180	180	
D11Mit71	214	211	211	211	211	
D11Mit130	201	197	197	197	197	
D11Mit139	116	116	116	116	116	
D11Mit153	179	179	179	179	179	
D11Mit24	240	238	238	238	238	
D11Mit111	132	138	138	138	140	Low confidence
D11Mit241	126	126	126	126	126	
D11Mit272	122	158	146	152	158	Low confidence
D11Mit118	168	162	162	162	162	
D13Mit139	139	145	145	145	145	
D13Mit146	110	112	112	112	112	
D13Mit77	280	288	288	288	288	
D15Mit134	148	146	146	146	146	
D15Mit33	152	170	170	170	170	
D15Mit37	148	148	148	148	148	
D15Mit243	127	127	127	127	127	
D15Mit76	250	250	250	250	250	
D17Mit221	139	135	135	135	135	
D18Mit110	149	139	139	139	139	
D18Mit20	184	186	186	186	186	
D19Mit42	178	178	178	178	178	
DXMit211	116	144	116	128	116	Low confidence

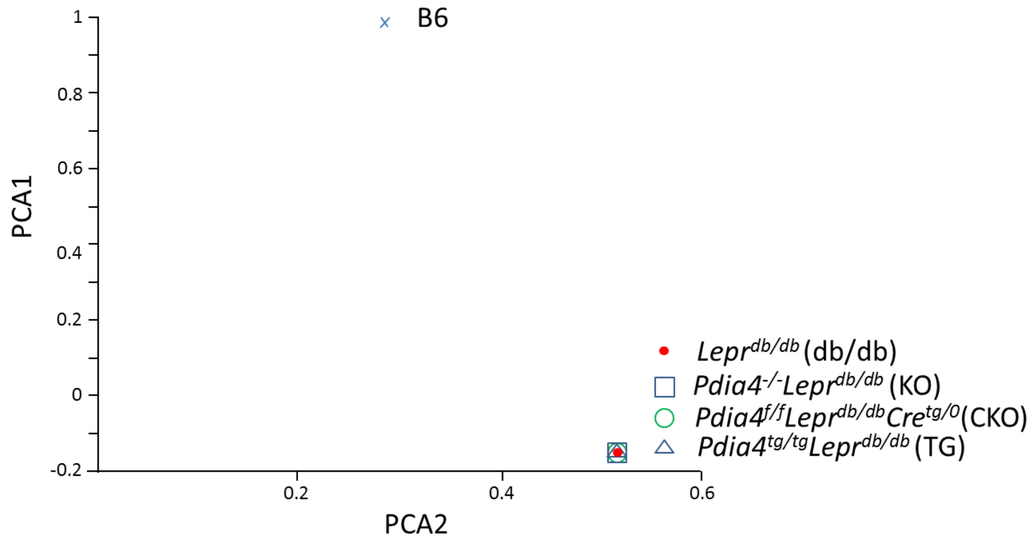

Figure P1. Principal Component Analysis (PCA) of the microsatelite makers in 5 strains of mice. Genomic DNA from B6, *Lepr*$^{db/db}$ (db/db) and 3 mouse lines (*Pdia4*$^{-/-}$ *Lepr*$^{db/db}$ (KO), *Pdia4*$^{f/f}$*Lepr*$^{db/db}$*Cre*$^{tg/0}$ (CKO) and Pdia4$^{tg/tg}$ *Lepr*$^{db/db}$ (TG)) were extracted. The DNA was used as template together with 42 pairs of primers to perform PCR. Thirty-three out of 42 microsatelite markers from 5 strains of mice were selected for PCA analysis based on their DNA sequences. We abandoned these 9 markers, labelled “low confidence”, in PCA analysis because they had a flaw in their PCR amplification or next-generation sequencing.

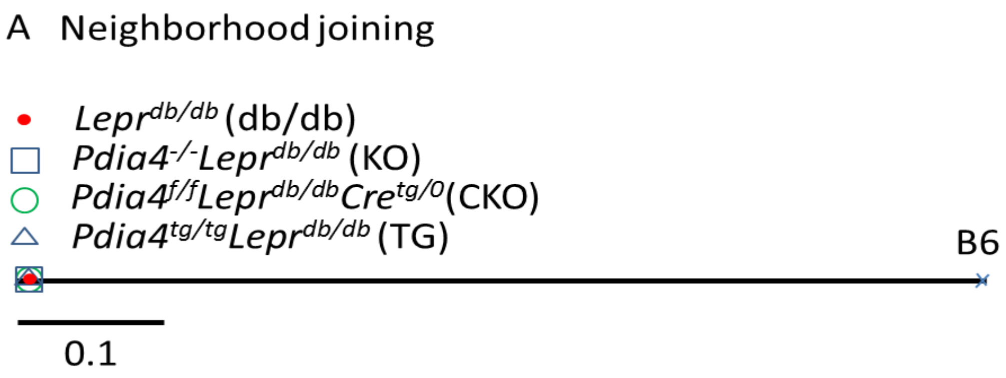

B Matrix of genetic distance

	B6	db/db	KO	CKO	TG
B6	0	0.6633	0.6633	0.6633	0.6633
db/db	0.6633	0	0	0	0
KO	0.6633	0	0	0	0
CKO	0.6633	0	0	0	0
TG	0.6633	0	0	0	0

Figure P2. Phylogenetic tree analysis of the microsatelite makers in 5 strains of mice. Thirty-three microsatelite markers of the mice (Figure P1) were selected for phylogenetic tree analysis based on their DNA sequences. Their neighborhood joining mode (A) and matrix of genetic distance (B) are indicated.

2. Islet area of Leprdb/db (18 weeks) in figure 2 B and figure 3B should be similar but indeed opposite pattern. How can it be?

Response: The islet area of *Lepr*$^{db/db}$ mice in Figure 2 B (18 weeks) and Figure 3B (14 weeks) was in fact distinct because their islet areas declined with the severity of diabetes. By 14 weeks of age, the average islet area of *Lepr*$^{db/db}$ mice was 49,403 µm^2 (Figure 3 B). However, that of Pdia4$^{tg/tg}$ *Lepr*$^{db/db}$ mice was reduced to 22,560 µm^2 (Figure 3 B). The data showed that Pdia4 transgenic mice had accelerated progression and deterioration in diabetes.

In sharp contrast, by 18 weeks of age, the average islet area of *Lepr*$^{db/db}$ mice was quickly reduced to 13,380 µm^2 (Figure 2B). However, the average islet area of *Pdia4*$^{-/-}$*Lepr*$^{db/db}$ mice was maintained at 37,648 µm^2 (Figure 2B). The data showed that Pdia4 knockout mice ameliorated diabetes.

3. Transgenic mice that authors made were based on random integration of injected linearized DNA into the genome. The tg/tg description meant some knock-in of the gene? Also, the description here +/+ in control mice was wrong, at least from the method information.

Response: The reviewer was right that our transgenic mice were made based on random insertion of injected linearized DNA into the genome of the mice. However, we did not confirm an insertion site of the Pdia4 transgene in the genome of transgenic mice. Based on the Jackson Lab guidelines (https://www.jax.org/news-and-insights/jax-blog/2011/may/designating-genotypes-what-does-plus-really-mean20150422t150455), a precise way of indicating wild-type mice (*Pdia4*$^{+/+}$;TgPdia4$^{+/+}$), Pdia4 knockout (*Pdia4*$^{-/-}$;TgPdia4$^{+/+}$), and Pdia4 transgenic mice (*Pdia4*$^{+/+}$;TgPdia4$^{tg/tg}$), can be used but is a little complicated for the readers. Instead, to prevent the ambiguity of control mice versus transgenic mice, we used wild-type control mice (WT), Pdia4 knockout (KO) and Pdia4 transgenic mice (TG) in all the figures of the current revised manuscript and Appendix.

4. in page 6, in final paragraph describing beta-cell function, the authors stated that Pdia-/- mice displayed better beta-cell function than in WT BKS, but it is not true (Fig S2).

Response: *Pdia4*$^{-/-}$ beta cells did have better insulin secretion, a signature of beta cells, than WT beta cells regardless of mouse background as shown in Figure 2F. If you just check the HOMA-beta index in Figure S2D and serum insulin in Figure 2E, you fail to see this nuance in beta cell function between *Pdia4*$^{-/-}$ and WT BKS mice. However, a combination of the data in Figures 2E, 2F and S2 come to this conclusion.

5. Diabetes cannot be described as "reduction or induction" in the animal model. The induction or reduction in number could be public health description.

Response: We thank the reviewer for the comment. We have erased "diabetes" from "reduction of diabetes" (line 30, page 5, the revised manuscript) and "To induce diabetes, ..." (line 41, page 3, Appendix)

Referee #3 (Remarks for Author):

The manuscript by Kuo et al. is a resubmission following substantial revision. The authors make the case that Pdia4 promotes the development of ROS in β cells through interaction with Ndufs3 and p22phox and prevents β cell death and dedifferentiation in models of type 2 diabetes in mice. In general, the revisions are substantial with copious data in the main text and supplemental figures that largely make the case the authors are endorsing. Overall, the authors have addressed my concerns, but there are some minor/moderate issues that still need to be addressed:

1. I'm still not convinced that the serum levels of Pdia4 in mice and humans with type 2 diabetes is emanating from the islet or that these represent a potential biomarker in any way. In their response, the authors are arguing that circulating Pdia4 is arising from islet cells other than β cells or α cells. Considering that islet cell mass makes up a vanishingly small fraction of total body mass, and that β cells and α cells make up ~90% of islet mass, it strains credulity to believe that measurable circulating levels of Pdia4 could be emanating from islets in any form or fashion. Considering that Pdia4 is also expressed in multiple other cell types in the body (Fig. 1), it seems far more likely it is emanating from some combination of these other cells. In which case, the significance and conclusions based on these levels is entirely speculative and should only be presented (if at all) as a curious phenomenon. The speculation about biomarker should be removed entirely, since there is no support for this notion, nor is it clear that such a biomarker would provide any further insight beyond existing biomarkers (e.g. glucose, HbA1c, etc.).
Response: We have deleted the whole part related to the use of Pdia4 as a biomarker from the Results (Line 26-28, page 5) and Discussion (last paragraph, page 12).

2. Based on Fig. 1 immunoblots, the expression of Pdia4 in total pancreas cannot be accounted for exclusively by expression in the islet, since islet mass is only ~2% of pancreatic mass. Other cells in the pancreas must express Pdia4, and this is not clear from the IF image presented in Fig. 1B, but appears to be evident from the IHC stain (also, why IHC rather than IF here?) in Fig. 1F, where staining is also apparent in acinar cells. The bottom line is that it is not clear that Pdia4 is exclusive to the islet in the pancreas, and the evidence presented by the authors suggests it is in fact expressed in other cell types. This should be acknowledged, and does not detract from the argument of this study.
Response: We used confocal microscopy to indicate the colocalization of Pdia4 and insulin in β-cells (Figure 1B). We used different techniques to confirm the protein level of Pdia4 in β-cells. For instance, IHC was used to show the protein level of

Pdia4 in Figure 1F. The discrepancy of the Pdia4 level in β-cells may be due to the difference of detection sensitivity of the two techniques.
We agree with the reviewer that Pdia4 may not only be expressed in β-cells although Pdia4 was not expressed in α-cells of mouse islets (Figure 1B). Therefore, we have modified the paragraph from "Further, Pdia4 was exclusively expressed in β-cells but not α-cells of mouse islets (Figure 1B)." to "Further, Pdia4 was expressed in β-cells but not α-cells of mouse islets (Figure 1B). However, we could not rule out its expression in other pancreatic cell types."

3. On page 9, the term "amazingly" is retained, and again, it is unclear why this result should be amazing. In general, the authors should avoid opinionated terms in the results section.
Response: We have deleted this word (line 3, page 10).

4. Finally, the discussion remains problematic. I sense that a big picture has been incorporated, but the discussion is littered with innumerable references to the figures of the paper and, as such, continues to represent mostly a restatement of the results. In general, the perspective the authors are getting across is that ROS is bad for β cells and getting rid of Pdia4 improves ROS. First, the concept of ROS causing β cell dysfunction is simply not a revelation, as I stated in my prior review (as there are numerous examples of where the reduction of ROS improves diabetes in these models). Moreover, whether the advantage of removing Pdia4 results from production of antioxidant enzymes (which is not the case here) or from the generation of such species (i.e. "nipping diabetes in the bud" as the authors rather colloquially state in the discussion) is both highly nuanced and not really tested, since Pdia4 was removed from the inception of β cell development with the Cre model used. In any case, the advance in this study is the role of Pdia4, and the discussion should be focused on the potential mechanisms of Pdia4 and how this study contributes to knowledge of this protein.
Response: The authors really appreciate the constructive advice given by the reviewer for improving our discussion section. In the re-written Discussion section, in the first paragraph, we have made a summary of the results with a generalized view of the most important findings in this study. In the second paragraph, we have discussed the cellular and molecular mechanism of Pdia4 in β-cell death and dysfunction during diabetes. We have touched on the role of Pdia4 in cell death, as evidenced with specific markers of autophagy and dedifferentiation, but not cell proliferation and differentiation in β-cells. We have also discussed the action of Pdia4 on β-cell function. The molecular basis of Pdia4 in β-cell failure is associated with the Ndufs3 and $p22^{phox}$ pathways in a sequence-specific manner. The Pdia4 inhibitor, GHTT, disrupted thePdia4/Ndufs3 and $p22^{phox}$ pathways and therefore improved β-cell failure. Genetics and pharmaceutical data demonstrate the novel function and molecular basis of the chaperone, Pdia4, in β-cells in terms of oxidative stress, dysfunction and death and extend our understanding of islet biology. We have also discussed some limitations in our genetics approaches. In the third paragraph, we have further discussed the potential of Pdia4 to treat diabetes in

mouse models; we discussed the results from *Lepr*$^{db/db}$ mice and HFD-fed B6 models and the relevance of both mouse models to human diabetes. Pdia4 deficiency and inactivation also improved β-cell failure and diabetes in mouse models. Targeting Pdia4 and its pathways may thus constitute attractive approaches for the treatment of β-cell pathogenesis and diabetes. In the fourth paragraph, we present the "big picture" implied by this work as follows: hyperglycemia/excess nutrients → Pdia4 → Ndufs3 and p22^{phox} → ROS → β cell failure and diabetes. Finally, we have discussed the contribution of this study to knowledge of Pdia4. The advances include characterization of Pdia4 and partners, molecular interplay, non-ER-relevant functions of Pdia4, mechanism by which Pdia4 is up-regulated by glucose, and comparison of Pdia4 with other Pdis. We hope that these modifications will now constitute ample scholarly discussion of this study.

19th May 2021

Thank you for the submission of your revised manuscript to EMBO Molecular Medicine. We have now received the enclosed report from the two referees who were asked to re-assess it. As you will see below, Referee #3 is satisfied with the revisions and is now supportive of publication. Referee #1 still raised several concerns, for which we would ask you to discuss the limitations and future directions in writing.

1. In the main manuscript file, please do the following:
- Reduce keyword number to 5.
- For animal work, this manuscript must include a statement in the Materials and Methods identifying the institutional and/or licensing committee approving the experiments. Gender, age and genetic background must be indicated, along with housing conditions.
- In Materials and Methods, include a statement that informed consent was obtained from all human subjects and that the experiments conformed to the principles set out in the WMA Declaration of Helsinki and the Department of Health and Human Services Belmont Report.

2. Data availability:
- please add a formal "Data Availability" section (placed after Materials & Method).
- For the proteomics data, the accession numbers and database should be listed in a formal "Data Availability" section that follows the model below (see also https://www.embopress.org/page/journal/17574684/authorguide#dataavailability). Please note that the Data Availability Section is restricted to new primary data that are part of this study.
Data availability

The datasets produced in this study are available in the following databases:
- RNA-Seq data: Gene Expression Omnibus GSE46843 (https://www.ncbi.nlm.nih.gov/geo/query/acc.cgi?acc=GSE46843)
- [data type]: [name of the resource] [accession number/identifier/doi] ([URL oridentifiers.org/DATABASE:ACCESSION])

3. Appendix:
-Please add a Table of Content on the 1st page.
-Please move the Methods to main manuscript file and merge with the Materials and Methods section.
-Fig S8 and Fig S9 should be called "Appendix Figure S8 and Appendix Figure S9.

4. For more information: There is space at the end of each article to list relevant web links for further consultation by our readers. Could you identify some relevant ones and provide such information as well? Some examples are patient associations, relevant databases, OMIM/proteins/genes links, author's websites, etc...

5. We would encourage you to include the source data for figure panels that show essential data. Numerical data should be provided as individual .xls or .csv files (including a tab describing the data). For blots or microscopy, uncropped images should be submitted (using a zip archive if multiple images need to be supplied for one panel). Additional information on source data and instruction on

how to label the files are available at
https://www.embopress.org/page/journal/17574684/authorguide#sourcedata

6. Our data editors have seen the manuscript, and they have made some comments and suggestions that need to be addressed (see attached). Please send back a revised version (in track change mode), as we will need to go through the changes.

7. I have slightly modified the synopsis text and The Paper Explained (see attached manuscript file). Please let me know if you are fine with it or if you would like to introduce further modifications. Please note that these would be the final version, and changes during proofing are usually not allowed.

8. As part of the EMBO Publications transparent editorial process initiative (see our Editorial at http://embomolmed.embopress.org/content/2/9/329), EMBO Molecular Medicine will publish online a Review Process File (RPF) to accompany accepted manuscripts.
a. In the event of acceptance, this file will be published in conjunction with your paper and will include the anonymous referee reports, your point-by-point response and all pertinent correspondence relating to the manuscript. Let us know if you do NOT agree with this.

b. Please note that the Authors checklist will be published at the end of the RPF.

I look forward to seeing a revised version of your manuscript as soon as possible.

Sincerely,
Jingyi

Jingyi Hou
Editor
EMBO Molecular Medicine

*** Instructions to submit your revised manuscript ***

*** PLEASE NOTE *** As part of the EMBO Publications transparent editorial process initiative (see our Editorial at https://www.embopress.org/doi/pdf/10.1002/emmm.201000094), EMBO Molecular Medicine will publish online a Review Process File to accompany accepted manuscripts.

In the event of acceptance, this file will be published in conjunction with your paper and will include the anonymous referee reports, your point-by-point response and all pertinent correspondence relating to the manuscript. If you do NOT want this file to be published, please inform the editorial office at contact@embomolmed.org.

To submit your manuscript, please follow this link:

Link not available

When submitting your revised manuscript, please include:

1) a .docx formatted version of the manuscript text (including Figure legends and tables)

2) Separate figure files*

3) supplemental information as Expanded View and/or Appendix. Please carefully check the authors guidelines for formatting Expanded view and Appendix figures and tables at https://www.embopress.org/page/journal/17574684/authorguide#expandedview

4) a letter INCLUDING the reviewer's reports and your detailed responses to their comments (as Word
file).

5) The paper explained: EMBO Molecular Medicine articles are accompanied by a summary of the articles to emphasize the major findings in the paper and their medical implications for the non-specialist reader. Please provide a draft summary of your article highlighting
- the medical issue you are addressing,
- the results obtained and
- their clinical impact.
This may be edited to ensure that readers understand the significance and context of the research. Please refer to any of our published articles for an example.

6) For more information: There is space at the end of each article to list relevant web links for further consultation by our readers. Could you identify some relevant ones and provide such information as well? Some examples are patient associations, relevant databases, OMIM/proteins/genes links, author's websites, etc...

7) Author contributions: the contribution of every author must be detailed in a separate section.

8) EMBO Molecular Medicine now requires a complete author checklist (https://www.embopress.org/page/journal/17574684/authorguide) to be submitted with all revised manuscripts. Please use the checklist as guideline for the sort of information we need WITHIN the manuscript. The checklist should only be filled with page numbers were the information can be found. This is particularly important for animal reporting, antibody dilutions (missing) and exact values and n that should be indicted instead of a range.

9) Every published paper now includes a 'Synopsis' to further enhance discoverability. Synopses are displayed on the journal webpage and are freely accessible to all readers. They include a short stand first (maximum of 300 characters, including space) as well as 2-5 one sentence bullet points that summarise the paper. Please write the bullet points to summarise the key NEW findings. They should be designed to be complementary to the abstract - i.e. not repeat the same text. We encourage inclusion of key acronyms and quantitative information (maximum of 30 words / bullet point). Please use the passive voice. Please attach these in a separate file or send them by email, we will incorporate them accordingly.

You are also welcome to suggest a striking image or visual abstract to illustrate your article. If you do please provide a jpeg file 550 px-wide x 400-px high.

10) A Conflict of Interest statement should be provided in the main text

11) Please note that we now mandate that all corresponding authors list an ORCID digital identifier. This takes <90 seconds to complete. We encourage all authors to supply an ORCID identifier, which will be linked to their name for unambiguous name identification.

Currently, our records indicate that the ORCID for your account is 0000-0001-6410-2581.

Please click the link below to modify this ORCID:
Link Not Available

12) The system will prompt you to fill in your funding and payment information. This will allow Wiley to send you a quote for the article processing charge (APC) in case of acceptance. This quote takes into account any reduction or fee waivers that you may be eligible for. Authors do not need to pay any fees before their manuscript is accepted and transferred to our publisher.

*Additional important information regarding Figures

Each figure should be given in a separate file and should have the following resolution:
Graphs 800-1,200 DPI
Photos 400-800 DPI
Colour (only CMYK) 300-400 DPI"

Figures are not edited by the production team. All lettering should be the same size and style; figure panels should be indicated by capital letters (A, B, C etc). Gridlines are not allowed except for log plots. Figures should be numbered in the order of their appearance in the text with Arabic numerals. Each Figure must have a separate legend and a caption is needed for each panel.

*Additional important information regarding figures and illustrations can be found at https://bit.ly/EMBOPressFigurePreparationGuideline

The system will prompt you to fill in your funding and payment information. This will allow Wiley to send you a quote for the article processing charge (APC) in case of acceptance. This quote takes into account any reduction or fee waivers that you may be eligible for. Authors do not need to pay any fees before their manuscript is accepted and transferred to our publisher.

***** Reviewer's comments *****

Referee #1 (Comments on Novelty/Model System for Author):

Still, molecular mechanisms are not clearly shown, and the relevance of some molecules in the manuscript was not clear yet. Although Pdia4 paly roles in beta-cell, Pdia4 is also high systemically, then whether GHTT effects are solely on beta-cell were not elucidated yet.

Referee #1 (Remarks for Author):

Authors described that the Pdia4, the member of protein disulfide isomerases (Pdis),

overexpression could be involved in the onset of beta-cell failure in type 2 diabetes. In vivo, Pdia4 deficiency protected beta-cell loss in db/db mice, whereas transgenic overexpression of Pdia4 deteriorated the disease. Functionally, Pdia4 is associated with the induction of autophagic molecules (beclin and LC3), mitochondrial deficiency, and induction of oxidative stress via interaction with Ndufs and P22phox. Finally, experimental therapy was conducted with GHTT.

1. Autophagic molecules: the current presentation of data did not show any conclusion, and the role of these molecular alterations was not investigated yet. Authors must show p62 and also lysosomal fusion proteins whether Pdia4 overexpression induced either autophagic flux or premature stop via inhibition of autolysosome vacuole formation. If p62 is accumulated, perhaps, p62 accumulation induced intracellular signaling to induce ROS formation.
2. ROS plays a pathologic role in beta-cell dysfunction. ROS could be either inducing or induced by mitochondria dysfunction. Authors showed that anti-oxidant vitC did not rescue db/db phenotype; however, at the same time, HG + PDia4 OE-induced ROS was abolished entirely by NAC (Fig7). The question is that such effects of Pdia4 and also NAC could link to the preservation of insulin-producing min6 cells and isolated islets from Tg mice? Also, beta-cell morphology? These data must be shown.
3. GHTT intervention could influence systemic metabolism. Authors should treat beta-cell specific Pdia4 KO db/db mice (CKO leprdb/db) to see whether the metabolic effects of GHTT was solely effects on beta-cell or not.
4. Some of the sentences are hard to follow, such as page 11, line4 to line7. Also, what is GK meant? If this is "scramble", say scramble in the figure as well. Also, in the same page second paragraph, line 2, tNdufs3 are introduced without explaining as a "truncated mutant." not only that, the whole manuscript was tough to follow, by not the grammar, but the description issues.
5. Alteration of BW should be shown in all in vivo data.

Referee #3 (Remarks for Author):

No further concerns

***** Reviewer's comments *****

Referee #1 (Comments on Novelty/Model System for Author):

Still, molecular mechanisms are not clearly shown, and the relevance of some molecules in the manuscript was not clear yet. Although Pdia4 paly roles in beta-cell, Pdia4 is also high systemically, then whether GHTT effects are solely on beta-cell were not elucidated yet.

Referee #1 (Remarks for Author):

Authors described that the Pdia4, the member of protein disulfide isomerases (Pdis), overexpression could be involved in the onset of beta-cell failure in type 2 diabetes. In vivo, Pdia4 deficiency protected beta-cell loss in db/db mice, whereas transgenic overexpression of Pdia4 deteriorated the disease. Functionally, Pdia4 is associated with the induction of autophagic molecules (beclin and LC3), mitochondrial deficiency, and induction of oxidative stress via interaction with Ndufs and P22phox. Finally, experimental therapy was conducted with GHTT.

1. Autophagic molecules: the current presentation of data did not show any conclusion, and the role of these molecular alterations was not investigated yet. Authors must show p62 and also lysosomal fusion proteins whether Pdia4 overexpression induced either autophagic flux or premature stop via inhibition of autolysosome vacuole formation. If p62 is accumulated, perhaps, p62 accumulation induced intracellular signaling to induce ROS formation.
Response: The immunoblotting analysis of p62 has been conducted and added to Fig. 4B. The results have been described in the Results (lines 33-37, page 8)

2. ROS plays a pathologic role in beta-cell dysfunction. ROS could be either inducing or induced by mitochondria dysfunction. Authors showed that anti-oxidant vitC did not rescue db/db phenotype; however, at the same time, HG + PDia4 OE-induced ROS was abolished entirely by NAC (Fig7). The question is that such effects of Pdia4 and also

NAC could link to the preservation of insulin-producing min6 cells and isolated islets from Tg mice? Also, beta-cell morphology? These data must be shown.

Response: Vitamin C and N-acetyl cysteine (NAC) were used as anti-oxidant controls in Appendix Fig. S2E and Fig. 7A. However, Pdia4 depletion seemed to have advantages over the anti-oxidants as one cited reference (Chang YC, et al. (2010) Am J Transl Res 2: 316-331) because Pdia4 deficiency nipped diabetes in the bud by lowering ROS production, a different mechanism from the elimination of ROS by anti-oxidants, in which the cells continued to produce ROS through the ROS-generating machinery.

Our data in Min6 cells showed that three lines of Min6 cells, GK, KD and OVE cells, had a basal level of cell death in medium containing low glucose (LG) in the absence and presence of NAC (LG, Fig. P1). In contrast, Min6 OVE cells had a higher level of cell death in medium containing high glucose (HG) than Min6 GK cells and Min6 KD cells (HG, Fig. P1). Similarly, Min6 OVE cells had a higher level of cell death in medium containing high glucose plus NAC (HG+NAC) than Min6 GK cells and Min6 KD cells (HG+NAC, Fig. P1). Consistently, our data in mouse islets showed that *Pdia4*$^{tg/tg}$ islets, WT islets and *Pdia4*$^{-/-}$ islets had a basal level of cell death in medium containing low glucose (LG) in the absence and presence of NAC (LG, Fig. P2) as evidenced by propidium iodide (PI) signal. In contrast, *Pdia4*$^{tg/tg}$ islets had a higher level of cell death in medium containing high glucose (HG) than WT islets and *Pdia4*$^{-/-}$ islets (HG, Fig. P2). Similarly, *Pdia4*$^{tg/tg}$ islets had a higher level of cell death in medium containing high glucose plus NAC (HG+NAC) than WT islets and *Pdia4*$^{-/-}$ islets (HG+NAC, Fig. P2).

Our data demonstrated that Pdia4 depletion reduced β-cell death. Although NAC could reduce ROS in Min6 cells and islets, NAC failed to effectively reduce β-cell death.

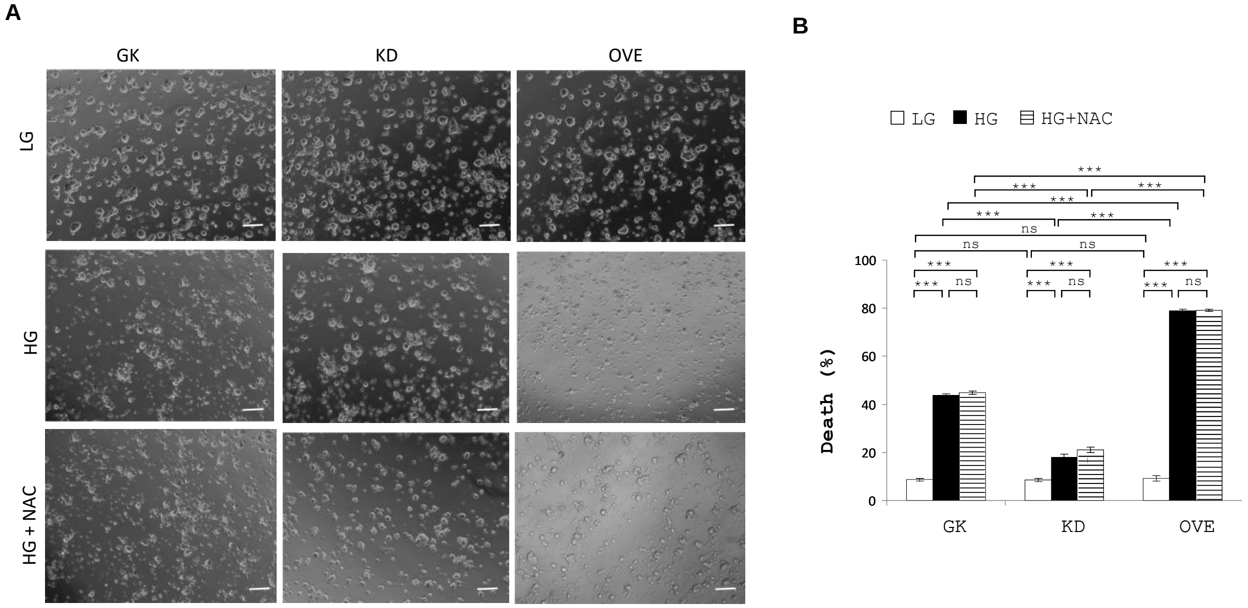

Fig. P1. Comparison of cell death in three cell lines of Min6 cells. (A-B) Min6 GK cells, a scramble control, Min6 KD cells with Pdia4 knockdown, and Min6 OVE cells with Pdia4

overexpression, were grown for 24 h. The cells were then incubated with RPMI medium containing 0.5 mM (LG) and 25 mM (HG) glucose in the absence or presence of 1 mM NAC for overnight. The cells were photographed (A). Scale bar = 100 μm. After trypsin treatment, the cells were stained with trypan blue and counted for live and dead cells (B).

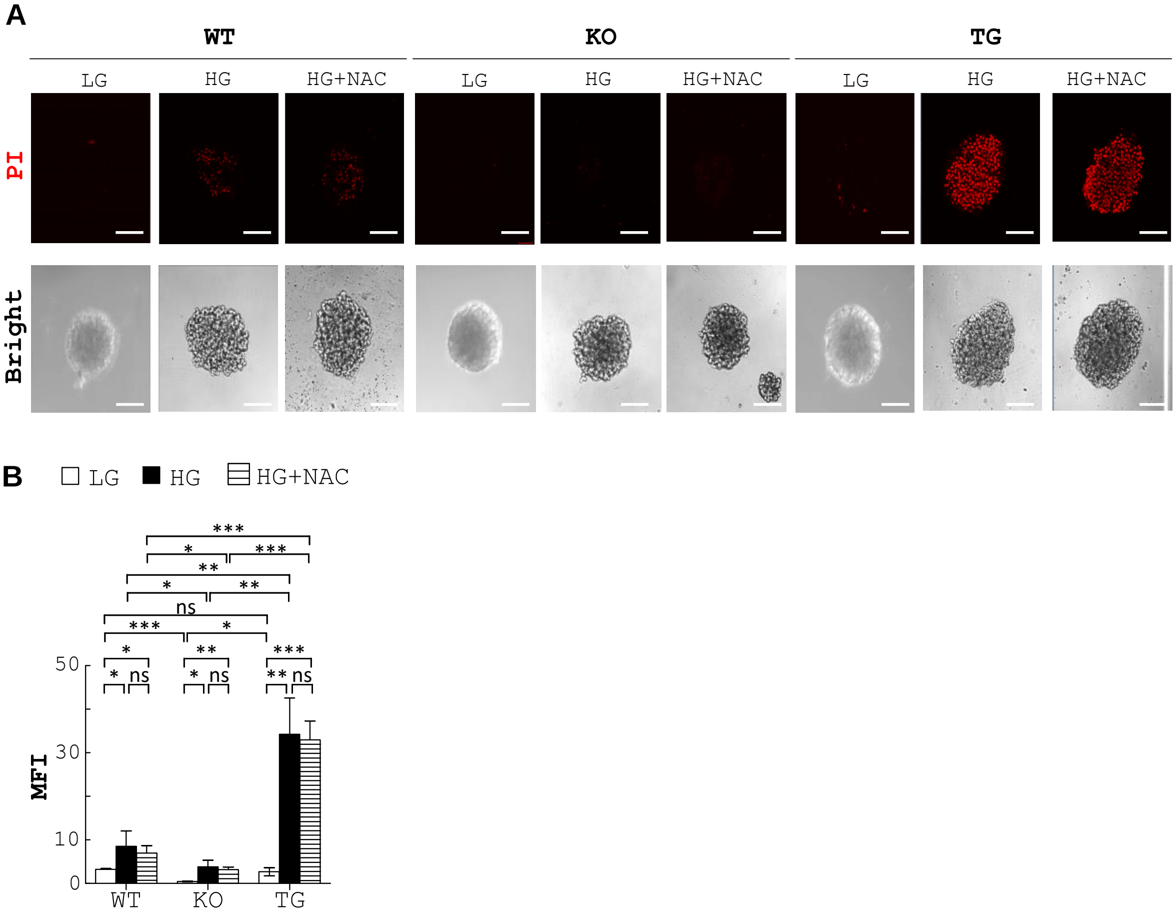

Fig. P2. Comparison of cell death in three cell lines of mouse pancreatic islets. (A) The islets of wild-type (WT), *Pdia4*$^{-/-}$ (KO) and Pdia4$^{tg/tg}$ (TG) BKS mice were isolated and grown in complete DMEM medium containing 3.3 mM (LG) and 30 mM glucose (HG) in the absence or presence of 1 mM NAC for 12 h. The islets were stained with propidium iodide (PI) and photographed. Scale bar = 100 μm. (B) The PI signal, expressed in mean fluorescent intensity (MFI), in the mouse islets of each group was quantified and re-plotted into histograms (B).

3. GHTT intervention could influence systemic metabolism. Authors should treat beta-cell specific Pdia4 KO db/db mice (CKO leprdb/db) to see whether the metabolic effects of GHTT was solely effects on beta-cell or not.
Response: We treated islet-specific Pdia4 KO *lepr*$^{db/db}$ (CKO *lepr*$^{db/db}$) with PBS vehicle and GHTT for 4 weeks. The data showed that GHTT did not significantly affect the diabetes in CKO *lepr*$^{db/db}$ mice as evidenced by fasting blood glucose and postprandial blood glucose (Fig. P3). The data suggest that GHTT controls diabetes mainly via targeting Pdia4 in the islets.

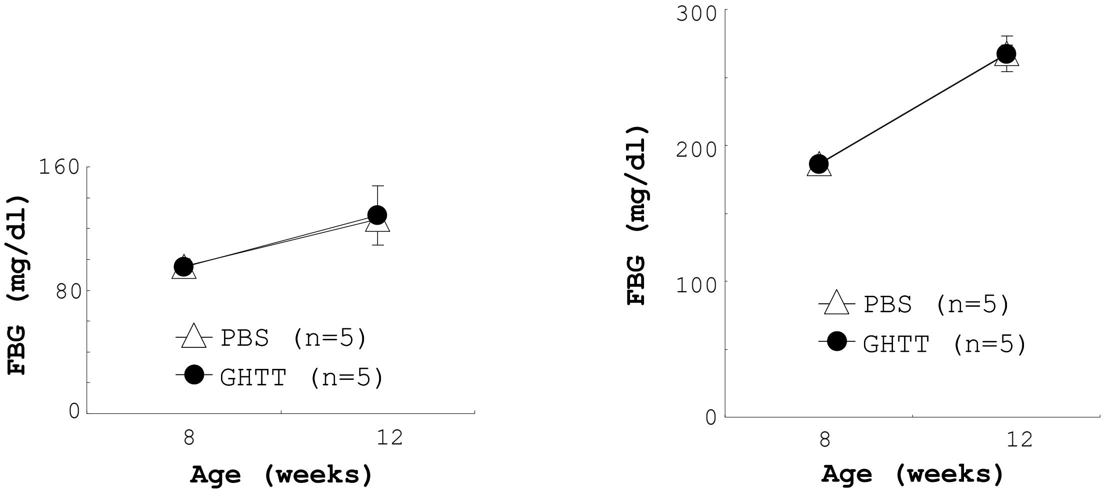

Fig. P3. GHTT cannot lower blood glucose in islet-specific Pdia4 KO *lepr*$^{db/db}$ (CKO *lepr*$^{db/db}$) mice. The islet-specific Pdia4 KO *lepr*$^{db/db}$ (CKO *lepr*$^{db/db}$) mice, aged 8 weeks, were randomly divided into 2 groups. Both groups of mice were treated with PBS vehicle and GHTT (25 mg/kg) for 4 weeks. FBG and PBG of islet-specific knockout (CKO *Lepr*$^{db/db}$) mice were determined. The number (n) of mice is indicated in parentheses.

4. Some of the sentences are hard to follow, such as page 11, line4 to line7. Also, what is GK meant? If this is "scramble", say scramble in the figure as well. Also, in the same page second paragraph, line 2, tNdufs3 are introduced without explaining as a "truncated mutant." not only that, the whole manuscript was tough to follow, by not the grammar, but the description issues.
Response: We thank the Reviewer for the comment. We have rephrased the sentences (lines 4-7, page 11). We have indicated that GK is a scramble control in the text and figure legend (Fig. 7). tNdufs3 and tp22^{phox} were a mutant of Ndufs3, containing N-terminal 1 to 132 aa, and a mutant of p22^{phox}, containing N-terminal 1 to 99 aa, respectively. Both have been defined in the revised manuscript (lines 18-21, page 11). In addition, we have rechecked the whole manuscript to look for descriptions that are tough to follow, and made any changes we can to try and make the manuscript as clear and easy to follow as possible.

5. Alteration of BW should be shown in all in vivo data.
Response: Body weight in *Pdia4*$^{-/-}$*Lepr*$^{db/db}$ mice (right, Appendix Fig. S2C), islet-specific Pdia4 knockout mice (*Pdia*$^{f/f}$*Lepr*$^{db/db}$*Cre*$^{tg/0}$) **(Appendix Fig. S4G), HFD-fed wild-type,** Pdia4$^{tg/tg}$, and ***Pdia4***$^{-/-}$ **B6 mice (right, Appendix Fig. S5E),** Pdia4$^{tg/tg}$ ***Lepr***$^{db/db}$ **mice (Appendix Fig. S6C), and GHTT-treated mice (Appendix Fig. S8F) are shown. Accordingly, we have also modified the legends of the Appendix Figures and the description in the revised manuscript.**

Referee #3 (Remarks for Author):

No further concerns

3rd Revision - Editorial Decision 18th Aug 2021

18th Aug 2021

I am pleased to inform you that your manuscript is accepted for publication and is now being sent to our publisher to be included in the next available issue of EMBO Molecular Medicine.

We would like to remind you that as part of the EMBO Publications transparent editorial process initiative, EMBO Molecular Medicine will publish a Review Process File online to accompany accepted manuscripts. If you do NOT want the file to be published or would like to exclude figures, please immediately inform the editorial office via e-mail.

Please read below for additional IMPORTANT information regarding your article, its publication and the production process.

Congratulations on your interesting work,

Kind regards,
Jingyi

Jingyi Hou
Editor
EMBO Molecular Medicine

Follow us on Twitter @EmboMolMed
Sign up for eTOCs at embopress.org/alertsfeeds

***** Reviewer's comments *****

EMBO PRESS

YOU MUST COMPLETE ALL CELLS WITH A PINK BACKGROUND ➔

PLEASE NOTE THAT THIS CHECKLIST WILL BE PUBLISHED ALONGSIDE YOUR PAPER

Corresponding Author Name: Wen-Chin Yang

Journal Submitted to: EMBO Molecular Medicine

Manuscript Number: EMM-2019-11668V3

Reporting Checklist For Life Sciences Articles (Rev. June 2017)

This checklist is used to ensure good reporting standards and to improve the reproducibility of published results. These guidelines are consistent with the Principles and Guidelines for Reporting Preclinical Research issued by the NIH in 2014. Please follow the journal's authorship guidelines in preparing your manuscript.

A- Figures
1. Data
The data shown in figures should satisfy the following conditions:

- ➔ the data were obtained and processed according to the field's best practice and are presented to reflect the results of the experiments in an accurate and unbiased manner.
- ➔ figure panels include only data points, measurements or observations that can be compared to each other in a scientifically meaningful way.
- ➔ graphs include clearly labeled error bars for independent experiments and sample sizes. Unless justified, error bars should not be shown for technical replicates.
- ➔ if n< 5, the individual data points from each experiment should be plotted and any statistical test employed should be justified
- ➔ Source Data should be included to report the data underlying graphs. Please follow the guidelines set out in the author ship guidelines on Data Presentation.

2. Captions

Each figure caption should contain the following information, for each panel where they are relevant:

- ➔ a specification of the experimental system investigated (eg cell line, species name).
- ➔ the assay(s) and method(s) used to carry out the reported observations and measurements
- ➔ an explicit mention of the biological and chemical entity(ies) that are being measured.
- ➔ an explicit mention of the biological and chemical entity(ies) that are altered/varied/perturbed in a controlled manner.

- ➔ the exact sample size (n) for each experimental group/condition, given as a number, not a range;
- ➔ a description of the sample collection allowing the reader to understand whether the samples represent technical or biological replicates (including how many animals, litters, cultures, etc.).
- ➔ a statement of how many times the experiment shown was independently replicated in the laboratory.
- ➔ definitions of statistical methods and measures:
  - common tests, such as t-test (please specify whether paired vs. unpaired), simple χ2 tests, Wilcoxon and Mann-Whitney tests, can be unambiguously identified by name only, but more complex techniques should be described in the methods section;
  - are tests one-sided or two-sided?
  - are there adjustments for multiple comparisons?
  - exact statistical test results, e.g., P values = x but not P values < x;
  - definition of 'center values' as median or average;
  - definition of error bars as s.d. or s.e.m.

Any descriptions too long for the figure legend should be included in the methods section and/or with the source data.

In the pink boxes below, please ensure that the answers to the following questions are reported in the manuscript itself. Every question should be answered. If the question is not relevant to your research, please write NA (non applicable). We encourage you to include a specific subsection in the methods section for statistics, reagents, animal models and human subjects.

USEFUL LINKS FOR COMPLETING THIS FORM

http://www.antibodypedia.com
http://1degreebio.org
http://www.equator-network.org/reporting-guidelines/improving-bioscience-research-repor

http://grants.nih.gov/grants/olaw/olaw.htm
http://www.mrc.ac.uk/Ourresearch/Ethicsresearchguidance/Useofanimals/index.htm
http://ClinicalTrials.gov
http://www.consort-statement.org
http://www.consort-statement.org/checklists/view/32-consort/66-title

http://www.equator-network.org/reporting-guidelines/reporting-recommendations-for-tumo

http://datadryad.org

http://figshare.com

http://www.ncbi.nlm.nih.gov/gap

http://www.ebi.ac.uk/ega

http://biomodels.net/

http://biomodels.net/miriam/
http://jjj.biochem.sun.ac.za
http://oba.od.nih.gov/biosecurity/biosecurity_documents.html
http://www.selectagents.gov/

B- Statistics and general methods

Please fill out these boxes ➔ (Do not worry if you cannot see all your text once you press return)

1.a. How was the sample size chosen to ensure adequate power to detect a pre-specified effect size?	For animal study, the sample size was 5 and more. For cell experiments and IHC experiments, the sample size was 3 and more.
1.b. For animal studies, include a statement about sample size estimate even if no statistical methods were used.	Three mice per group in Fig.1F; 3 mice per group in Fig.1G (left); 5 mice per group in Fig. 1G (middle); 7 healthy subjects and 5 patients with T2D in Fig. 1G (right); 16 TG db/db , 16 db/db and 26 KO db/db mice in Fig. 2A; 6 WT BKS,5 TG BKS, 7 db/db and 8 TG db/db mice in Fig. 2E;5 WT BKS,5 TG BKS, 6 db/db and 6 TG db/db mice in Fig. 3A; 3 mice per group in Fig. 3B; 5 TG BKS, 8 db/db and 5 TG db/db mice in Fig. 3C and 3D; 3 mice per group in Fig. 4;3 mice per group in Fig. 5; 6 db/db mice per group in Fig. 8.. ANOVA test and log rank test were applied.
2. Describe inclusion/exclusion criteria if samples or animals were excluded from the analysis. Were the criteria pre-established?	Onely newly onset diabetice db/dbd mice were selected for diabeteic treatment (Fig. 8). Mice with FBG (>126 mg/L) or PBG (>200 mg/dL) were considered to be diabetic.
3. Were any steps taken to minimize the effects of subjective bias when allocating animals/samples to treatment (e.g. randomization procedure)? If yes, please describe.	Random grouping of mice
For animal studies, include a statement about randomization even if no randomization was used.	The db/db mice were randomly grouped in Fig. 8. For other animal studies, we needed to group mice based on their genetics backgrounds.
4.a. Were any steps taken to minimize the effects of subjective bias during group allocation or/and when assessing results (e.g. blinding of the investigator)? If yes please describe.	The analysis of our data were double checked by a second investigator.
4.b. For animal studies, include a statement about blinding even if no blinding was done	The mice were randomly grouped or based on their genetic backgrounds. All the mice were ear-marked. Analysis of our animal data were double-checked a second investigator.
5. For every figure, are statistical tests justified as appropriate?	Yes, ANOVA and log rank tests were applied.
Do the data meet the assumptions of the tests (e.g., normal distribution)? Describe any methods used to assess it.	Shapiro-Wilk normality test was used to test the normal distribution.

Is there an estimate of variation within each group of data?	Standard deviation was used to test the estimate of variation within each group.
Is the variance similar between the groups that are being statistically compared?	Bartlett test of homogeneity of variances was used to test the variance between the groups and the variance was similar.

C- Reagents

6. To show that antibodies were profiled for use in the system under study (assay and species), provide a citation, catalog number and/or clone number, supplementary information or reference to an antibody validation profile. e.g., Antibodypedia (see link list at top right), 1DegreeBio (see link list at top right).	anti-Pdia4 (ADI-SPS-720-D), anti-p22phox (sc-20781), anti-Ndufs3 (15066-1-AP), anti-Myc (MA1-980), anti-Flag (TA50011-100), anti-actin (sc-376421), anti-PARP (9542), anti-Na/K ATPase (ST0533), Grp94 (MA3-016), anti-insulin (ab63820), anti-glucagon (M00678-1), anti-Shdb (ab175225), anti-Uqcrfs1 (18443-1-AP), anti-Cox5b (11418-2-AP), anti-Nox2 (19013-1-AP), anti-p47phox (LS-B2365), anti-p67phox (15551-1-AP), and anti-Rac1 (24072-1-AP), anti-Pdia1 (11245-1-AP), anti-Pdia3(15967-1-AP), anti-Pdia6 (18233-1-AP), anti-Gst(sc-138), anti-His (MA1-21315)
7. Identify the source of cell lines and report if they were recently authenticated (e.g., by STR profiling) and tested for mycoplasma contamination.	Min6 (Miyazaki, J.I.) and 293 T cells (ATCC CRL-3216) were authenticated. No mycoplama test was reported.

* for all hyperlinks, please see the table at the top right of the document

D- Animal Models

8. Report species, strain, gender, age of animals and genetic modification status where applicable. Please detail housing and husbandry conditions and the source of animals.	B6, BKS, and B6.BKS(D)-Leprdb/J were purchased from the Jackson laboratory. The conventional knockout, conditional knockout, and transgenic mice of Pdia4 on B6, BKS, and Leprdb/db backgrounds were generated and used in this study. The female mice were used from birth to 120 weeks of age. All animals had free access to chow and water, and were maintained at 21-23℃ with 12 h light-12 h dark cycles in the institutional animal facility.
9. For experiments involving live vertebrates, include a statement of compliance with ethical regulations and identify the committee(s) approving the experiments.	Animal protocol was approved by the Academia Sinica Institutional Animal Care and Utilization Committee (11-03-158).
10. We recommend consulting the ARRIVE guidelines (see link list at top right) (PLoS Biol. 8(6), e1000412, 2010) to ensure that other relevant aspects of animal studies are adequately reported. See author guidelines, under 'Reporting Guidelines'. See also: NIH (see link list at top right) and MRC (see link list at top right) recommendations. Please confirm compliance.	All mice were handled in compliance to the guidelines of the ARRIVE guidelines.

E- Human Subjects

11. Identify the committee(s) approving the study protocol.	The Academia Sinica Institutional Review Board (AS-IRB01-14015).
12. Include a statement confirming that informed consent was obtained from all subjects and that the experiments conformed to the principles set out in the WMA Declaration of Helsinki and the Department of Health and Human Services Belmont Report.	The informed consents obtained from the patients were in compliance to the declaration of Helsinki.
13. For publication of patient photos, include a statement confirming that consent to publish was obtained.	N/A
14. Report any restrictions on the availability (and/or on the use) of human data or samples.	N/A
15. Report the clinical trial registration number (at ClinicalTrials.gov or equivalent), where applicable.	N/A
16. For phase II and III randomized controlled trials, please refer to the CONSORT flow diagram (see link list at top right) and submit the CONSORT checklist (see link list at top right) with your submission. See author guidelines, under 'Reporting Guidelines'. Please confirm you have submitted this list.	N/A
17. For tumor marker prognostic studies, we recommend that you follow the REMARK reporting guidelines (see link list at top right). See author guidelines, under 'Reporting Guidelines'. Please confirm you have followed these guidelines.	N/A

F- Data Accessibility

18: Provide a "Data Availability" section at the end of the Materials & Methods, listing the accession codes for data generated in this study and deposited in a public database (e.g. RNA-Seq data: Gene Expression Omnibus GSE39462, Proteomics data: PRIDE PXD000208 etc.) Please refer to our author guidelines for 'Data Deposition'.<br><br>Data deposition in a public repository is mandatory for:<br>a. Protein, DNA and RNA sequences<br>b. Macromolecular structures<br>c. Crystallographic data for small molecules<br>d. Functional genomics data<br>e. Proteomics and molecular interactions	1. Proteomics data of Pdia4 were deposited in PeptideAtlas (PASS01396). 2. RNAi and cDNA sequences are indicated in Appendix.
19. Deposition is strongly recommended for any datasets that are central and integral to the study; please consider the journal's data policy. If no structured public repository exists for a given data type, we encourage the provision of datasets in the manuscript as a Supplementary Document (see author guidelines under 'Expanded View' or in unstructured repositories such as Dryad (see link list at top right) or Figshare (see link list at top right).	Already deposited
20. Access to human clinical and genomic datasets should be provided with as few restrictions as possible while respecting ethical obligations to the patients and relevant medical and legal issues. If practically possible and compatible with the individual consent agreement used in the study, such data should be deposited in one of the major public access-controlled repositories such as dbGAP (see link list at top right) or EGA (see link list at top right).	N/A
21. Computational models that are central and integral to a study should be shared without restrictions and provided in a machine-readable form. The relevant accession numbers or links should be provided. When possible, standardized format (SBML, CellML) should be used instead of scripts (e.g. MATLAB). Authors are strongly encouraged to follow the MIRIAM guidelines (see link list at top right) and deposit their model in a public database such as Biomodels (see link list at top right) or JWS Online (see link list at top right). If computer source code is provided with the paper, it should be deposited in a public repository or included in supplementary information.	N/A

G- Dual use research of concern

22. Could your study fall under dual use research restrictions? Please check biosecurity documents (see link list at top right) and list of select agents and toxins (APHIS/CDC) (see link list at top right). According to our biosecurity guidelines, provide a statement only if it could.	N/A
